# Comparative Expression Profiling of *Leishmania*: Modulation in Gene Expression between Species and in Different Host Genetic Backgrounds

**DOI:** 10.1371/journal.pntd.0000476

**Published:** 2009-07-07

**Authors:** Daniel P. Depledge, Krystal J. Evans, Alasdair C. Ivens, Naveed Aziz, Asher Maroof, Paul M. Kaye, Deborah F. Smith

**Affiliations:** 1 Centre for Immunology and Infection, Department of Biology/Hull York Medical School, University of York, York, United Kingdom; 2 Fios Genomics, ETTC, King's Buildings, Edinburgh, United Kingdom; 3 Technology Facility, Department of Biology, University of York, York, United Kingdom; Louisiana State University, United States of America

## Abstract

**Background:**

Genome sequencing of *Leishmania* species that give rise to a range of disease phenotypes in the host has revealed highly conserved gene content and synteny across the genus. Only a small number of genes are differentially distributed between the three species sequenced to date, *L. major*, *L. infantum* and *L. braziliensis*. It is not yet known how many of these genes are expressed in the disease-promoting intracellular amastigotes of these species or whether genes conserved between the species are differentially expressed in the host.

**Methods/Principal Findings:**

We have used customised oligonucleotide microarrays to confirm that all of the differentially distributed genes identified by genome comparisons are expressed in intracellular amastigotes, with only a few of these subject to regulation at the RNA level. In the first large-scale study of gene expression in *L. braziliensis*, we show that only ∼9% of the genes analysed are regulated in their RNA expression during the *L. braziliensis* life cycle, a figure consistent with that observed in other *Leishmania* species. Comparing amastigote gene expression profiles between species confirms the proposal that *Leishmania* transcriptomes undergo little regulation but also identifies conserved genes that are regulated differently between species in the host. We have also investigated whether host immune competence influences parasite gene expression, by comparing RNA expression profiles in *L. major* amastigotes derived from either wild-type (BALB/c) or immunologically compromised (Rag2^−/−^ γ_c_^−/−^) mice. While parasite dissemination from the site of infection is enhanced in the Rag2^−/−^ γ_c_^−/−^ genetic background, parasite RNA expression profiles are unperturbed.

**Conclusion/Significance:**

These findings support the hypothesis that *Leishmania* amastigotes are pre-adapted for intracellular survival and undergo little dynamic modulation of gene expression at the RNA level. Species-specific parasite factors contributing to virulence and pathogenicity in the host may be limited to the products of a small number of differentially distributed genes or the differential regulation of conserved genes, either of which are subject to translational and/or post-translational controls.

## Author Summary

The single-celled parasite *Leishmania*, transmitted by sand flies in more than 88 tropical and sub-tropical countries globally, infects man and other mammals, causing a spectrum of diseases called the leishmaniases. Over 12 million people are currently infected worldwide with 2 million new cases reported each year. The type of leishmaniasis that develops in the mammalian host is dependent on the species of infecting parasite and the immune response to infection (that can be influenced by host genetic variation). Our research is focused on identifying parasite factors that contribute to pathogenicity in the host and understanding how these might differ between parasite species that give rise to the different clinical forms of leishmaniasis. Molecules of this type might lead to new therapeutic tools in the longer term. In this paper, we report a comparative analysis of gene expression profiles in three *Leishmania* species that give rise to different types of disease, focusing on the intracellular stages that reside in mammalian macrophages. Our results show that there are only a small number of differences between these parasite species, with host genetics playing only a minor role in influencing the parasites' response to their intracellular habitat. These small changes may be significant, however, in determining the clinical outcome of infection.

## Introduction

Infection with species of the kinetoplastid parasite, *Leishmania*, results in a spectrum of diseases in man, termed the leishmaniases [1],[2]. These range from the non-fatal chronic cutaneous lesions arising from *L. major* infection to mucocutaneous leishmaniasis usually associated with *L. braziliensis* (classified within the sub-genus *L. Viannia*) and the often fatal visceralising disease, most commonly associated with *L. donovani* infection in the Indian sub-continent, *L. chagasi* in Brazil and *L. infantum* in the Mediterranean basin. (The last two species are generally considered to be genetically identical [3]). While the species of infecting parasite can play a defining role in disease type, the genetic background and immune response of the host are also major factors in determining clinical outcome [4],[5],[6],[7],[8],[9]. Understanding the relative contribution of these different components may enhance our understanding of pathogenicity in the leishmaniases.

Sequencing and comparison of the genomes of representative lab-adapted strains of *L. major*, *L. infantum* and *L. braziliensis* have revealed strong conservation of gene content and synteny, with only a small number of genes identified as differentially distributed between species [1],[10]. This subset of genes, together with sequences preferentially expressed in intracellular amastigotes and/or showing differential expression between species, may be important in facilitating parasite survival and maintenance within the host. The best-characterised example of the former class is the *L. donovani* complex-specific A2 gene coding for an amastigote protein of as yet unknown function which, when expressed in *L. major*, leads to increased parasite dissemination to the viscera [11].

Recent expression profiling has identified 3–9% of genes that are modulated at the RNA level between life cycle stages of several *Leishmania* species [12],[13],[14],[15]. Moreover, comparisons of *L. mexicana* amastigote parasites grown axenically with those maintained within macrophages, either *in vitro* or *in vivo*, have shown that axenic (extracellular) amastigotes are more similar to extracellular promastigotes than to macrophage-derived (intracellular) amastigotes in their RNA profiles [12]. These observations emphasise the importance of using parasites isolated *ex vivo* to investigate the mechanisms of intracellular survival. To date, no comparative expression profiling has been performed on *L. Viannia spp.*, despite the relative divergence of the genome of *L. braziliensis* from that of *L. major* or *L. infantum* [1].

A complicating factor in the analysis of *Leishmania* gene expression is the almost complete absence of defined RNA polymerase II promotors in kinetoplastid species, coupled with the characteristic bidirectional polycistronic transcription units found on individual chromosomes [16],[17],[18]. In these organisms, polycistronic precursor RNAs (which may be expressed constitutively) are processed by coupled *trans*-splicing and polyadenylation [19] to generate mature mRNA transcripts for translation. Expression of individual genes is regulated post-transcriptionally, a process largely dependent on RNA stabilisation mechanisms [17],[18]. This post-transcriptional regulation, coupled with an as yet unknown extent of regulation at the level of translation, results in variable correlations between gene and protein expression levels [20],[21]. Such factors place greater emphasis on identifying those genes which undergo regulation during the life cycle while also looking for differences in the relative levels of expression of conserved genes.

Given the importance of intracellular *Leishmania* stages to disease in man, this study has focused on amastigote gene expression, comparing RNA expression profiles between the three sequenced *Leishmania* species to identify any significant differences that may be functionally relevant in these infective parasite stages. To achieve this end, an oligonucleotide array was designed representative of 4 functional classes of genes, together comprising ∼10% of the genome. These targets included (i) all genes identified as differentially-distributed between the 3 sequenced *Leishmania* species [1]; (ii) all genes containing amino acid repeats within their open reading frames [22]; (iii) all genes encoding proteins predicted (with high probability) to be co-translationally modified by *N*-myristoylation [23]; (iv) a range of control genes of known RNA expression profiles.

The rationale for this choice of target genes is that their expressed products provide a subset for analysis as putative targets for therapeutic intervention. 2–4% of the 3 sequenced *Leishmania* genomes, including 30% of the differentially-distributed genes, contain amino acid repeats within their protein-coding regions, as identified by the RepSeq web utility [22]. Repeat domains are associated with a range of functions relevant to host survival in other protozoan parasites, including antigenic variation, host-cell receptor binding and intracellular protein-protein interactions in *Plasmodium* species [24],[25],[26]. Amino acid repeat-containing proteins encoded within the *Leishmania* genomes include several kinases, cysteine peptidases, putative surface antigen proteins and the infective stage-specific HASPB (formerly named GBP), expressed in amastigotes of all *L. Leishmania* species analysed but absent from *L.(Viannia.) braziliensis* [1],[27],[28],[29],[30]. HASPB is also a target for *N*-myristoylation, a co-translational protein modification catalysed by the enzyme *N*-myristoyltransferase (NMT) that is a genetically-validated target for *Leishmania* drug development [31],[32].

Hybridisation of these customised oligonucleotide arrays with amastigote RNA derived from footpad lesions (*L. major*), spleens (*L. infantum*) and RAW 264.7 macrophages (*L. braziliensis*) revealed that only a small number of the target genes are differentially expressed at the mRNA level between species and that fewer still are regulated during the parasite life cycle. In addition, comparison of expression profiles derived from *L. major* amastigotes isolated from hosts of differing immune competence (BALB/c vs. Rag2^−/−^ γ_c_^−/−^ mice [33]) demonstrated that host immune pressure has little effect on parasite gene expression at the RNA level. Taken as a whole, the data presented here suggest that parasites do not respond dynamically to host immune pressure and that any influence of varying transcript levels on virulence and pathogenicity of different *Leishmania* species is likely to result from the differential expression of conserved genes between species and/or the expression of a small number of genes that are differentially distributed between species.

## Materials and Methods

### In silico analyses

The three representative *Leishmania* proteomes (*L .major*, *L. braziliensis* and *L. infantum*) were analysed for the presence of amino acid repeats using default settings at the RepSeq web utility (www.repseq.org; [22]); 256 proteins were identified. A further 62 proteins were predicted to be *N*-terminally myristoylated with high confidence using the NMT Predictor and Myristoylator programs as described [23]. The genes identified as differentially distributed between the three *Leishmania* species [1] were reanalysed using BLAST and the latest assemblies of the three genomes available at the time on GeneDB (*L. major* published assembly [10], *L. infantum* v2 and *L. braziliensis* v1). 242 of these genes were included in this study (Table S1); the remainder have been removed during annotation revision.

### Leishmania culture, differentiation and in vivo infections

The three sequenced genome reference strains used were *L. major* MHOM/IL/80/Friedlin; *L. infantum* clone JPCM5 MCAN/ES/98/LLM-877; *L. braziliensis* clone M2904 MHOM/BR/75M2904. Procyclic and metacyclic parasites were maintained at 26°C in pH 7.0 and pH 5.5 media respectively; for *L. major* and *L. braziliensis*, 1×M199 medium was supplemented with 10% Gibco fetal bovine serum (FBS, Invitrogen) and penicillin-streptomycin (Invitrogen) and, in the case of *L. braziliensis*, 2% human male urine. *L. infantum* was cultured in Gibco HOMEM (Invitrogen) supplemented with 10% FBS (Biosera) and penicillin-streptomycin. The pH of all media was adjusted using orthophosphoric acid. Metacyclic RNAs were validated by RT-qPCR profiling of the metacyclic specific markers SHERP [34] in *L. major* and *L. infantum* and Meta1 [35] in *L. braziliensis*. For *L. major in vivo* passage, BALB/c and Rag2^−/−^γ_c_^−/−^ mice [33],[36] were infected sub-cutaneously in the rear footpad with 4×10^6^ late stage *L. major* metacyclics purified by Percoll gradient fractionation [37]. Footpad thickness was measured weekly using digital calipers, taking an average of three readings. At four weeks post-infection, mice were euthanized and tissues were sampled for analysis. For *L. infantum in vivo* passage, infection was initiated in hamsters by intraperitoneal inoculation of 10^7^ stationary phase metacyclics as described [38], with euthanasia and spleen removal 9–12 months later.

BALB/c mice (Charles River Laboratories) and Rag2^−/−^γ_c_^−/−^ mice (bred in the Centre for Immunology and Infection) were housed in pathogen-free conditions at the University of York. All animal work was conducted under UK Home Office Licence requirements and after institutional ethical review. Attempts to purify amastigotes from the footpads of BALB/c, C57BL/6 and Rag2^−/−^γ_c_^−/−^ mice infected with the *L. braziliensis* genome strain were unsuccessful (despite establishment of lymph nodes infections with the same parasites), so Percoll-purified metacyclics were used to infect RAW 264.7 macrophages maintained in DMEM medium supplemented with 10% FBS, l-glutamine and sodium pyruvate (1 mM final concentrations). Amastigotes were obtained from infected macrophages after 3 days culture at 34°C. For all species, infections were initiated with parasites that had been recently passaged *in vivo* to reduce any impact of long term *in vitro* culture on parasite gene expression and virulence.

### Amastigote isolation and protein analysis

Infected tissues were homogenised and fractionated (centrifugation at 100 g, 4°C) to remove larger debris. Host cells in the resulting supernatant were pelleted (2000 g, 4°C) and treated with 0.05% w/v saponin (Sigma Aldrich) for lysis and release of amastigotes. Host cell debris was removed by layering parasites over Percoll (1.037 g/ml) and centrifugation at 2000 g, 4°C for 1 hr. Amastigote yields were assessed using a haemocytometer and Beckman Z Series Coulter Counter. Parasite species was verified by restriction fragment length polymorphism analysis of amastigote DNA [39].

For immunoblotting, 5×10^6^ host-derived amastigotes were pelleted, washed in PBS and resuspended in an SDS load mix supplemented with Pepstatin A (50 ug/ml). Treatment with 3% β-mercaptoethanol was followed by a 10 min incubation at 95°C prior to 12% SDS-PAGE, electroblotting on immobilon-P membrane (Millipore) and probing with anti-HASPB [40],[41] at 1∶1250 dilution and anti-NMT [31] at 1∶1000, as previously described. ECL Western Blotting Detection Reagents (GE Healthcare Lifesciences) were used in conjunction with a G∶BOX Chemi Imaging System and GeneSnap V.7.0 software (Syngene) to determine relative expression of detected proteins.

### Analysis of parasite burdens

*L. major* parasite burdens in mouse footpad lesions were determined by purification and manual counting while those in the liver and spleen tissues were assessed from duplicate samples using quantitative PCR, adapted from the method of [42]. DNA was extracted from tissues using a DNeasy Blood and Tissue kit (Qiagen) following overnight digestion with Proteinase K at 55°C. Quantitation of *L. major* DNA was performed using primers specific for a 116-bp fragment of the kinetoplastid minicircle DNA: forward, 5′-CCTATTTTACACCAACCCCCAGT-3′(JW11) and reverse, 5′-GGGTAGGGGCGTTCTGCGAAA-3′ (JW12) [43]. DNA standards were created for each tissue sampled, by spiking naïve tissue with known numbers of *L. major* promastigotes and extracting the DNA as described above. For RT-qPCR reactions, samples were analysed using Power SYBR Green PCR Master Mix and an ABI 7300 sequence analyzer (Applied Biosystems). Samples were subjected to an initial holding step at 95°C for 10 min, followed by an amplification step (40 cycles of 95°C for 15 sec and 60°C for 1 min) and a single dissociation step of 95°C for 15 sec, 60°C for 20 sec and 95°C for 15 sec. The total parasite burden per organ was calculated by determining the total number of parasites per g of tissue sampled and multiplying by total organ mass.

### RNA isolation, cDNA synthesis and labelling

Amastigote pellets were resuspended in TRIzol Reagent (Invitrogen) at a concentration of 1 ml/10^8^ cells and total RNA extracted using the protocol provided. Extracted RNA was purified using a MEGAclear kit (Ambion) and the quantity and quality verified using a NanoDrop ND-1000 Spectrophotometer and Agilent 2100 Bioanalyzer, respectively, prior to storage at −80°C. cDNA synthesis and subsequent labelling was performed using the Amino Allyl cDNA labeling kit (Ambion) and Alexa Flour 555 and 647 dyes (Invitrogen).

### DNA oligonucleotide array design, slide printing, washing and pre-hybridisation

70-mer oligonucleotides were designed and synthesised by Operon Biotechnologies using parameters such that a single oligo had >90% identity with the gene target in each of the three species but <70% identity with any other gene on the array. All oligos (Table S2) were BLAST-searched against the NCBI databases for the mouse and hamster genomes, to minimise potential cross hybridisation. The microarrays were printed on Nexterion Slides E (Schott) using a QArrayMini (Genetix) as follows: 5 replicates were printed for each probe in a random pattern totalling 5376 probes per slide (including buffer and negative controls). cDNA from procyclic RNA from each of the three species was hybridised against the arrays for quality control, revealing that all probes were hybridised (data not shown). Prior to hybridisation, each slide was washed at room temperature as follows: 1×5 min in 0.1% Triton X-100, 2×2 min in 1 mM HCL, 1×10 min in 100 mM KCL. Slides were subsequently blocked by incubation in 1×Nexterion Blocking E (Schott) for 15 min and then dried by centrifugation.

### Microarray hybridisation and signal detection

Labelled cDNA pellets were resuspended in Nexterion Hyb and hybridised on the slides in Corning hybridisation chambers (Fisher) for 16 hrs at 45°C. The slides were then washed in 2×Sodium Chloride Citrate (SSC)/0.1% sodium dodecyl sulphate (SDS) for 5 min, 2×SSC for a further 5 min, 0.2×SSC for 5 min and dried by centrifugation at 200 g for 3 min. The slides were scanned using an Axon 4000A scanner and fluorescence signal intensities of the array features and local backgrounds measured by GenePix Pro 5.0.

### Data processing and statistical analysis

For each slide, flags were automatically assigned to each probe and manually evaluated, based on the quality of hybridisation, to assist in normalisation within and between arrays. All raw data extracted from GenePix were imported into R 2.2.1 for normalization and statistical analysis using the Bioconductor packages (www.bioconductor.org), predominantly Limma. Briefly, gpr files were read and background correction performed using the Kooperberg model. QC analysis, entailing density distribution analysis coupled with correlation distance heatmap plotting, was used to identify substandard arrays, which were removed from further analysis. Array data were normalised within arrays using printtiploess, followed by normalisation between arrays using the “Aquantile” method, which normalises intensity distribution across all slides. Subsequently, the mean intensity was determined for the 5 replicate spots on each array prior to single channel linear modelling and contrast fitting of non-control array features, with eBayes methods to generate moderated t-statistics. Four contrasts were performed: *L. braziliensis* procyclics vs. metacyclics vs. amastigotes, *L. major* amastigotes vs. procyclics, *L. infantum* amastigotes vs. procyclics and *L. major* amastigotes from BALB/c mice vs. *L. major* amastigotes from Rag2^−/−^γ_c_^−/−^ mice.

Gene lists were generated, with accompanying statistics, from the eBayes fit data. The decideTests method was applied, with an adjust p value threshold of 0.01, to classify genes as to their significance in one of more contrasts. A fold change of 1.7 was then used as threshold for determining differentially expressed genes, similar to other recent studies [13],[14],[20]. In accordance with MIAME (Minimum Information About a Microarray Experiments) regulations [44], all data were deposited into ArrayExpress database [45] at www.ebi.ac.uk under the accession number E-MEXP-2063.

For host-derived amastigote analysis, a minimum of 3 biological replicates (each comprising 2–3 technical replicates) per species were used. 2–3 technical replicates were also used for each of the following analyses: culture-derived amastigotes, metacyclics and procyclics

### Quantitative real-time PCR (RT-qPCR)

RT-qPCR was performed on selected genes to validate the microarray data, using 1–2 biological replicates (independent of those used on the arrays). cDNA was synthesised using the Omniscript RT Kit (Qiagen) . Cross-species primers were designed by aligning gene sequences using CLUSTALW (http://align.genome.jp/) and then selecting primers based on the output of Primaclade [46]. Each biological sample was run with 4–5 technical replicates. Data generated were analysed [47] and normalised using a range of suitable targets (including published controls; [14],[35],[48]). All primers used are shown in Table S3.

## Results/Discussion

Previous expression profiling studies, using arrays of varying size and composition, have revealed that the transcriptomes of four *L. Leishmania* species (*L. major*, *L. infantum*, *L. donovani and L mexicana*; [12],[13],[14],[15],[49],[50],[51]) are largely expressed at constitutive levels during the parasite life cycle, with only a limited number of genes (<10% of the genome) showing significant evidence of regulation (fold change >1.7, p<0.05) at the mRNA level. No data have yet been presented on global expression profiles of any *L. Viannia spp*, nor has there been an investigation into the potential influence of immune pressure on parasite gene expression in the host.

The custom microarrays used in this study targeted 785 genes representing ∼10% of the genome of the 3 target *Leishmania* species, *L. major*, *L. infantum* and *L. braziliensis*. Following hybridisation, a total of 46 genes were selected from the arrays for validation of their RNA expression profiles by RT-qPCR. These included a number of previously-characterised stage-regulated and constitutively-expressed genes across all three species. In total, 74% (17/23) of the *L. braziliensis* genes tested for their expression across the parasite life cycle showed good correlation between RNA levels detected by RT-qPCR and microarray analysis (Table 1). A further 8 out of 9 targets from the cross-species comparison and 12 out of 14 targets selected from the study of host immune pressure on *L. major* gene expression were also validated by comparison of RT-qPCR and microarray data (Table 1). In most cases, the observed fold changes were comparable between the microarray and RT-qPCR data although in some instances, larger differences between the two methods of analysis were measured. This trend has been reported in similar studies [12],[14] and is likely to be due to the increased sensitivity and specificity of RT-qPCR against single gene targets.

**Table 1:** Validation of microarray data by quantitative RT-PCR (RT-qPCR).

*L. braziliensis* lifecycle			*L. braziliensis* vs *L. infantum* amastigotes			
GeneDB ID	Array LogFC	RT-qPCR LogFC	*L. braziliensis* ID	*L. infantum* ID	Array LogFC	RT-qPCR LogFC
LbrM04_V2.0370	2.78	−1.45*	LbrM03_V2.0240	LinJ03_V3.0220	−5.82	−0.83
LbrM07_V2.0360	−1.11	−1.22	LbrM08_V2.0810	LinJ08_V3.0960	2.22	5.34
LbrM09_V2.0960	−1.04	−2.94	LbrM16_V2.0920	LinJ16_V30920	2.53	291.62
LbrM11_V2.0560	−2.80	−1.63				
LbrM12_V2.0750	−2.31	−0.84	***L. major*** ** vs ** ***L. infantum***			
LbrM21_V2.0360	2.51	0.55*	***L. major*** ** ID**	***L. infantum*** ** ID**	**Array LogFC**	**RT-qPCR LogFC**
LbrM26_V2.0120	0.84	1.64	LmjF03.0230	LinJ03_V3.0220	−5.29	−10.43
LbrM31_V2.2570	−0.89	−2.90	LmjF13.0880	LinJ13_V3.0770	−2.68	−2.66
LbrM31_V2.3150	1.91	−1.09*	LmjF29.1480	LinJ29_V3.1580	0.01	9.49*
LbrM34_V2.4130	−3.15	−2.71	LmjF31.3000	LinJ31_V3.3110	−1.83	−1.87
LbrM08_V2.0810	1.95	−0.57*	LmjF32.1950	LinJ32_V3.2060	0.01	1.21
LbrM09_V2.0840	1.19	2.54	LmjF36.4310	LinJ36_V3.4520	2.88	0.28
LbrM09_V2.0960	−0.82	−0.46				
LbrM11_V2.0560	−2.51	−0.18	***L. major*** ** amastigotes from BALB/c and Rag2^−/−^γ_c_^−/−^ mice**			
LbrM20_V2.0550	2.48	1.01	***L. major*** ** ID**	**Array LogFC**	**RT-qPCR LogFC**	
LbrM31_V2.3150	2.08	−1.84*	LmjF03.0230	0.50	0.05	
LbrM04_V2.0370	−3.67	2.55*	LmjF04.0210	−0.17	0.44*	
LbrM08_V2.0810	1.67	1.09	LmjF24.0220	−0.25	−0.12	
LbrM12_V2.0750	3.08	4.16	LmjF24.1840	1.35	0.43	
LbrM18_V2.0050	1.62	−1.07*	LmjF25.1820	−0.03	−0.18	
LbrM20_V2.0550	2.46	1.99				
LbrM32_V2.2500	0.79	3.66				
LbrM34_V2.4130	3.47	1.41				

*: **RT**-qPCR data does not validate microarray data; LogFC represents −log_2_(fold change); ID, each ID refers to the GeneDB gene entry.

### Only a small number of the genes analysed are regulated in their expression during the *L. braziliensis* lifecycle

**Figure 1:**
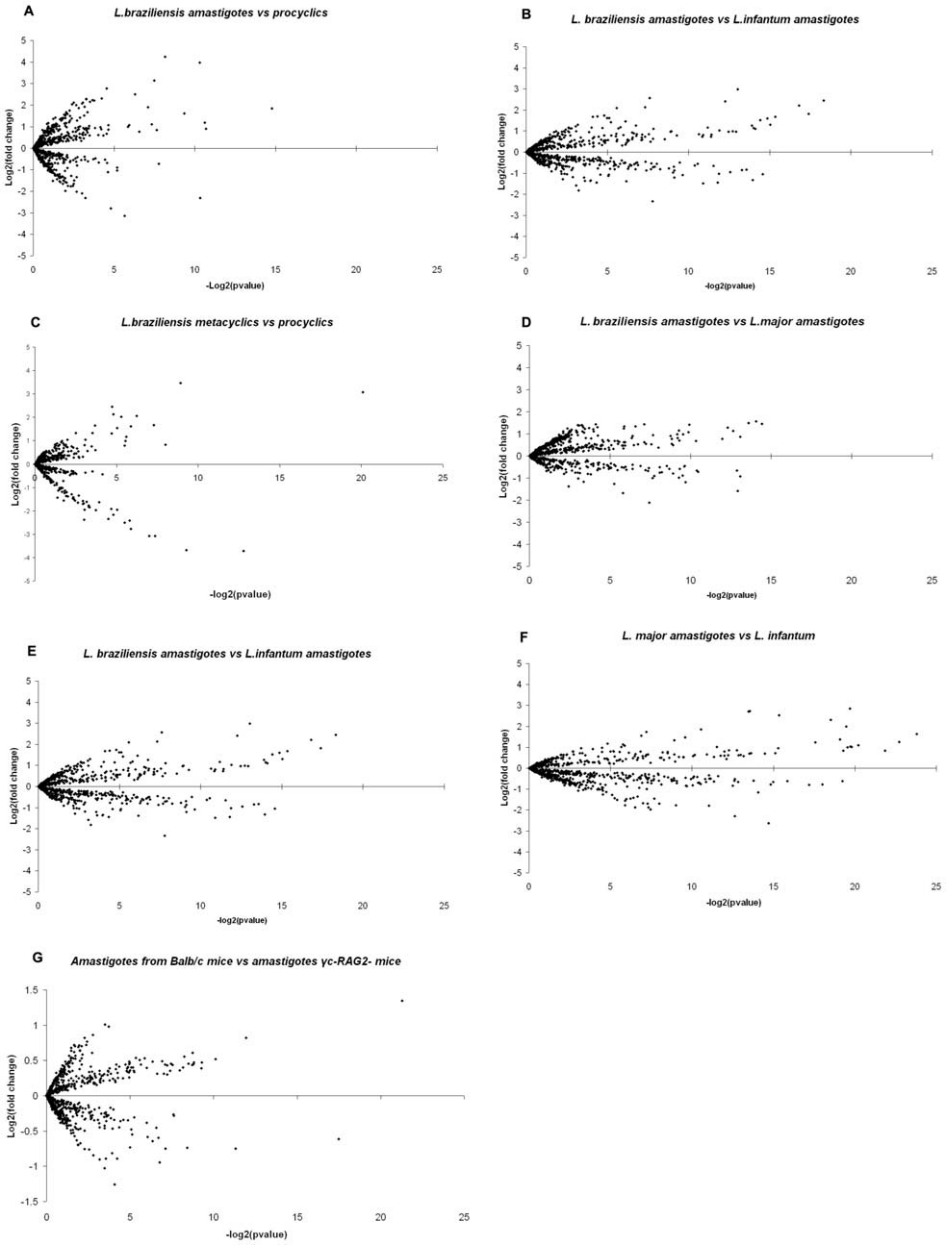
Expression profiling of gene subsets from three *Leishmania* genomes. Scatter plots (generated using Microsoft Excel) show the distribution of log_2_ fold changes in RNA expression levels against their statistical significance (−log_2_ p-value) for up to 785 genes per genome. Fold change of >1.7 between data points (dashed horizontal lines); significant p-value<0.05 (dashed vertical lines). (A) *L. braziliensis* amastigotes vs. procyclics; (B) *L. braziliensis* amastigotes vs. metacyclics; (C) *L. braziliensis* metacyclics vs. procyclics; amastigotes of (D) *L. braziliensis* vs. *L. major*, (E) *L. braziliensis* vs. *L. infantum*, (F) *L. major* vs. *L. infantum*; (G) *L. major* amastigotes from BALB/c vs. Rag2^−/−^γ_c_^−/−^ mice.

Expression profiles were generated for each *L. braziliensis* life cycle stage using mRNA from culture-derived procyclics and metacyclics and RAW 264.7 macrophage-derived amastigotes. The profiles were analysed using scatter plots (Figure 1A–C), revealing that ∼9% (60/678) of the genes probed were significantly regulated (fold change >1.7, p-value<0.05) in their expression between life cycle stages. Thus, although the arrays used for this analysis of *L. braziliensis* gene expression represent only 10% of the genome (and are biased towards specific gene subsets), the proportion of stage-regulated genes (∼9%) is of the same order of magnitude as that observed in whole genome analysis of *L. major* (7%), *L. mexicana* (3.5%) and *L. infantum* (9.3%) [12],[14].

**Figure 2:**
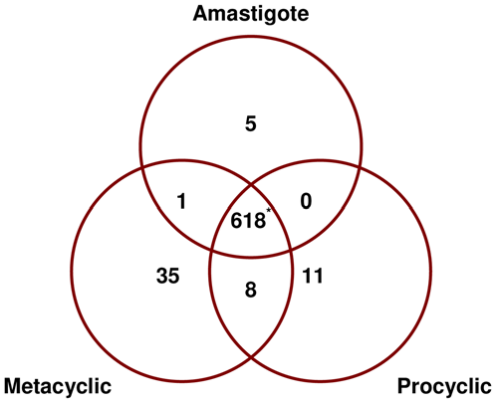
Distribution of genes preferentially expressed in one or more stages of *L. braziliensis*. Venn diagram showing distribution of upregulated genes (>1.7 fold, p<0.05) in stages of the *L. braziliensis* life cycle. 8.8% (60/678) of the genes probed are regulated in their expression by these criteria, with 58% of these showing increased expression during the metacyclic stage. *Genes that are not differentially expressed between these stages.

Of the 60 regulated genes in *L. braziliensis* (Table 2, Figure 2), 5 were preferentially expressed in amastigotes (LbrM05_V2.0380 - microtubule-associated protein, LbrM07_V2.0360 - ATP-dependent DEAD/H RNA helicase, LbrM09_V2.0960 - calmodulin, LbrM11_V2.0560 - 40S ribosomal protein S21 and LbrM31_V2.2570 - 3′ nucleotidase/nuclease) with a further 35 preferentially expressed in metacyclics and 11 in procyclics. Analysis of the specific gene subsets identified none of the NMT targets as upregulated in amastigotes while 5 of these genes showed preferential expression in either procyclics and/or metacyclics. 9.8% (25/256) of the genes coding for proteins containing amino acid repeats showed stage specific regulation, with 3 of these encoding proteins of unknown function that are preferentially expressed in the procyclic and metacyclic stages (as compared with amastigotes). Interestingly, all of the genes preferentially expressed in *L. braziliensis* amastigotes in this analysis, with the exception of the calmodulin array, contain repeat motifs. If these RNA differences correlate with protein levels, this result could indicate that repeat-containing proteins are functionally important in the intracellular stage of the life cycle and provide a useful sub-set of biomarkers with diagnostic and vaccination potential.

**Table 2:** Genes differentially expressed during the *L. braziliensis* lifecycle.

*L. braziliensis*: Amastigotes vs. Procyclics					
GeneDB ID	Product	LogFC	P value	Note	Up in
LbrM34_V2.4130	Poly A binding protein	−3.15	0.0197	SRR*	Amastigote
	40S ribosomal protein S21	−2.80	0.0359	SRR*	Amastigote
LbrM12_V2.0750	Surface antigen proteins (1 and 2)	−2.31	0.0008	SRR*	Amastigote
LbrM07_V2.0360	ATP-dependent DEAD/H RNA helicase	−1.11	0.0405	SRR*	Amastigote
LbrM09_V2.0960/LbrM09_V2.0970/LbrM09_V2.0980	Calmodulin	−1.04	0.0274	*	Amastigote
LbrM31_V2.2570§	3′ nucleotidase/nuclease	−0.89	0.0272	*	Amastigote
LbrM26_V2.0120	Adenine phosphoribosyltransferase	0.84	0.0050	*	Procyclic
LbrM22_V2.1410§	Unknown function	0.86	0.0398		Procyclic
LbrM27_V2.1350§	Carboxypeptidase	0.99	0.0172		Procyclic
LbrM33_V2.0190	Unknown function	1.08	0.0397		Procyclic
LbrM11_V2.0160	Unknown function	1.08	0.0163		Procyclic
LbrM20_V2.4280	Unknown function	1.11	0.0062		Procyclic
LbrM12_V2.0750	Surface antigen proteins (1 and 2)	1.20	0.0006	SRR	Procyclic
LbrM34_V2.0500	Unknown function	1.84	0.0000		Procyclic
LbrM31_V2.3150	Adp-ribosylation factor	1.91	0.0073	NMT*	Procyclic
LbrM21_V2.0360	Unknown function	2.51	0.0126	NMT*	Procyclic
LbrM04_V2.0370	Adp-ribosylation factor	2.78	0.0425	NMT*	Procyclic
LbrM30_V2.1290	Unknown function	3.98	0.0008		Procyclic
LbrM31_V2.0450	Unknown function	4.25	0.0035		Procyclic

*L. braziliensis*: Amastigotes vs. Metacyclics					
GeneDB ID	Product	LogFC	P value	Note	Up in
LbrM11_V2.0560	40S ribosomal protein S21	−2.51	0.0034	SRR*	Amastigote
LbrM12_V2.0750^§§^	Surface antigen proteins (1 and 2)	−2.08	0.0215	SRR	Amastigote
LbrM05_V2.0380	Microtubule-associated protein	−1.11	0.0454		Amastigote
LbrM09_V2.0960/LbrM09_V2.0970/LbrM09_V2.0980	Calmodulin	−0.82	0.0063	*	Amastigote
LbrM34_V2.0360	Serine palmitoyltransferase	0.82	0.0184		Metacyclic
LbrM04_V2.1260	Surface antigen-like protein	0.86	0.0252	SRR	Metacyclic
LbrM27_V2.1350§	Carboxypeptidase	0.86	0.0014		Metacyclic
LbrM33_V2.0190	Unknown function	0.90	0.0071		Metacyclic
LbrM19_V2.1010	Kinesin	0.91	0.0001	SRR	Metacyclic
LbrM35_V2.5860	G-actin binding protein	0.91	0.0269		Metacyclic
LbrM11_V2.0160	Unknown function	0.96	0.0010		Metacyclic
LbrM08_V2.1090/LbrM08_V2.1080/LbrM08_V2.1100	Amastin-like protein	0.98	0.0033		Metacyclic
LbrM34_V2.5330	Unknown function	0.99	0.0107		Metacyclic
LbrM07_V2.0890	Unknown function	1.11	0.0253		Metacyclic
LbrM20_V2.4280	Unknown function	1.11	0.0000		Metacyclic
LbrM33_V2.1190	Unknown function	1.17	0.0037		Metacyclic
LbrM24_V2.1790	Protein kinase	1.18	0.0068	NMT	Metacyclic
LbrM04_V2.0590	Unknown function	1.18	0.0075		Metacyclic
LbrM09_V2.0840	Unknown function	1.19	0.0005	SRR*	Metacyclic
LbrM06_V2.0760	RNA-binding protein-like pr	1.20	0.0016	SRR	Metacyclic
LbrM25_V2.0950	Unknown function	1.28	0.0035	SRR	Metacyclic
LbrM22_V2.1410§	Unknown function	1.30	0.0000		Metacyclic
LbrM07_V2.0550	Unknown function	1.30	0.0006		Metacyclic
LbrM28_V2.3180§	Unknown function	1.32	0.0224		Metacyclic
LbrM32_V2.1080	Unknown function	1.39	0.0250		Metacyclic
LbrM24_V2.0360/LbrM24_V2.0440	CMP-sialic acid transporter	1.40	0.0291		Metacyclic
LbrM33_V2.3400	Unknown function	1.44	0.0252		Metacyclic
LbrM23_V2.0100	Unknown function	1.50	0.0472	SRR	Metacyclic
LbrM31_V2.3260	Nucleoporin (NUP54/57)	1.50	0.0266	SRR	Metacyclic
LbrM04_V2.0550	Unknown function	1.53	0.0434	SRR	Metacyclic
LbrM15_V2.0460/LbrM15_V2.0470/LbrM15_V2.0480	Unknown function	1.55	0.0119		Metacyclic
LbrM04_V2.0470§	Unknown function	1.70	0.0090		Metacyclic
LbrM21_V2.2150/LbrM33_V2.0920/LbrM33_V2.0930/LbrM33_V2.0960	Beta-tubulin	1.71	0.0059		Metacyclic
LbrM18_V2.0050§	Transporter (sugar)	1.95	0.0004		Metacyclic
LbrM08_V2.0810/LbrM08_V2.0820/LbrM08_V2.0830	Cathepsin L-like protease	1.95	0.0001	*	Metacyclic
LbrM27_V2.2760	Peptidyl dipeptidase	1.97	0.0211		Metacyclic
LbrM30_V2.1290	Unknown function	2.03	0.0044		Metacyclic
LbrM31_V2.3150	Adp-ribosylation factor	2.08	0.0000	NMT*	Metacyclic
LbrM34_V2.0500	Unknown function	2.15	0.0000		Metacyclic
LbrM21_V2.0360	Unknown function	2.25	0.0006	NMT	Metacyclic
LbrM30_V2.2320	Adp-ribosylation factor-like protein	2.28	0.0113		Metacyclic
LbrM20_V2.0550	Unknown function	2.48	0.0079	SRR*	Metacyclic

*L. braziliensis*: Procyclics vs Metacyclics					
GeneDB ID	Product	LogFC	P value	Note	Up in
LbrM04_V2.0370	Adp-ribosylation factor	−3.67	0.0016	NMT	Procyclic
LbrM16_V2.0790	Unknown function	−3.08	0.0061		Procyclic
LbrM31_V2.0450	Unknown function	−3.08	0.0078		Procyclic
LbrM05_V2.0960	Unknown function	−2.77	0.0171		Procyclic
LbrM31_V2.1250	Unknown function	−2.50	0.0223	SRR	Procyclic
LbrM15_V2.0940	Unknown function	−2.42	0.0180	SRR	Procyclic
LbrM34_V2.0010/LbrM34_V2.0040/LbrM34_V2.0060	Pyruvate kinase	−2.35	0.0444		Procyclic
LbrM32_V2.3450	Unknown function	−2.17	0.0356		Procyclic
LbrM30_V2.1290	Unknown function	−1.95	0.0304		Procyclic
LbrM07_V2.0950	Unknown function	−1.91	0.0389		Procyclic
LbrM32_V2.2500	Membrane associated protein-like protein	0.79	0.0223	SRR	Metacyclic
LbrM19_V2.1010	Kinesin	0.83	0.0039	SRR	Metacyclic
LbrM29_V2.0470	Unknown function	1.31	0.0383	SRR	Metacyclic
LbrM18_V2.1250	Unknown function	1.55	0.0306	NMT	Metacyclic
LbrM18_V2.0050§	Transporter (sugar)	1.62	0.0169		Metacyclic
LbrM08_V2.0810/LbrM08_V2.0820/LbrM08_V2.0830	Cathepsin L-like protease	1.67	0.0064		Metacyclic
LbrM20_V2.1060	Amastin-like surface protein	2.02	0.0257		Metacyclic
LbrM20_V2.0610	Unknown function	2.05	0.0134	SSR	Metacyclic
LbrM30_V2.2950	Glyceraldehyde 3-phosphate dehydrogenase	2.12	0.0359		Metacyclic
LbrM20_V2.0550	Unknown function	2.46	0.0378	SRR	Metacyclic
LbrM12_V2.0750	Surface antigen proteins (1 and 2)	3.08	0.0000	SRR	Metacyclic
LbrM34_V2.4130	Poly A binding protein	3.47	0.0021	SRR	Metacyclic

**GeneDB ID**, gene accession number on GeneDB (http://www.genedb.org/); **Product**, predicted gene function (annotation from GeneDB); **LogFC**, log_2_ fold change observed between compared RNAs in array studies; **P value**, measure of statistical significance (p<0.05 is statistically significant); **Note**, indicates whether gene is from a specific subset (SSR – amino acid repeat containing gene, NMT – target for *N*-myristoylation); * indicates where array data are validated by qPCR (see Table 4); **Up in**, indicates species or lifecycle stage with increased expression of target gene.

**§:** annotated as a pseudogene in GeneDB.

### Cross species comparison of amastigote RNA expression

**Figure 3:**
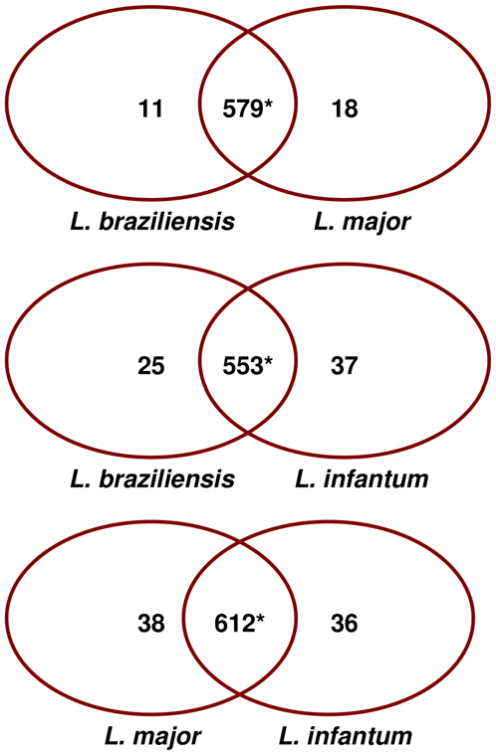
Differential expression of amastigote genes between *Leishmania* species. Venn diagrams showing pairwise comparisons of amastigote RNA expression from the three target *Leishmania* species; the number of genes with increased expression (>1.7-fold, p<0.05) in each species is indicated. *Genes that show no significant difference in expression between species.

Pair-wise comparisons of amastigote RNA profiles for each of the three species identified only a small number of conserved genes showing differential expression. Varying numbers of probes were directly comparable between the species (as some genes are present in two rather than three species) including 608 between *L. braziliensis* and *L. major*, 615 between *L. braziliensis* and *L. infantum* and 686 between *L. major* and *L. infantum*. The expression profiles are shown in Figure 1D–F, with the number of differentially expressed genes in each species summarised in Figure 3 (with details in Table S4). These analyses were used to identify genes showing significantly increased amastigote expression in one species compared with the other two (Table 3). Five genes were identified as showing significantly increased expression in *L. braziliensis* amastigotes: 2 genes with predicted functions (LbrM08_V2.0810 - cathepsin L-like protease, LbrM25_V2.1020 and LbrM35_V2.3370 - ADP-ribosylation factor) and 3 genes of unknown function (LbrM06_V2.0730, LbrM20_V2.0550 and LbrM21_V2.0910). A further 5 genes showed significantly increased expression in *L. major* amastigotes, 4 of which code for proteins of predicted functions (LmjF06.0400 - Fructose biphosphate aldolase, LmjF24.1730 - protein kinase, LmjF31.0830 - Triacyglycerol lipase, LmjF36.5370- tyrosine specific protein phosphatase) while the last codes for an unknown protein (LmjF31.1400). A total of 15 genes showed significantly increased expression in *L. infantum* amastigotes alone, including 10 coding for proteins of unknown function (LinJ05_V3.0670, LinJ07_V3.0270, LinJ07_V3.0950, LinJ10_V3.1230, LinJ13_V3.0770, LinJ14_V3.0470, LinJ18_V3.1160, LinJ24_V3.1530, LinJ29_V3.0110 and LinJ31_V3.1470). and 5 for proteins of predicted function (LinJ12_v4.0671 – surface antigen protein, LinJ04_V3.0320 - mitochondrial exoribonuclease DSS-1, LinJ11_V3.0400 - tubulin-tyrosine ligase-like protein, LinJ31_V3.1490 - surface membrane protein gp46-like protein and the proteophosphoglycan ppg3 array on chromosome 35 (chromosome 34 in *L. braziliensis*). Interestingly, gp46 has also been identified as an upregulated gene in *L. donovani* amastigotes derived from patients with PKDL (post-kala azar dermal leishmaniasis; [52]).

**Table 3:** Conserved genes preferentially expressed in amastigotes.

Genes preferentially expressed in *L. braziliensis* amastigotes					
*L. major* ID	*L. infantum* ID	*L. braziliensis* ID	Product	LogFC (Lb vs. Lm)	LogFC (Lb vs. Li)
LmjF34.0620	LinJ34_V3.0640	LbrM20_V2.0550	Unknown function	2.12	2.33
LmjF08.1020	LinJ08_V3.0960	LbrM08_V2.0810	Cathepsin L-like protease	1.58	1.49
LmjF06.0740	LinJ06_V3.0770	LbrM06_V2.0730	Unknown function	1.00	1.45
LmjF36.3150	LinJ36_V3.3300	LbrM35_V2.3370	Adp-ribosylation factor GTPase activating protein	0.98	0.79
LmjF21.0820	LinJ21_V3.0900	LbrM21_V2.0910	Unknown function	0.92	0.95

Genes preferentially expressed in *L. infantum* amastigotes					
*L. major* ID	*L. infantum* ID	*L. braziliensis* ID	Product	LogFC (Li vs. Lb)	LogFC (Li vs. Lm)
LmjF03.0230	LinJ03_V3.0220	LbrM03_V2.0240	Long chain fatty Acyl CoA synthetase	2.41	2.30
LmjF04.0330	LinJ04_V3.0320	LbrM04_V2.0360	Mitochondrial exoribonuclease DSS-1	1.19	1.99
LmjF05.0670	LinJ05_V3.0670	LbrM05_V2.0660	Unknown function	2.46	2.34
LmjF07.0120	LinJ07_V3.0270	LbrM07_V2.0120	Unknown function	1.74	1.55
LmjF07.0840	LinJ07_V3.0950	LbrM07_V2.0890	Unknown function	2.21	0.80
LmjF10.1140	LinJ10_V3.1230	LbrM10_V2.1250	Unknown function	2.10	2.73
LmjF11.0400	LinJ11_V3.0400	LbrM11_V2.0080	Tubulin-tyrosine ligase-like protein	1.11	1.01
LmjF13.0880	LinJ13_V3.0770	LbrM13_V2.0690	Unknown function	1.82	1.64
LmjF14.0470§	LinJ14_V3.0470	LbrM14_V2.0480	Unknown function	0.85	0.86
LmjF18.1150	LinJ18_V3.1160	LbrM18_V2.1250	Unknown function	1.27	0.77
LmjF22.1680§	LinJ22_V3.1530	LbrM22_V2.1590§	Phosphoinositide-specific phosphatase C	0.98	0.86
LmjF24.1470	LinJ24_V3.1530	LbrM24_V2.1380	Unknown function	2.58	2.70
LmjF29.0110	LinJ29_V3.0110	LbrM29_V2.0120	Unknown function	1.27	0.95
LmjF31.1440	LinJ31_V3.1470	LbrM31_V2.1670	Unknown function	1.30	1.26
LmjF31.1440	LinJ31_V3.1470	LbrM31_V2.1670	Unknown function	2.13	2.52
LmjF35.0520	LinJ35_V3.0530	LbrM34_V2.0520	Proteophosphoglycan ppg3, putative	1.53	1.24

Genes preferentially expressed in *L. major* amastigotes					
*L. major* ID	*L. infantum* ID	*L. braziliensis* ID	Product	LogFC (Lm vs. Lb)	LogFC (Lm vs. Li)
LmjF06.0400§	LinJ06.0400§	LbrM06_V2.0380§	Fructose biphosphate aldolase	1.08	0.81
LmjF24.1730	LinJ24_V3.1800	LbrM24_V2.1790	Protein kinase, putative	1.15	1.15
LmjF31.0830	LinJ31_V3.0860	LbrM31_V2.1010§	Triacyglycerol lipase	0.78	0.79
LmjF31.1400	LinJ31_V3.1430	LbrM31_V2.1560	Unknown function	1.44	0.80
LmjF36.5370	LinJ36_V3.5610	LbrM35_V2.5630	Tyrosine specific protein phosphatase	1.50	0.77

**ID**, gene accession number on GeneDB (http://www.genedb.org/); **Product**, predicted gene function (GeneDB annotation); **LogFC**, log_2_ fold change observed between compared RNAs in array studies. Lb, *L. braziliensis*; Lm, *L. major*; Li, *L. infantum*.

**§:** annotated as pseudogene.

The ppg3 gene is located within an array of related sequences (still undergoing refinement of annotation due to their repetitive structure) on chromosome 35 of *Leishmania* species and codes for one of the glycoprotein constituents of filamentous proteophosphoglycan (fPPG), a major component of promastigote secretory gel (PSG). PSG is produced by parasites within the gut of the sandfly vector, where it contributes to a physical blockage that promotes host blood-feeding and subsequent parasite transmission [53],[54]. The *Leishmania* PPGs belong to a novel class of serine- and threonine-rich proteins possessing large conserved Ala-Pro-Ser repeats that represent major antigenic determinants in all *Leishmania* species examined to date [53],[55],[56]. These repeats are extensively modified by phosphodiester-linked oligosaccharides and terminal manno-oligosaccharides, while the modified PPGs are either water soluble and secreted or membrane-bound. PPGs are expressed in both promastigotes and amastigotes, localising to the flagellar pocket, endosomes, lysosomes and the external surface of the parasite [57],[58],[59]. Surface PPGs are important in parasite-macrophage interactions: they apparently bind to host cells to modulate macrophage function during early infection, both by inhibiting tumor necrosis factor-α production and synergizing with interferon-γ to stimulate the production of nitric oxide [60]. The significance of increased RNA expression from the PPG gene array in *L. infantum* amastigotes (as compared with *L. major* and *L. braziliensis* amastigotes) is unknown; no studies have yet investigated the *L. infantum* PPGs or addressed whether one or more of these molecules might perform a species-specific role in establishing and maintaining amastigotes within host macrophages.

**Figure 4:**
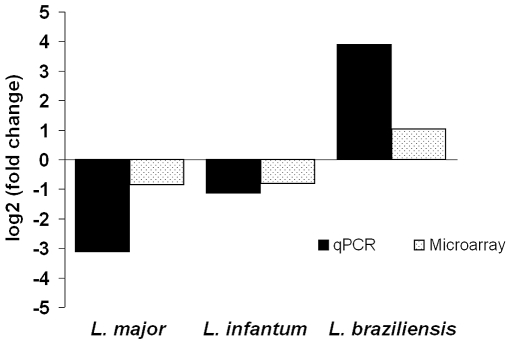
Calmodulin RNA expression is upregulated in amastigotes of *L. braziliensis*. Comparison of amastigote RNA levels by both microarray and RT-qPCR analysis demonstrates increased expression from the chromosome 9 calmodulin gene array in *L. braziliensis* but decreased expression in *L. major* and *L. infantum*. 2 independent biological replicates were used to generate data from each species.

Calmodulin, the ubiquitous eukaryotic intracellular calcium receptor, plays a role in the regulation of many cellular proteins and transmembrane ion transporters with wide-ranging downstream physiological consequences. Calmodulin is encoded by a 3-gene array on chromosome 9 in *Leishmania* but has not yet been widely studied in this organism, despite linkage to the modulation of a plasma membrane Ca^2+^-ATPase in *L. donovani* [61]. In this present study, both the array analyses and subsequent RT-qPCRs show decreased abundance of calmodulin transcripts in amastigotes of *L. major* and *L. infantum* as compared with procyclic and metacyclic parasites. This correlates with the observed changes in calmodulin protein during *in vitro* differentiation of *L. donovani* [62]. By contrast, calmodulin transcript levels are upregulated in *L. braziliensis* amastigotes (Figure 4). To date, it is unclear whether these RNA differences reflect mRNAs derived from one or several of the gene copies, nor how this distribution might vary between the species. It is also unknown whether the varying RNA levels correlate with protein expression in *L. braziliensis* and how this might influence calmodulin function in different amastigote populations.

### Genes differentially distributed between the three representative *Leishmania* species are usually constitutively expressed

The small number of differentially distributed genes identified by comparative genomic analysis of *L. major*, *L. infantum* and *L. braziliensis* [1] include several well-characterised stage specific genes that are implicated in vector transmission and virulence in the host (e.g. A2, HASPB, SHERP [11],[34],[63]. The expression profiles for the ∼200 differentially distributed genes were determined from the *L. braziliensis* life cycle analysis generated here and supplemented with published microarray data generated from life cycle studies of *L. major* and *L. infantum* [12],[14] (Table S1). These cumulative results have confirmed that all genes differentially distributed between the three species are expressed in all life cycle stages. Only 34 of these sequences are regulated between stages, however: 19 in *L. major*, 9 in *L. infantum* and 6 in *L. braziliensis*. The majority of these genes encode proteins of unknown function while the remainder are the HASPA, HASPB and SHERP genes from *L. Leishmania* species as well as an adenine phosphoribosyltransferase (LbrM26_V2.0120) and a unique gene family in *L. braziliensis* (LbrM23_V2.1110 and LbrM23_V2.1120) and the *L. infantum* specific LinJ22_V3.0670.

### *Leishmania* transcriptomes undergo little regulation but the conserved genes that are regulated vary between species

Previous comparisons of the life cycle expression profiles of *L. major*, *L. infantum* and *L. mexicana* have revealed similar numbers of genes that are regulated during the life cycle [12],[13],[14]. The most interesting observation associated with these data is that, while these regulated genes are conserved between all three species, whether they are regulated or not appears to be a species-specific property. As an example of this, only 114/1228 of the conserved regulated genes of *L. major* and *L. infantum* undergo modulation in both species [14]. A similar effect has been observed in comparisons between *L. major* and *L. mexicana* promastigote RNA expression profiles [12]. In this current study, of the 21 genes identified as being stage regulated in *L. braziliensis* (Table S5), 14 are only regulated in this species while 6 share regulation with either *L. major* or *L. infantum* and one, a 3′-nucleotidase/nuclease, is regulated in all three species and shows upregulation in amastigotes [12],[14]. *Leishmania* parasites are purine auxotrophs and require mechanisms to salvage these essential nutrients from the sandfly gut and the host [64],[65],[66]. The 3′-nucleotidase/nuclease [67] degrades and dephosphorylates exogenous purine sources to nucleosides that can be transported across the plasma membrane [67]. Independent studies in *L. donovani* [68] and *L. mexicana* [69] have shown that 3′-nucleotidase/nuclease mRNA transcripts are upregulated in promastigotes when parasites are starved of purines but not in amastigotes. One interpretation of these conflicting observations could be that there are additional gene copies expressing nucleotidase/nuclease activity during the parasite life cycle. The complete list of all regulated genes identified in *L. braziliensis*, *L. major* and *L. infantum* is presented in Table S5.

### Host immune pressure reduces parasite dissemination and survival in the viscera but does not influence parasite RNA expression profiles

The influence of host immune pressure on gene expression in *Leishmania* has not been studied *in vivo* to our knowledge. In this study, parasite burdens, lesion development, and parasite gene expression profiles were examined in immunocompetent BALB/c and immunocompromised Rag2^−/−^γ_c_^−/−^ mice which have a mixed genetic background of 129 Ola, BALB/c and C57BL/6 [33]. These latter mice possess a mutation in the common γ-chain receptor (a component of the receptors for IL-2, -4, -7, -9 and -15) and in the recombination activating gene 2 (Rag2; required for V(J)D rearrangement) and are characterised by a complete absence of mature T lymphocytes, B lymphocytes and NK cells [33].

**Figure 5:**
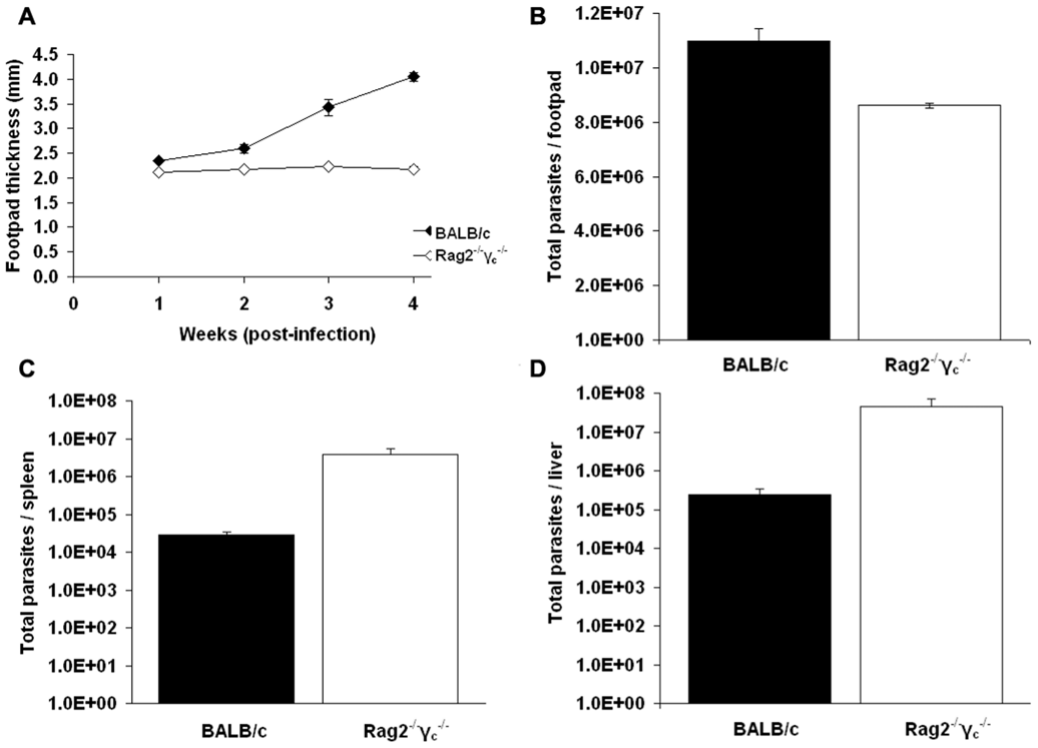
Comparison of *L. major* infections in BALB/c and Rag2^−/−^γ_c_^−/−^ mice. Groups of 5 mice of each strain were inoculated with *L. major* purified metacyclics and infections monitored over a 4 week period as described (Materials and Methods). (A) Lesion development (footpad thickness); (B) parasite burdens in footpad lesions after 4 weeks; (C) parasite burdens in spleen after 4 weeks; (D) parasite burdens in liver after 4 weeks. The data presented are representative of two independent studies; error bars represent the standard error of the mean and the * indicates a significant difference (p<0.05) as determined by Students t-test (unpaired).

Footpad infections were established by inoculation with 4×10^6^ metacyclic *L. major* parasites and resultant lesion formation was measured at weekly intervals. The Rag2^−/−^γ_c_^−/−^ mice displayed no signs of inflammation in the footpad and did not develop cutaneous lesions. In contrast, vigorous lesion development was seen in BALB/c mice with a greater than 2-fold increase in footpad thickness at one month post-infection (Figure 5A). Parasite burdens in the footpads of Rag2^−/−^γ_c_^−/−^ mice were significantly lower than in the BALB/c mice; however these immunocompromised mice showed 2.5-fold higher parasite burdens in the liver and spleen (Figure 5B–D). Enhanced parasite burdens in visceral organs may arise from accelerated dissemination of parasites through the lymphatic system from the site of infection, as Rag2^−/−^γ_c_^−/−^ mice lack peripheral lymph nodes. In addition, survival and proliferation of parasites may be increased in these tissues due to the inability of Rag2^−/−^γ_c_^−/−^ mice to mount protective T and B cell dependant responses required for parasite clearance.

**Figure 6:**
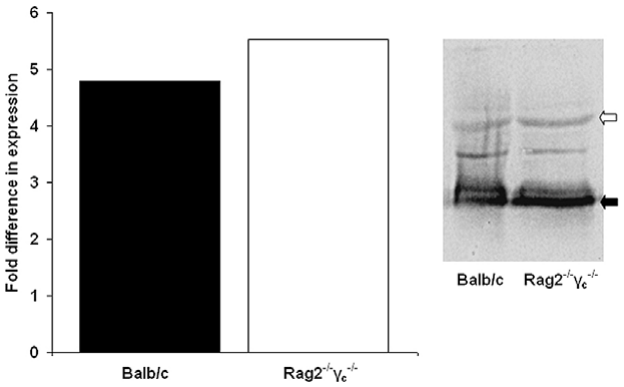
HASPB RNA and protein abundance in *L. major* amastigotes isolated from BALB/c and Rag2^−/−^γ_c_^−/−^ mice. Amastigotes isolated from the livers of two mice of each strain (BALB/c and Rag2^−/−^γ_c_^−/−^ mice), 4 weeks post-infection with *L. major* (see Figure 5), were used for RNA and protein extraction, as described. HASPB expression was analysed by (A) RT-qPCR for RNA and (B) immunoblotting with anti-HASPB for protein (*L. major* HASPB migrates at 38.5 kDa (black arrow); NMT, at 50 kDa (white arrow)). NMT was used as a constitutive control for both RNA and protein expression levels.

Expression profiling of the amastigotes taken from the footpads of these mouse strains revealed only 3 genes that showed a significant difference in expression (out of 700 genes on the array). Only one of these genes (LmjF06.0720, coding for a protein of unknown function) was preferentially expressed in amastigotes from BALB/c mice. The other 2 genes showed increased expression in the Rag2^−/−^γ_c_^−/−^ mice: LmjF24.1840, a lysophospholipase and LmjF35.4730, another “unknown” gene (Table 4). Independent analysis of HASPB transcript and protein abundance in these amastigotes (by RT-qPCR and immunoblotting respectively) revealed no significant differences in expression (Figure 6). This lack of amastigote RNA modulation, assuming that it correlates with protein expression levels, confirms that *Leishmania* parasites are pre-adapted toward intracellular survival, regardless of the state of the host immune system. These data are of relevance to our interpretation of the cross-species amastigote expression results (Table 3) given that the amastigotes were sourced from rodents of different genetic backgrounds.

**Table 4:** Genes differentially expressed in *L. major* amastigotes from different host genetic backgrounds.

GeneDB ID	Product	LogFC	P.Value	Note	Up in
LmjF06.0720	Unknown function	−0.95	0.0091	SRR*	Balb/c amastigotes
LmjF24.1840	Lysophospholipase	1.35	0.0000	*	Rag2^−/−^γ_c_^−/−^ amastigotes
LmjF35.4730§	Unknown function	0.82	0.0003	*	Rag2^−/−^γ_c_^−/−^ amastigotes

**GeneDB ID**, gene accession number on GeneDB (http://www.genedb.org/); **Product**, predicted gene function (annotation from GeneDB); **LogFC**, log_2_ fold change observed between compared RNAs in array studies; **P value**, measure of statistical significance; **Note**, indicates whether gene is from a specific subset (SSR – amino acid repeat containing gene, NMT – target for *N*-myristoylation); *indicates where array data are validated by qPCR (see Table 4); **Up in**, indicates host with increased expression of target gene.

**§:** annotated as a pseudogene in GeneDB.

### Concluding remarks

Small microarrays, targeted towards 9% of the genes conserved between the three representative *Leishmania* species and an additional ∼250 genes differentially distributed between these species, were designed and hybridised with host-derived amastigote RNA isolated from cutaneous lesions (*L. major*), spleens (*L. infantum*) and RAW 264.7 macrophages (*L. braziliensis*). ∼9% of the probed genes were identified as being regulated during the *L. braziliensis* life cycle, a figure comparable to that observed using whole genome arrays for *L. major*, *L. mexicana* and *L. infantum* [12],[14]. It is interesting to note however, that while most regulated genes are conserved between all three representative species, the majority of genes regulated in one species are not regulated in the others. Moreover, comparative expression profiles generated for *L. major*, *L. infantum* and *L. braziliensis* amastigotes revealed that species-specific differential regulation of conserved genes was common and this may impact on parasite survival in the host. These species-specific differences require further study that should focus on determining whether relative protein abundances are affected by increased transcript abundances. Attempts to correlate the relative abundance of mRNA and protein for individual genes in *L. infantum* amastigotes resulted in only modest agreement (∼53% [21]), with these differences attributed to the extensive post-transcriptional, translational and post-translational regulation operating in *Leishmania* parasites [13],[21]. Finally, the lack of modulation of gene expression profiles in *L. major* parasites responding to different immune pressures is consistent with the hypothesis that *Leishmania* parasites are constitutively adapted toward survival in a range of hosts [20].

## Supporting Information

Table S1 List of genes differentially distributed between three *Leishmania* species with microarray analysis derived from this study or ref [14].
(0.06 MB XLS)

Table S2 All 70-mer oligonucleotides used in the microarray studies reported in Depledge et al., 2009.
(0.13 MB XLS)

Table S3 List of primers used in qPCR validation of microarray data.
(0.10 MB PDF)

Table S4 Genes differentially expressed between amastigotes of three *Leishmania* species.
(0.14 MB PDF)

Table S5 *Leishmania* genes demonstrating stage regulation in one or more species.
(0.14 MB PDF)

## References

[1] Murray HW, Berman JD, Davies CR, Saravia NG (2005). Advances in leishmaniasis.. Lancet.

[2] Peacock CS, Seeger K, Harris D, Murphy L, Ruiz JC (2007). Comparative genomic analysis of three Leishmania species that cause diverse human disease.. Nat Genet.

[3] Mauricio IL, Stothard JR, Miles MA (2000). The strange case of Leishmania chagasi.. Parasitol Today.

[4] Guerbouj S, Guizani I, Speybroeck N, Le Ray D, Dujardin JC (2001). Genomic polymorphism of Leishmania infantum: a relationship with clinical pleomorphism?. Infect Genet Evol.

[5] McMahon-Pratt D, Alexander J (2004). Does the Leishmania major paradigm of pathogenesis and protection hold for New World cutaneous leishmaniases or the visceral disease?. Immunol Rev.

[6] Wilson ME, Jeronimo SM, Pearson RD (2005). Immunopathogenesis of infection with the visceralizing Leishmania species.. Microb Pathog.

[7] BenSaid M, Guerbouj S, Saghrouni F, Fathallah-Mili A, Guizani I (2006). Occurrence of Leishmania infantum cutaneous leishmaniasis in central Tunisia.. Trans R Soc Trop Med Hyg.

[8] Lipoldova M, Demant P (2006). Genetic susceptibility to infectious disease: lessons from mouse models of leishmaniasis.. Nat Rev Genet.

[9] Smith DF, Peacock CS, Cruz AK (2007). Comparative genomics: From genotype to disease phenotype in the leishmaniases.. Int J Parasitol.

[10] Ivens AC, Peacock CS, Worthey EA, Murphy L, Aggarwal G (2005). The genome of the kinetoplastid parasite, Leishmania major.. Science.

[11] Zhang WW, Mendez S, Ghosh A, Myler P, Ivens A (2003). Comparison of the A2 gene locus in Leishmania donovani and Leishmania major and its control over cutaneous infection.. J Biol Chem.

[12] Holzer TR, McMaster WR, Forney JD (2006). Expression profiling by whole-genome interspecies microarray hybridization reveals differential gene expression in procyclic promastigotes, lesion-derived amastigotes, and axenic amastigotes in Leishmania mexicana.. Mol Biochem Parasitol.

[13] Leifso K, Cohen-Freue G, Dogra N, Murray A, McMaster WR (2007). Genomic and proteomic expression analysis of Leishmania promastigote and amastigote life stages: the Leishmania genome is constitutively expressed.. Mol Biochem Parasitol.

[14] Rochette A, Raymond F, Ubeda JM, Smith M, Messier N (2008). Genome-wide gene expression profiling analysis of Leishmania major and Leishmania infantum developmental stages reveals substantial differences between the two species.. BMC Genomics.

[15] Saxena A, Lahav T, Holland N, Aggarwal G, Anupama A (2007). Analysis of the Leishmania donovani transcriptome reveals an ordered progression of transient and permanent changes in gene expression during differentiation.. Mol Biochem Parasitol.

[16] El-Sayed NM, Myler PJ, Blandin G, Berriman M, Crabtree J (2005). Comparative genomics of trypanosomatid parasitic protozoa.. Science.

[17] Clayton C, Shapira M (2007). Post-transcriptional regulation of gene expression in trypanosomes and leishmanias.. Mol Biochem Parasitol.

[18] Haile S, Papadopoulou B (2007). Developmental regulation of gene expression in trypanosomatid parasitic protozoa.. Curr Opin Microbiol.

[19] Liang XH, Haritan A, Uliel S, Michaeli S (2003). trans and cis splicing in trypanosomatids: mechanism, factors, and regulation.. Eukaryot Cell.

[20] Cohen-Freue G, Holzer TR, Forney JD, McMaster WR (2007). Global gene expression in Leishmania.. Int J Parasitol.

[21] McNicoll F, Drummelsmith J, Muller M, Madore E, Boilard N (2006). A combined proteomic and transcriptomic approach to the study of stage differentiation in Leishmania infantum.. Proteomics.

[22] Depledge DP, Lower RP, Smith DF (2007). RepSeq–a database of amino acid repeats present in lower eukaryotic pathogens.. BMC Bioinformatics.

[23] Mills E, Price HP, Johner A, Emerson JE, Smith DF (2007). Kinetoplastid PPEF phosphatases: dual acylated proteins expressed in the endomembrane system of Leishmania.. Mol Biochem Parasitol.

[24] Rosenthal PJ (2004). Cysteine proteases of malaria parasites.. Int J Parasitol.

[25] Tetteh KK, Cavanagh DR, Corran P, Musonda R, McBride JS (2005). Extensive antigenic polymorphism within the repeat sequence of the Plasmodium falciparum merozoite surface protein 1 block 2 is incorporated in a minimal polyvalent immunogen.. Infect Immun.

[26] Scherf A, Lopez-Rubio JJ, Riviere L (2008). Antigenic variation in Plasmodium falciparum.. Annu Rev Microbiol.

[27] Alce TM, Gokool S, McGhie D, Stager S, Smith DF (1999). Expression of hydrophilic surface proteins in infective stages of Leishmania donovani.. Mol Biochem Parasitol.

[28] McKean PG, Trenholme KR, Rangarajan D, Keen JK, Smith DF (1997). Diversity in repeat-containing surface proteins of Leishmania major.. Mol Biochem Parasitol.

[29] Nugent PG, Karsani SA, Wait R, Tempero J, Smith DF (2004). Proteomic analysis of Leishmania mexicana differentiation.. Mol Biochem Parasitol.

[30] Rangarajan D, Gokool S, McCrossan MV, Smith DF (1995). The gene B protein localises to the surface of Leishmania major parasites in the absence of metacyclic stage lipophosphoglycan.. J Cell Sci.

[31] Price HP, Menon MR, Panethymitaki C, Goulding D, McKean PG (2003). Myristoyl-CoA:Protein N-Myristoyltransferase, an Essential Enzyme and Potential Drug Target in Kinetoplastid Parasites.. J Biol Chem.

[32] Gelb MH, Van Voorhis WC, Buckner FS, Yokoyama K, Eastman R (2003). Protein farnesyl and N-myristoyl transferases: piggy-back medicinal chemistry targets for the development of antitrypanosomatid and antimalarial therapeutics.. Molecular and Biochemical Parasitology.

[33] Goldman JP, Blundell MP, Lopes L, Kinnon C, Di Santo JP (1998). Enhanced human cell engraftment in mice deficient in RAG2 and the common cytokine receptor gamma chain.. Br J Haematol.

[34] Knuepfer E, Stierhof YD, McKean PG, Smith DF (2001). Characterization of a differentially expressed protein that shows an unusual localization to intracellular membranes in Leishmania major.. Biochem J.

[35] Gamboa D, Van Eys G, Victoir K, Torres K, Adaui V (2007). Putative markers of infective life stages in Leishmania (Viannia) braziliensis.. Parasitology.

[36] Mazurier F, Fontanellas A, Salesse S, Taine L, Landriau S (1999). A novel immunodeficient mouse model–RAG2×common cytokine receptor gamma chain double mutants–requiring exogenous cytokine administration for human hematopoietic stem cell engraftment.. J Interferon Cytokine Res.

[37] Ahmed S, Colmenares M, Soong L, Goldsmith-Pestana K, Munstermann L (2003). Intradermal infection model for pathogenesis and vaccine studies of murine visceral leishmaniasis.. Infect Immun.

[38] Denise H, Poot J, Jimenez M, Ambit A, Herrmann DC (2006). Studies on the CPA cysteine peptidase in the Leishmania infantum genome strain JPCM5.. BMC Mol Biol.

[39] Schonian G, Nasereddin A, Dinse N, Schweynoch C, Schallig HD (2003). PCR diagnosis and characterization of Leishmania in local and imported clinical samples.. Diagn Microbiol Infect Dis.

[40] Flinn HM, Rangarajan D, Smith DF (1994). Expression of a hydrophilic surface protein in infective stages of Leishmania major.. Mol Biochem Parasitol.

[41] Denny PW, Gokool S, Russell DG, Field MC, Smith DF (2000). Acylation-dependent protein export in Leishmania.. J Biol Chem.

[42] Nicolas L, Prina E, Lang T, Milon G (2002). Real-time PCR for detection and quantitation of leishmania in mouse tissues.. J Clin Microbiol.

[43] Nicolas L, Sidjanski S, Colle JH, Milon G (2000). Leishmania major reaches distant cutaneous sites where it persists transiently while persisting durably in the primary dermal site and its draining lymph node: a study with laboratory mice.. Infect Immun.

[44] Brazma A, Hingamp P, Quackenbush J, Sherlock G, Spellman P (2001). Minimum information about a microarray experiment (MIAME)-toward standards for microarray data.. Nat Genet.

[45] Parkinson H, Kapushesky M, Shojatalab M, Abeygunawardena N, Coulson R (2007). ArrayExpress–a public database of microarray experiments and gene expression profiles.. Nucleic Acids Res.

[46] Gadberry MD, Malcomber ST, Doust AN, Kellogg EA (2005). Primaclade–a flexible tool to find conserved PCR primers across multiple species.. Bioinformatics.

[47] Pfaffl MW (2001). A new mathematical model for relative quantification in real-time RT-PCR.. Nucleic Acids Res.

[48] Ouakad M, Bahi-Jaber N, Chenik M, Dellagi K, Louzir H (2007). Selection of endogenous reference genes for gene expression analysis in Leishmania major developmental stages.. Parasitol Res.

[49] Akopyants NS, Matlib RS, Bukanova EN, Smeds MR, Brownstein BH (2004). Expression profiling using random genomic DNA microarrays identifies differentially expressed genes associated with three major developmental stages of the protozoan parasite Leishmania major.. Mol Biochem Parasitol.

[50] Almeida R, Gilmartin BJ, McCann SH, Norrish A, Ivens AC (2004). Expression profiling of the Leishmania life cycle: cDNA arrays identify developmentally regulated genes present but not annotated in the genome.. Mol Biochem Parasitol.

[51] Saxena A, Worthey EA, Yan S, Leland A, Stuart KD (2003). Evaluation of differential gene expression in Leishmania major Friedlin procyclics and metacyclics using DNA microarray analysis.. Mol Biochem Parasitol.

[52] Salotra P, Duncan RC, Singh R, Subba Raju BV, Sreenivas G (2006). Upregulation of surface proteins in Leishmania donovani isolated from patients of post kala-azar dermal leishmaniasis.. Microbes Infect.

[53] Rogers ME, Ilg T, Nikolaev AV, Ferguson MA, Bates PA (2004). Transmission of cutaneous leishmaniasis by sand flies is enhanced by regurgitation of fPPG.. Nature.

[54] Bates PA (2008). Leishmania sand fly interaction: progress and challenges.. Curr Opin Microbiol.

[55] Montgomery J, Ilg T, Thompson JK, Kobe B, Handman E (2000). Identification and predicted structure of a leucine-rich repeat motif shared by Leishmania major proteophosphoglycan and Parasite Surface Antigen 2.. Mol Biochem Parasitol.

[56] Samant M, Sahasrabuddhe AA, Singh N, Gupta SK, Sundar S (2007). Proteophosphoglycan is differentially expressed in sodium stibogluconate-sensitive and resistant Indian clinical isolates of Leishmania donovani.. Parasitology.

[57] Ilg T, Craik D, Currie G, Multhaup G, Bacic A (1998). Stage-specific proteophosphoglycan from Leishmania mexicana amastigotes. Structural characterization of novel mono-, di-, and triphosphorylated phosphodiester-linked oligosaccharides.. J Biol Chem.

[58] Ilg T, Montgomery J, Stierhof YD, Handman E (1999). Molecular cloning and characterization of a novel repeat-containing Leishmania major gene, ppg1, that encodes a membrane-associated form of proteophosphoglycan with a putative glycosylphosphatidylinositol anchor.. J Biol Chem.

[59] Foth B, Piani A, Curtis JM, Ilg T, McConville M (2002). Leishmania major proteophosphoglycans exist as membrane-bound and soluble forms and localise to the cell membrane, the flagellar pocket and the lysosome.. Int J Parasitol.

[60] Piani A, Ilg T, Elefanty AG, Curtis J, Handman E (1999). Leishmania major proteophosphoglycan is expressed by amastigotes and has an immunomodulatory effect on macrophage function.. Microbes Infect.

[61] Mazumder S, Mukherjee T, Ghosh J, Ray M, Bhaduri A (1992). Allosteric modulation of Leishmania donovani plasma membrane Ca(2+)-ATPase by endogenous calmodulin.. J Biol Chem.

[62] Rosenzwieg D, Smith D, Oppordoes F, Stern S, Olafson RW, Zilberstein D (2008). Retooling Leishmania metabolism: from sand fly gut to human macrophage.. FASEB J.

[63] McKean PG, Denny PW, Knuepfer E, Keen JK, Smith DF (2001). Phenotypic changes associated with deletion and overexpression of a stage-regulated gene family in Leishmania.. Cell Microbiol.

[64] Hammond DJ, Gutteridge WE (1984). Purine and pyrimidine metabolism in the Trypanosomatidae.. Mol Biochem Parasitol.

[65] Hassan HF, Coombs GH (1986). A comparative study of the purine- and pyrimidine-metabolising enzymes of a range of trypanosomatids.. Comp Biochem Physiol B.

[66] Steiger RF, Steiger E (1977). Cultivation of Leishmania donovani and Leishmania braziliensis in defined media: nutritional requirements.. J Protozool.

[67] Gottlieb M (1989). The surface membrane 3′-nucleotidase/nuclease of trypanosomatid protozoa.. Parasitol Today.

[68] Debrabant A, Gottlieb M, Dwyer DM (1995). Isolation and characterization of the gene encoding the surface membrane 3′-nucleotidase/nuclease of Leishmania donovani.. Mol Biochem Parasitol.

[69] Sopwith WF, Debrabant A, Yamage M, Dwyer DM, Bates PA (2002). Developmentally regulated expression of a cell surface class I nuclease in Leishmania mexicana.. Int J Parasitol.

