# Supplementary material for: Comparative Expression Profiling of Leishmania: Modulation in Gene Expression between Species and in Different Host Genetic Backgrounds
Source: PLoS Negl Trop Dis. 2009 Jul 7;3(7):e476. doi: 10.1371/journal.pntd.0000476 (PMC2701600; doi:10.1371/journal.pntd.0000476)
Supplement: Table S3 — List of primers used in qPCR validation of microarray data. (0.10 MB PDF) [file pntd.0000476.s003.pdf]

Supplementary Table 3: List of primers used in qPCR validation of microarray data

Gene ID	Gene Annotation	Type	Used for validation of	Primer Forward Sequence	Primer Reverse Sequence
LmjF13.0300 / LinJ13_V3.0330 / LbrM13_V2.0200	Alpha tubulin	Control *	*L.infantum* vs. *L.braziliensis*	TCTTCCTGGACCTCGAGC	CAGCGTGGAACACCATAAAG
LmjF30.3430 / LinJ30_V3.3480	Unknown function	Control *	*L.major* vs. *L.infantum*	TGTACCCACACTCGAAGCAG	ACAGCATCGAAACCTGATTTG
LmjF32.1950 / LinJ32_V3.2060	Unknown function	Control *	*L.major* vs. *L.infantum*	AGTGCGCACATCTGAACAAG	CATTCACCTCGCCGTACA
LbrM18_V2.1700	Gamma-glutamyl cysteine synthetase	Control †	*L.braziliensis* lifecycle	CTACGACTCTATCTCCATCTTCATCA	CACACCAGCCTTCTCCAGC
LinJ29_V3.2410 / LmjF29.2300	Ubiquitin hydrolase	Control¥	*L.major* and *L.infantum* lifecycle	TAGATGAGGTGGCACTGGTGTC	CGTCCATCTCGATCCCCAT
LmjF32.3420	rRNA45	Control§	*L.major* lifecycle	CCTACCATGCCGTGTCCTTCTA	AACGACCCCTGCAGCAATAC
LmjF06.0720	Unknown function	Target	Balb/c vs. $Rag2^{-/-}\gamma_c^{-/-}$	GGACCACCCTGTGTTCTTGT	ACGTAAAGCTGGCTTGCACT
LmjF35.4730	Unknown function	Target	Balb/c vs. $Rag2^{-/-}\gamma_c^{-/-}$	CGCAAGAAAACAAAGGGTTC	CTACGCAACTCTGTCGGTGA
LmjF23.1060 / LinJ23_V3.1220	HASPB	Target	Balb/c vs. $Rag2^{-/-}\gamma_c^{-/-}$	GACGGGGATGTGCAGAAG	CCTTCGGCTGATCATCGT
LinJ01_V3.0490 / LbrM03_V2.0240	Fatty acyl CoA synthetase 1	Target	*L.infantum* vs. *L.braziliensis*	CTCTTCAAGGGCTACTACAAGCA	GTGCCGTAGATGCTCTCCA
LinJ16_V30930 / LbrM16_V2.0930	Flagellar calcium-binding protein	Target	*L.infantum* vs. *L.braziliensis*	GATGCTGTGCTACATATACGACTA	CACCAGCATGTTGCCGGA
LmjF03.0230	long chain fatty Acyl CoA synthetase	Target	*L.major* vs. *L.infantum* Balb/c vs. $Rag2^{-/-}\gamma_c^{-/-}$	CTCCAGAAGCTCAACAAGGTG	CCAGAATCATCGCTGCCT
LmjF13.0880	Unknown function	Target	*L.major* vs. *L.infantum*	CGGTCCTTTTACCTCTTCGACTA	GCTGGAGAAGGAAGTTTTGATC
LmjF29.1480	Unknown function	Target	*L.major* vs. *L.infantum*	ATCAGCAGCAAGACCATCCTAT	GCACCTCCTCCTTCTCTGG
LmjF31.3000	Unknown function	Target	*L.major* vs. *L.infantum*	CGGTACAACTTCCACGTCG	TCTACTGCCATGCTGAGTGC
LmjF32.1950	Unknown function	Target	*L.major* vs. *L.infantum*		
LmjF36.4310	Unknown function	Target	*L.major* vs. *L.infantum*	TACTGCTCAGGCTATCATGGC	ATGGTTTAAGATGTCGAGGCC
LbrM04_V2.0370	ADP-ribosylation factor	Target	*L.braziliensis* lifecycle	GAACAACGCCGGTAAGACAT	AGTCCCAACAGGTGAAGGTG

LbrM07_V2.0360	ATP-dependent DEAD/H RNA helicase	Target	*L.braziliensis* lifecycle	CTACGTGCTAGACCGCTTCC	TAGTTCACCACCACGTCCAA
LbrM09_V2.0960	Calmodulin	Target	*L.braziliensis* lifecycle	GCAGATCTCCGAGTTCAAGG	CCACCTCGTTGATCATGTCC
LbrM11_V2.0560	40S ribosomal protein S21	Target	*L.braziliensis* lifecycle	CTGTGCATTGCTGGCTACCT	GGGCTTCTTCGACTTCGACT
LbrM12_V2.0750	Surface antigen protein	Target	*L.braziliensis* lifecycle	GGCTACCTTCCAGCTGAGTG	TTTAAAATGGCCCAGCTGAC
LbrM21_V2.0360	Unknown function	Target	*L.braziliensis* lifecycle	ACGTTAAACGCGCAGAAGAT	GTGTGTCGCAAGAGTCGAGA
LbrM26_V2.0120	Adenine phosphor-ribosyltransferase	Target	*L.braziliensis* lifecycle	AGCTCTCTATCGACGGCTTG	TGGAGACAGGAAGGCGTATC
LbrM31_V2.2570	3'-nucleotidase/nuclease	Target	*L.braziliensis* lifecycle	GGACTATCTGCGGCTATGGA	AATATTCGGCTACCGCCTCT
LbrM31_V2.3150	ADP-ribosylation factor	Target	*L.braziliensis* lifecycle	CATCTTCGTGGTCGATAGCA	CCTGCTTGTTCGCAAATACA
LbrM34_V2.4130	Poly(a) binding protein	Target	*L.braziliensis* lifecycle	GAGATTGCCAAGGCTGAGTC	CAGGTTCGGGTACTTGCTGT
LbrM32_V2.2500	Membrane associated protein	Target	*L.braziliensis* lifecycle	TCGACGGAGTCTTCTCACCT	GATGCATGCGATCATTTGTC
LbrM20_V2.0550	Unknown function	Target	*L.braziliensis* lifecycle	GTGGTGCTGAATGGACACC	CAGATGCGCCATACCTACCT
LinJ08_V3.0960 / LbrM08_V2.0810	Cathepsin L-like protease	Target	*L.braziliensis* lifecycle *L.infantum* vs. *L.braziliensis*	GCTCGTTGGGTACAACATGAC	CCCCACGAGTTCTTGATCA
LbrM09_V2.0840	Unknown function	Target	*L.braziliensis* lifecycle	ACCAACGCTCCTCAGAAAGA	TTGTGTTGGGGTCTCGTGTA
LbrM18_V2.0050	Major facilitator superfam protein (MFS)	Target	*L.braziliensis* lifecycle	CCGTTTTTCACCAGGACTGT	GGGCGTTCAAGCTACAAGAG
LmjF04.0210	Surface antigen protein	Target	Balb/c vs. $Rag2^{-/-}\gamma_c^{-/-}$	AAGTGCAAGCCCTACGATCT	TCCTATCGCAGAGGTACGTG
LmjF24.0220	Unknown function	Target	Balb/c vs. $Rag2^{-/-}\gamma_c^{-/-}$	CCGTCATCGAAGACAGTGAT	CCGTGTTCTCTAGCAGGTGA
LmjF24.1840	Lysophospholipase	Target	Balb/c vs. $Rag2^{-/-}\gamma_c^{-/-}$	GCGACTATGTGCGTAGCATT	GGGATGCCATACTTCTTCGT
LmjF25.1820	Unknown function	Target	Balb/c vs. $Rag2^{-/-}\gamma_c^{-/-}$	CCATTTCAGACTCGGAGGTT	TCCTCATCGAAGTCATCTGC
LmjF27.0745	Unknown function	Target	Balb/c vs. $Rag2^{-/-}\gamma_c^{-/-}$	GGCTACACACACGCATTGAT	GCGCGTGACACACTTTAGG
LmjF29.1000	Unknown function	Target	Balb/c vs. $Rag2^{-/-}\gamma_c^{-/-}$	GTGGGGACGGCTTTAGTAGG	TACAAGTGCGTTGGCGATAC

LmjF31.3035	Mu-adaptin 4	Target	Balb/c vs. Rag2$^{-/-}\gamma_c^{-/-}$	TGGACTTTGGTGTTGTGGAG	AGATCTCGTTCCCCTCGAAT
LmjF32.2220	Unknown function I	Target	Balb/c vs. Rag2$^{-/-}\gamma_c^{-/-}$	TTGGTGGATGTTTCACTCGT	TGTCTGAACGGTAGCTCTGG
LmjF17.0080	Elongation factor 1-alpha	Target	Balb/c vs. Rag2$^{-/-}\gamma_c^{-/-}$	TCTCCTTCGAAGTGTGGGAC	TAGATGACGGCGTCTGTGTC
LmjF22.0850	3'a2rel-related protein	Target	Balb/c vs. Rag2$^{-/-}\gamma_c^{-/-}$	GGTGTCATCGTTGAGGAGGT	CGACCAAGACGACAACAATG

[*]Normalisation between species performed by randomly selecting genes with similar expression levels between species (i.e. fold change <0.1)

[Ŧ] Normaliser for *L.braziliensis* lifecycle (Gamboa et al., 2007), ref 35

[¥] Normaliser for *L.major* and *L.infantum* lifecycles (Rochette et al., 2008), ref 14

[§] Normaliser for *L.major* lifecycle (Ouakad et al., 2007), ref 48
