# Supplementary material for: Comparative Expression Profiling of Leishmania: Modulation in Gene Expression between Species and in Different Host Genetic Backgrounds
Source: PLoS Negl Trop Dis. 2009 Jul 7;3(7):e476. doi: 10.1371/journal.pntd.0000476 (PMC2701600; doi:10.1371/journal.pntd.0000476)
Supplement: Table S4 — Genes differentially expressed between amastigotes of three Leishmania species. (0.14 MB PDF) [file pntd.0000476.s004.pdf]

# Supplementary Table 4: Genes differentially expressed between amastigotes of three *Leishmania* species

## *L. braziliensis* vs. *L. major*

| *L.braz* GeneDB ID | *L.maj* GeneDB ID | Product | LogFC | P.Value | Preferential expression | Note |
|---|---|---|---|---|---|---|
| LbrM04_V2.0370 | LmjF04.0340 | ADP-ribosylation factor | -1.67 | 0.0178 | *L. braziliensis* | NMT |
| LbrM06_V2.0380§ | LmjF06.0400§ | Fructose biphosphate aldolase | 1.08 | 0.0011 | *L. major* | |
| LbrM07_V2.0880§ | LmjF07.0800 | Flavoprotein subunit protein | 0.78 | 0.0022 | *L. major* | |
| LbrM08_V2.0810 / LbrM08_V2.0820 / LbrM08_V2.0830 | LmjF08.1020 | Cathepsin L-like protease | -1.58 | 0.0001 | *L. braziliensis* | |
| LbrM10_V2.0990 | not present in geneDB | Phosphate repressible phosphate permease | 1.58 | 0.0001 | *L. major* | |
| LbrM24_V2.1790 | LmjF24.1730 | Protein kinase | 1.15 | 0.0016 | *L. major* | NMT |
| LbrM25_V2.1020 | not present in geneDB | Rnase III gene | -1.10 | 0.0000 | *L. braziliensis* | |
| LbrM30_V2.0190§ | LmjF30.0190§ | Polynucleotide kinase 3'-phosphatase | -0.90 | 0.0014 | *L. braziliensis* | |
| LbrM30_V2.0980 | LmjF30.0850 / LmjF30.0860 | Surface protein amastin | 0.81 | 0.0017 | *L. major* | |
| LbrM31_V2.1010§ | LmjF31.0830 | Triacyglycerol lipase | 0.78 | 0.0003 | *L. major* | |
| LbrM31_V2.3150 | LmjF31.2790 | ADP-ribosylation factor | 0.90 | 0.0118 | *L. major* | NMT |
| LbrM31_V2.3260 | LmjF31.2900 | Nucleoporin (NUP54/57) | 1.34 | 0.0170 | *L. major* | SRR |
| LbrM35_V2.3370 | LmjF36.3150 | ADP-ribosylation factor GTPase activating protein | -0.98 | 0.0016 | *L. braziliensis* | |
| LbrM35_V2.5630 | LmjF36.5370 | Tyrosine specific protein phosphatase | 1.50 | 0.0001 | *L. major* | |
| LbrM06_V2.0730 | LmjF06.0740 | Unknown function | -1.00 | 0.0050 | *L. braziliensis* | SRR |
| LbrM07_V2.0890 - LbrM07_V2.0910 | LmjF07.0840 | Unknown function | 1.41 | 0.0010 | *L. major* | SRR |
| LbrM16_V2.0940 | LmjF16.0930 | Unknown function | -1.26 | 0.0262 | *L. braziliensis* | NMT |

| | | | | | | |
|---|---|---|---|---|---|---|
| LbrM20_V2.0550 | LmjF34.0620 | Unknown function | -2.12 | 0.0058 | *L. braziliensis* | SRR |
| LbrM20_V2.0610 | LmjF34.0680 | Unknown function | 1.24 | 0.0173 | *L. major* | SSR |
| LbrM21_V2.0910 | LmjF21.0820 | Unknown function | -0.92 | 0.0001 | *L. braziliensis* | SRR |
| LbrM24_V2.1520 | LmjF24.1360 | Unknown function | 1.29 | 0.0002 | *L. major* | NMT |
| LbrM25_V2.1380 / LbrM25_V2.2450 | LmjF25.1820 | Unknown function | 0.95 | 0.0050 | *L. major* | SRR |
| LbrM25_V2.1870 | LmjF25.2280 | Unknown function | 0.90 | 0.0023 | *L. major* | |
| LbrM27_V2.2850[§] | not present in geneDB | Unknown function | 0.98 | 0.0014 | *L. major* | SRR |
| LbrM31_V2.0130 | LmjF31.0130 | Unknown function | 0.99 | 0.0017 | *L. major* | |
| LbrM31_V2.0370[§] | LmjF31.0260 | Unknown function | 1.35 | 0.0105 | *L. major* | |
| LbrM31_V2.1560 | LmjF31.1400 | Unknown function | 1.44 | 0.0072 | *L. major* | NMT |
| LbrM34_V2.5330 | LmjF35.5390 | Unknown function | -0.79 | 0.0021 | *L. braziliensis* | SRR |
| LbrM34_V2.5330 | LmjF35.5390 | Unknown function | -0.91 | 0.0047 | *L. braziliensis* | |

## *L. braziliensis vs L. infantum*

| ***L.braz* GeneDB ID** | ***L.inf* GeneDB ID** | **Product** | **LogFC** | **P.Value** | **Preferentially expression** | **Note** |
|---|---|---|---|---|---|---|
| LbrM04_V2.0360 | LinJ04_V3.0320 | Mitochondrial exoribonuclease DSS-1, | 1.19 | 0.0177 | *L. infantum* | SRR |
| LbrM07_V2.0880[§] | LinJ07_V3.0910 | Flavoprotein subunit protein | 1.03 | 0.0003 | *L. infantum* | |
| LbrM07_V2.1050 | LinJ07_V3.1150 | RNA binding protein-like protein | 1.61 | 0.0317 | *L. infantum* | SRR |
| LbrM08_V2.0810 / LbrM08_V2.0820 / LbrM08_V2.0830 | LinJ08_V3.0960 | Cathepsin L-like protease | -1.49 | 0.0005 | *L. braziliensis* | |
| LbrM10_V2.0990 | LinJ20_V3.0040 | Phosphate repressible phosphate permease | 0.87 | 0.0214 | *L. infantum* | |
| LbrM11_V2.0080 | LinJ11_V3.0400 | Tubulin-tyrsoine ligase-like protein | 1.11 | 0.0044 | *L. infantum* | SRR |
| LbrM12_V2.0750 | LinJ12_v4.0671 | Surface antigen proteins (1 and 2), | 1.67 | 0.0000 | *L. infantum* | SRR |
| LbrM13_V2.1330 | not present in | Amastin | -1.07 | 0.0419 | *L. braziliensis* | |

| | | | | | | |
|---|---|---|---|---|---|---|
| | geneDB | | | | | |
| LbrM22_V2.1590[§] | LinJ22_V3.1530 | Phosphoinositide-specific phosphatase C | 0.98 | 0.0020 | *L. infantum* | |
| LbrM23_V2.0390 | not present in geneDB | RNase III domain gene | 1.11 | 0.0001 | *L. infantum* | |
| LbrM25_V2.1020 | not present in geneDB | Rnase III gene | -1.26 | 0.0000 | *L. braziliensis* | |
| LbrM30_V2.2930[§] | LinJ30_V3.2970 | Phospholipase c-like protein | -1.12 | 0.0297 | *L. braziliensis* | |
| LbrM31_V2.1680 | LinJ31_V3.1490 | Surface membrane protein gp46-like protein | 1.71 | 0.0479 | *L. infantum* | SRR |
| LbrM31_V2.3150 | LinJ31_V3.2890 | ADP-ribosylation factor, | 0.79 | 0.0357 | *L. infantum* | NMT |
| LbrM31_V2.3260 | LinJ31_V3.3000 | Nucleoporin (NUP54/57), | 1.46 | 0.0144 | *L. infantum* | SRR |
| LbrM33_V2.2660 | LinJ33_V3.2510 | Golgi reassembly stacking protein (GRASP homologue), | 0.83 | 0.0070 | *L. infantum* | NMT |
| LbrM34_V2.0520 / LbrM34_V2.0520 | LinJ35_V3.0510 | Proteophosphoglycan ppg3, | 0.81 | 0.0005 | *L. infantum* | SSR |
| LbrM34_V2.0520 / LbrM34_V2.0520 | LinJ35_V3.0530 | Proteophosphoglycan ppg3, | 1.53 | 0.0000 | *L. infantum* | SSR |
| LbrM34_V2.1870[§] | LinJ35_V3.1950 | Ribosomal protein L32 like protein | -0.81 | 0.0049 | *L. braziliensis* | |
| LbrM35_V2.1760 | LinJ36_V3.1680 | Universal minicircle sequence binding protein 1 | 0.86 | 0.0006 | *L. infantum* | SRR |
| LbrM35_V2.3370 | LinJ36_V3.3300 | ADP-ribosylation factor GTPase activating protein, | -0.79 | 0.0130 | *L. braziliensis* | |
| LbrM05_V2.0660 | LinJ05_V3.0670 | Unknown function | 2.46 | 0.0000 | *L. infantum* | |
| LbrM05_V2.0690 | LinJ05_V3.0700 | Unknown function | 0.97 | 0.0002 | *L. infantum* | SRR |
| LbrM06_V2.0640 | LinJ06_V3.0660 | Unknown function | -1.44 | 0.0493 | *L. braziliensis* | SRR |
| LbrM06_V2.0710 | LinJ06_V3.0750 | Unknown function | 1.12 | 0.0194 | *L. infantum* | SRR |
| LbrM06_V2.0730 | LinJ06_V3.0770 | Unknown function | -1.45 | 0.0003 | *L. braziliensis* | SRR |
| LbrM07_V2.0120 | LinJ07_V3.0270 | Unknown function | 1.74 | 0.0358 | *L. infantum* | |
| LbrM07_V2.0550 | LinJ07_V3.0540 | Unknown function | -0.87 | 0.0060 | *L. braziliensis* | |
| LbrM07_V2.0890 - LbrM07_V2.0910 | LinJ07_V3.0950 - LinJ07_V3.0990 | Unknown function | 0.93 | 0.0109 | *L. infantum* | SRR |

| | | | | | | |
|---|---|---|---|---|---|---|
| LbrM07_V2.0890 - LbrM07_V2.0910 | LinJ07_V3.0950 - LinJ07_V3.0990 | Unknown function | 2.21 | 0.0000 | *L. infantum* | SRR |
| LbrM07_V2.0930 | LinJ07_V3.1010 | Unknown function | 0.98 | 0.0001 | *L. infantum* | SRR |
| LbrM10_V2.1190[§] | LinJ10_V3.1170 | Unknown function | -0.78 | 0.0470 | *L. braziliensis* | |
| LbrM10_V2.1250 | LinJ10_V3.1230 | Unknown function | 2.10 | 0.0209 | *L. infantum* | NMT |
| LbrM11_V2.0160 | LinJ11_V3.0480 | Unknown function | 0.80 | 0.0016 | *L. infantum* | |
| LbrM13_V2.0690 | LinJ13_V3.0770 | Unknown function | 1.82 | 0.0000 | *L. infantum* | SRR |
| LbrM14_V2.0360 | LinJ14_V3.0360 | Unknown function | 1.14 | 0.0001 | *L. infantum* | |
| LbrM14_V2.0480 | LinJ14_V3.0470 | Unknown function | 0.85 | 0.0053 | *L. infantum* | |
| LbrM16_V2.1730 | not present in geneDB | Unknown function | 1.42 | 0.0270 | *L. infantum* | SRR |
| LbrM17_V2.0980 | LinJ17_V3.0990 | Unknown function | -0.81 | 0.0094 | *L. braziliensis* | |
| LbrM18_V2.1250 | LinJ18_V3.1160 | Unknown function | 1.27 | 0.0093 | *L. infantum* | NMT |
| LbrM20_V2.0550 | LinJ34_V3.0640 | Unknown function | -2.33 | 0.0045 | *L. braziliensis* | SRR |
| LbrM21_V2.0910 | LinJ21_V3.0900 | Unknown function | -0.95 | 0.0002 | *L. braziliensis* | SRR |
| LbrM21_V2.1080 | LinJ21_V3.1070 | Unknown function | -0.81 | 0.0033 | *L. braziliensis* | |
| LbrM22_V2.1410[§] | LinJ22.1330 | Unknown function | -1.04 | 0.0000 | *L. braziliensis* | |
| LbrM24_V2.1380 | LinJ24_V3.1530 | Unknown function | 2.58 | 0.0052 | *L. infantum* | |
| LbrM24_V2.1520 | LinJ24_V3.1400 | Unknown function | 0.89 | 0.0101 | *L. infantum* | NMT |
| LbrM25_V2.1390 | LinJ25_V3.1910 | Unknown function | -1.03 | 0.0003 | *L. braziliensis* | SRR |
| LbrM25_V2.1620 | LinJ25_V3.2140 | Unknown function | -0.97 | 0.0019 | *L. braziliensis* | NMT |
| LbrM27_V2.2850[§] | not present in geneDB | Unknown function | 1.17 | 0.0004 | *L. infantum* | SRR |
| LbrM29_V2.0120 | LinJ29_V3.0110 | Unknown function | 1.27 | 0.0001 | *L. infantum* | SRR |
| LbrM29_V2.0820 | LinJ29_V3.0830 | Unknown function | -0.82 | 0.0001 | *L. braziliensis* | SRR |
| LbrM29_V2.2100 | not present in geneDB | Unknown function | -0.80 | 0.0228 | *L. braziliensis* | |
| LbrM31_V2.0130 | LinJ31_V3.0140 | Unknown function | 0.86 | 0.0089 | *L. infantum* | |

| | | | | | | |
|---|---|---|---|---|---|---|
| LbrM31_V2.0370§ | LinJ31_V3.0280 | Unknown function | 1.14 | 0.0386 | *L. infantum* | |
| LbrM31_V2.1240§ | LinJ31_V3.1040 | Unknown function | -1.08 | 0.0013 | *L. braziliensis* | |
| LbrM31_V2.1670 | LinJ31_V3.1470 | Unknown function | 2.13 | 0.0063 | *L. infantum* | SRR |
| LbrM31_V2.2330 | LinJ31_V3.2110 | Unknown function | -1.38 | 0.0139 | *L. braziliensis* | SRR |
| LbrM32_V2.3800 | not present in geneDB | Unknown function | -0.87 | 0.0018 | *L. braziliensis* | |
| LbrM33_V2.3200 | LinJ33_V3.3070 | Unknown function | -1.32 | 0.0001 | *L. braziliensis* | SRR |
| LbrM35_V2.1250 | LinJ36_V3.1180 | Unknown function | -0.79 | 0.0279 | *L. braziliensis* | |

## *L. infantum vs L. major*

| ***L.inf* GeneDB ID** | ***L.maj* GeneDB ID** | **Product** | **LogFC** | **P.Value** | **Preferentially expressed** | **Note** |
|---|---|---|---|---|---|---|
| LinJ03_V3.0220 | LmjF03.0230 | Long chain fatty Acyl CoA synthetase, | 2.30 | 0.0000 | *L. major* | NMT |
| LinJ03_V3.0570 | LmjF03.0590 | DNA repair helicase | -1.77 | 0.0019 | *L. infantum* | |
| LinJ03_V3.0720 | LmjF03.0740 | Cytochrome c oxidase copper chaperone, | -0.94 | 0.0092 | *L. infantum* | NMT |
| LinJ04_V3.0320 | LmjF04.0330 | Mitochondrial exoribonuclease DSS-1, | 1.99 | 0.0000 | *L. major* | SRR |
| LinJ04_V3.1180 | LmjF04.1165§ | 31-0-demethyl-FK506 methyltransferase | -1.45 | 0.0133 | *L. infantum* | |
| LinJ05_V3.0480 | LmjF05.0480 | Monocarboxylate transporter-like protein | 0.78 | 0.0444 | *L. major* | |
| LinJ06_V3.0400§ | LmjF06.0400§ | Fructose biphosphate aldolase | -0.81 | 0.0010 | *L. infantum* | |
| LinJ10_V3.0490 - LinJ10_V3.0530 | LmjF10.0460 / LmjF10.0465 / LmjF10.0470 / LmjF10.0480 | GP63, leishmanolysin | -1.47 | 0.0159 | *L. infantum* | SRR |
| LinJ11_V3.0100 | LmjF11.0100 | Seryl-tRNA synthetase, | -1.68 | 0.0039 | *L. infantum* | |
| LinJ11_V3.0400 | LmjF11.0400 | Tubulin-tyrsoine ligase-like protein | 1.01 | 0.0003 | *L. major* | SRR |
| LinJ12_v4.0663 | LmjF12.0755 | Surface antigen proteins (1 and 2), | 1.02 | 0.0000 | *L. major* | SRR |
| LinJ12_v4.0666 | LmjF12.1070 | Surface antigen protein 2 | 0.80 | 0.0002 | *L. major* | SRR |
| LinJ12_v4.0668 | LmjF12.0780 | Surface antigen protein 2 precursor | 0.83 | 0.0000 | *L. major* | |

| | | | | | | |
|---|---|---|---|---|---|---|
| LinJ12_v4.0671 | LmjF12.1090 | Surface antigen proteins (1 and 2), | 1.38 | 0.0000 | *L. major* | SRR |
| LinJ14_V3.1180 | LmjF14.1100 | Kinesin K39, | -0.95 | 0.0434 | *L. infantum* | SRR |
| LinJ16_V30920 | LmjF16.0910[§] | Flagellar calcium binding protein | -1.87 | 0.0110 | *L. infantum* | |
| LinJ17_V3.0330 | LmjF17.0280 | Histone H2A, | -1.42 | 0.0118 | *L. infantum* | SRR |
| LinJ20_V3.0970 | LmjF20.0960 | Protein kinase, | 1.86 | 0.0007 | *L. major* | SSR |
| LinJ22_V3.1530 | LmjF22.1680[§] | Phosphoinositide-specific phosphatase C | 0.86 | 0.0002 | *L. major* | |
| LinJ24_V3.0630 | LmjF24.0620 | Ubiquitin hydrolase, | -1.26 | 0.0433 | *L. infantum* | |
| LinJ24_V3.1800 | LmjF24.1730 | Protein kinase, | -1.15 | 0.0001 | *L. infantum* | NMT |
| LinJ27_V3.2490 | LmjF27.0510 | Cysteine peptidase, Clan CA, family C2, | -1.13 | 0.0374 | *L. infantum* | SRR |
| LinJ28_V3.0470 | LmjF29.0280 | D-lactate dehydrogenase | -0.85 | 0.0012 | *L. infantum* | |
| LinJ30_V3.1490 | LmjF30.1410 | Ama1 protein, | 0.79 | 0.0271 | *L. major* | |
| LinJ30_V3.2970 | LmjF30.2950 | Phospholipase c-like protein | -0.88 | 0.0155 | *L. infantum* | |
| LinJ31_V3.0860 | LmjF31.0830 | Triacyglycerol lipase | -0.79 | 0.0000 | *L. infantum* | |
| LinJ31_V3.1490 | LmjF31.1460 | Surface membrane protein gp46-like protein | 1.18 | 0.0353 | *L. major* | SRR |
| LinJ31_V3.2370 | LmjF31.2300 | 3' nucleotidase/nuclease | -0.79 | 0.0000 | *L. infantum* | |
| LinJ32_V3.1900 | LmjF32.1810 | Protein kinase, | -1.52 | 0.0235 | *L. infantum* | NMT |
| LinJ35_V3.0260 | LmjF35.0260 | GTP-ase activating protein, | 0.98 | 0.0451 | *L. major* | |
| LinJ35_V3.0500 | LmjF35.0500 | Proteophosphoglycan ppg3, | 1.09 | 0.0000 | *L. major* | SSR |
| LinJ35_V3.0520 | LmjF35.0520 / LmjF35.0540 | Proteophosphoglycan ppg3, | 0.84 | 0.0000 | *L. major* | SSR |
| LinJ35_V3.0530 | LmjF35.0520 / LmjF35.0540 | Proteophosphoglycan ppg3, | 1.24 | 0.0000 | *L. major* | SSR |
| LinJ35_V3.0540 | LmjF35.0540 | Proteophosphoglycan ppg3, | 1.73 | 0.0068 | *L. major* | |
| LinJ36_V3.5610 | LmjF36.5370 | Tyrosine specific protein phosphatase | -0.77 | 0.0036 | *L. infantum* | |
| LinJ05_V3.0670 | LmjF05.0670 | Unknown function | 2.34 | 0.0000 | *L. major* | |
| LinJ05_V3.0690 | LmjF05.0690 | Unknown function | 1.14 | 0.0183 | *L. major* | SRR |
| LinJ07_V3.0270 | LmjF07.0120 | Unknown function | 1.55 | 0.0085 | *L. major* | |

| | | | | | | |
|---|---|---|---|---|---|---|
| LinJ07_V3.0950 - LinJ07_V3.0990 | LmjF07.0840 | Unknown function | 0.80 | 0.0087 | *L. major* | SRR |
| LinJ09_V3.1640 | LmjF09.1550 | Unknown function | -1.02 | 0.0496 | *L. infantum* | NMT |
| LinJ10_V3.0790 | LmjF10.0740 | Unknown function | 0.77 | 0.0028 | *L. major* | |
| LinJ10_V3.1230 | LmjF10.1140 | Unknown function | 2.73 | 0.0001 | *L. major* | NMT |
| LinJ13_V3.0770 | LmjF13.0880 | Unknown function | 1.64 | 0.0000 | *L. major* | SRR |
| LinJ14_V3.0470 | LmjF14.0470[§] | Unknown function | 0.86 | 0.0001 | *L. major* | |
| LinJ15_V3.0160 | LmjF15.0160 | Unknown function | -1.56 | 0.0169 | *L. infantum* | SRR |
| LinJ16_V3.0940 | LmjF16.0930 | Unknown function | 1.34 | 0.0021 | *L. major* | NMT |
| LinJ18_V3.1160 | LmjF18.1150 | Unknown function | 0.77 | 0.0212 | *L. major* | NMT |
| LinJ19_V3.1270 | LmjF19.1280 | Unknown function | -1.27 | 0.0292 | *L. infantum* | NMT |
| LinJ20_V3.0530 | LmjF20.0460 | Unknown function | -1.88 | 0.0062 | *L. infantum* | |
| LinJ20_V3.0770 | LmjF20.0705 | Unknown function | 1.11 | 0.0000 | *L. major* | NMT |
| LinJ20_V3.1200[§] | LmjF20.1175[§] | Unknown function | 0.80 | 0.0316 | *L. major* | |
| LinJ22_V3.0070 | LmjF22.0190 | Unknown function | -0.83 | 0.0013 | *L. infantum* | |
| LinJ23_V3.0100 | LmjF23.0090 | Unknown function | -0.92 | 0.0447 | *L. infantum* | SRR |
| LinJ24_V3.0210 | LmjF24.0220 | Unknown function | -1.68 | 0.0078 | *L. infantum* | |
| LinJ24_V3.1530 | LmjF24.1470 | Unknown function | 2.70 | 0.0001 | *L. major* | |
| LinJ25_V3.1150 | LmjF25.1110 | Unknown function | -2.63 | 0.0000 | *L. infantum* | SRR |
| LinJ26_V3.0390 | LmjF26.0400 | Unknown function | -1.46 | 0.0049 | *L. infantum* | |
| LinJ26_V3.1420 | LmjF26.1440 | Unknown function | -1.97 | 0.0057 | *L. infantum* | NMT |
| LinJ29_V3.0110 | LmjF29.0110 | Unknown function | 0.95 | 0.0000 | *L. major* | SRR |
| LinJ29_V3.0870 | LmjF29.0830 | Unknown function | 1.48 | 0.0013 | *L. major* | SRR |
| LinJ31_V3.1090 | LmjF31.1090 | Unknown function | -1.80 | 0.0005 | *L. infantum* | |
| LinJ31_V3.1430 | LmjF31.1400 | Unknown function | -0.80 | 0.0385 | *L. infantum* | NMT |
| LinJ31_V3.1470 | LmjF31.1440 | Unknown function | 1.05 | 0.0000 | *L. major* | SRR |
| LinJ31_V3.1470 | LmjF31.1440 | Unknown function | 1.26 | 0.0000 | *L. major* | SRR |

| | | | | | | |
|---|---|---|---|---|---|---|
| LinJ31_V3.1470 | LmjF31.1440 | Unknown function | 2.52 | 0.0000 | *L. major* | SRR |
| LinJ31_V3.2550 | not present in geneDB | Unknown function | -0.99 | 0.0067 | *L. infantum* | |
| LinJ31_V3.2750 | LmjF31.2670 | Unknown function | -1.77 | 0.0167 | *L. infantum* | SRR |
| LinJ31_V3.3110 | LmjF31.3000 | Unknown function | -1.35 | 0.0098 | *L. infantum* | |
| LinJ32_V3.0220 | LmjF32.0210 | Unknown function | -1.43 | 0.0263 | *L. infantum* | SRR |
| LinJ33_V3.2120 | LmjF33.2000 | Unknown function | 0.82 | 0.0277 | *L. major* | SRR |
| LinJ33_V3.3070 | LmjF33.2910 | Unknown function | -0.84 | 0.0002 | *L. infantum* | SRR |
| LinJ34_V3.0740 | LmjF34.0705 | Unknown function | -1.08 | 0.0435 | *L. infantum* | |
| LinJ36_V3.4360 | LmjF36.4150 | Unknown function | -2.30 | 0.0002 | *L. infantum* | |
| LinJ36_V3.4520 | LmjF36.4310 | Unknown function | -1.70 | 0.0065 | *L. infantum* | |

[§]annotated as a pseudogene in GeneDB
Note: NMT, N-myristoyl transferase target genes; SRR, sequence repeat region (see Depledge et al 2007, ref 22)
