# Supplementary material for: Comparative Expression Profiling of Leishmania: Modulation in Gene Expression between Species and in Different Host Genetic Backgrounds
Source: PLoS Negl Trop Dis. 2009 Jul 7;3(7):e476. doi: 10.1371/journal.pntd.0000476 (PMC2701600; doi:10.1371/journal.pntd.0000476)
Supplement: Table S5 — Leishmania genes demonstrating stage regulation in one or more species. (0.14 MB PDF) [file pntd.0000476.s005.pdf]

Table S5: *Leishmania* genes demonstrating stage regulation in one or more species

L.major Accession number (GeneDB)	Gene Annotation	Fold increase	*p-value*	Up in	L.infantum Accession number (GeneDB)	Fold increase	*p-value*	Up in	Lbraziliensis Accession number (GeneDB)	Gene Annotation	Log Fold increase	*p-value*	Up in
LmjF31.2300	3'-nucleotidase/nuclease	1.7	1.41E-04	Lm ama	LinJ31_V3.2370	2.0	2.46E-05	Li ama	LbrM31_V2.2570	3'-nucleotidase/nuclease	-0.89	0.0272	Ama
LmjF09.0910	calmodulin c	2.8	1.12E-06	Lm pro	LinJ09_V3.0970	2.0	1.93E-06	Li pro	LbrM09_V2.0960	calmodulin, putative	-1.04	0.0274	Ama
LmjF09.0930	calmodulin c	2.8	1.12E-06	Lm pro	LinJ09_V3.0980	2.0	1.93E-06	Li pro	LbrM09_V2.0970	calmodulin, putative	-1.04	0.0274	Ama
LmjF09.0920	calmodulin c	2.8	1.12E-06	Lm pro					LbrM09_V2.0980	calmodulin, putative	-1.04	0.0274	Ama
LmjF12.1090	surface antigen protein 2 e	2.6	2.53E-04	Lm pro					LbrM12_V2.0750	surface antigen proteins (1 and	-2.31	0.0008	Ama
LmjF07.0340									LbrM07_V2.0360	ATP-dependent DEAD/H RNA	-1.11	0.0405	Ama
LmjF11.0760									LbrM11_V2.0550	40S ribosomal protein S21, put	-2.80	0.0359	Ama
LmjF35.4130									LbrM34_V2.4130	poly(a) binding protein, putative	-3.15	0.0197	Ama
LmjF31.2785	hypothetical	1.9	6.27E-06	Lm ama									
LmjF12.0765	surface antigen protein 2 e	2.0	6.94E-06	Lm pro					LbrM12_V2.0750	surface antigen proteins (1 and	1.20	0.0006	Pro
LmjF30.1170	hypothetical	2.5	1.27E-07	Lm pro					LbrM30_V2.1290	hypothetical	3.98	0.0008	Pro
									LbrM26_V2.0120	Adenine phosphoribosyltransfe	0.84	0.0050	Pro
LmjF11.0475									LbrM11_V2.0160	hypotehtical protein	1.08	0.0163	Pro
LmjF21.0300									LbrM21_V2.0360	hypothetical	2.51	0.0126	Pro
LmjF22.1510									LbrM22_V2.1410	Hypothetical protein	0.86	0.0398	Pro
LmjF27.0660									LbrM31_V2.0450	hypothetical protein	4.25	0.0035	Pro
LmjF27.1245									LbrM27_V2.1350	carboxypeptidase	0.99	0.0172	Pro
LmjF31.2790									LbrM31_V2.3150	adp-ribosylation factor, putative	1.91	0.0073	Pro
LmjF33.0180									LbrM33_V2.0190	Hypothetical protein	1.08	0.0397	Pro
LmjF34.0690									LbrM20_V2.4280	hypothetical protein	1.11	0.0062	Pro
LmjF35.0480									LbrM34_V2.0500	Hypothetical protein	1.84	0.0000	Pro
LmjF04.0340									LbrM04_V2.0370	ADP-ribosylation factor, putativ	2.78	0.0425	Pro
LmjF01.0630	hypothetical	2.5	2.10E-05	Lm ama	LinJ01_V3.0650	1.7	5.28E-05	Li ama					
LmjF02.0170	phosphoglycan beta 1,3 galactosyltransferase c	11.8	2.29E-10	Lm ama	LinJ02_V3.0150	22.0	4.29E-12	Li ama					
LmjF02.0200	phosphoglycan beta 1,3 galactosyltransferase c	11.8	2.29E-10	Lm ama	LinJ02_V3.0170	22.0	4.29E-12	Li ama					
LmjF02.0210	phosphoglycan beta 1,3 galactosyltransferase c	11.8	2.29E-10	Lm ama	LinJ02_V3.0180	22.0	4.29E-12	Li ama					
LmjF03.0500	phosphate-repressible phosphate permease	2.0	1.73E-03	Lm ama	LinJ03_V3.0480	1.7	1.05E-03	Li ama					
LmjF05.1090	double-strand-break repair protein rad21 homolog	1.9	3.76E-03	Lm ama	LinJ05_V3.1090	3.0	2.50E-07	Li ama					
LmjF06.0995	hypothetical	1.8	2.81E-04	Lm ama	LinJ06_V3.1030	2.1	6.81E-05	Li ama					
LmjF08.0640	hypothetical	4.8	4.76E-10	Lm ama	LinJ08_V3.0650	2.0	1.76E-07	Li ama					
LmjF08.0670	amastin e	15.1	6.92E-11	Lm ama	LinJ08_V3.0680	5.8	6.34E-09	Li ama					
LmjF08.0680	amastin e	15.1	6.92E-11	Lm ama	LinJ08_V3.0690	6.8	3.00E-14	Li ama					
LmjF08.0690	amastin e	15.1	6.92E-11	Lm ama	LinJ08_V3.0700	4.3	2.14E-10	Li ama					
LmjF08.0700	amastin e	35.7	3.80E-13	Lm ama	LinJ08_V3.0710	4.3	2.14E-10	Li ama					
LmjF08.0710	amastin e	15.1	6.92E-11	Lm ama	LinJ08_V3.0720	6.8	3.00E-14	Li ama					
LmjF08.0720	amastin e	35.7	3.80E-13	Lm ama	LinJ08_V3.0760	1.9	3.29E-06	Li ama					
LmjF08.0730	amastin e	15.1	6.92E-11	Lm ama	LinJ08_V3.0780	2.1	2.91E-04	Li ama					
LmjF08.0740	amastin e	35.7	3.80E-13	Lm ama	LinJ08_V3.0790	1.7	1.61E-03	Li ama					
LmjF08.0750	amastin e	15.1	6.92E-11	Lm ama	LinJ08_V3.1320	5.8	6.34E-09	Li ama					
LmjF08.0760	amastin e	35.7	3.80E-13	Lm ama	LinJ08_V3.1330	4.3	2.14E-10	Li ama					
LmjF09.0060	RNA-binding protein 5	1.7	9.77E-05	Lm ama	LinJ09_V3.0080	3.4	4.17E-09	Li ama					
LmjF10.1050	hypothetical	1.8	7.27E-04	Lm ama	LinJ10_V3.1130	2.7	5.00E-06	Li ama					
LmjF12.0480	hypothetical	5.1	2.27E-10	Lm ama	LinJ12_V3.0440	1.9	5.23E-04	Li ama					
LmjF12.1250	puromycin-sensitive aminopeptidase	1.8	3.10E-05	Lm ama	LinJ12_V3.0830	2.1	2.87E-05	Li ama					
LmjF13.0200	hypothetical	3.8	8.41E-09	Lm ama	LinJ13_V3.0200	2.7	1.40E-06	Li ama					
LmjF14.1360	myo-inositol-1-phosphate synthase	4.7	1.63E-10	Lm ama	LinJ14_V3.1450	2.4	3.84E-07	Li ama					
LmjF14.1400	phosphoglycan beta 1,3 galactosyltransferase c	11.8	2.29E-10	Lm ama	LinJ14_V3.1500	22.0	4.29E-12	Li ama					
LmjF17.1010	hydrolase, alpha/beta fold family	1.8	6.93E-04	Lm ama	LinJ17_V3.1110	1.7	7.07E-03	Li ama					
LmjF22.1580	exosome complex exonuclease RRP45 homolog	1.8	9.96E-05	Lm ama	LinJ22_V3.1430	3.1	7.24E-10	Li ama					
LmjF23.0460	trypanothione synthetase	1.8	4.01E-04	Lm ama	LinJ23_V3.0500	1.7	7.95E-04	Li ama					
LmjF24.2230	hypothetical	1.7	1.36E-05	Lm ama	LinJ24_V3.2320	2.7	1.68E-07	Li ama					
LmjF26.1460	hypothetical	2.0	2.71E-03	Lm ama	LinJ26_V3.1440	1.8	3.73E-05	Li ama					
LmjF27.0910	hypothetical	1.9	2.42E-05	Lm ama	LinJ27_V3.0770	20.8	9.85E-10	Li ama					
LmjF27.2370	hypothetical	1.8	4.35E-04	Lm ama	LinJ27_V3.2320	2.5	1.72E-08	Li ama					
LmjF30.0770	hypothetical	1.8	4.14E-05	Lm ama	LinJ30_V3.0820	2.0	2.59E-06	Li ama					
LmjF30.0940	hypothetical	2.7	6.96E-06	Lm ama	LinJ30_V3.1000	7.1	1.71E-08	Li ama					
LmjF30.1780	protein kinase	1.7	1.04E-02	Lm ama	LinJ30_V3.1780	2.5	2.23E-06	Li ama					
LmjF30.2330	hypothetical	2.4	2.82E-08	Lm ama	LinJ30_V3.2340	1.7	2.90E-05	Li ama					
LmjF31.0450	amastin e	3.2	2.12E-07	Lm ama	LinJ31_V3.0460	2.3	7.88E-08	Li ama					
LmjF31.1170	hypothetical	2.8	1.62E-07	Lm ama	LinJ31_V3.1190	2.1	2.22E-07	Li ama					
LmjF31.2090	hypothetical	2.0	1.02E-06	Lm ama	LinJ31_V3.2140	1.9	7.69E-07	Li ama					
LmjF32.3400	hypothetical	2.3	2.95E-06	Lm ama	LinJ32_V3.3600	1.9	6.59E-06	Li ama					
LmjF32.3990	GIPL galf transferase d	2.0	1.31E-05	Lm ama	LinJ32_V3.4140	2.8	3.62E-07	Li ama					
LmjF33.1620	hypothetical	4.5	1.68E-05	Lm ama	LinJ33_V3.1720	4.2	2.11E-09	Li ama					
LmjF34.0960	amastin e	3.7	4.01E-05	Lm ama	LinJ34_V3.1010	2.2	6.34E-04	Li ama					
LmjF34.1080	amastin e	2.6	4.73E-05	Lm ama	LinJ34_V3.1150	2.6	2.58E-06	Li ama					

NB: The data for *L. major* and *L. infantum* are derived from whole genome analysis; the *L. braziliensis* data represent analysis of only 10% of the genome as described in this manuscript

LmjF34.1560	amastin e	6.8	3.90E-11	Lm ama	LinJ34_V3.1720	1.9	2.12E-06	Li ama					
LmjF34.1680	amastin e	5.2	1.96E-07	Lm ama	LinJ34_V3.1700	2.8	5.82E-06	Li ama					
LmjF34.1720	amastin e	5.5	8.26E-10	Lm ama	LinJ34_V3.1680	2.9	6.21E-07	Li ama					
LmjF34.1840	amastin e	7.7	1.29E-12	Lm ama	LinJ34_V3.1730	2.0	1.01E-04	Li ama					
LmjF34.1960	amastin e	5.2	1.96E-07	Lm ama	LinJ34_V3.2650	2.2	6.34E-04	Li ama					
LmjF34.1980	amastin e	6.8	3.90E-11	Lm ama	LinJ34_V3.2660	2.2	2.07E-04	Li ama					
LmjF35.5160	hypothetical	1.8	1.21E-05	Lm ama	LinJ35_V3.5130	1.7	1.68E-05	Li ama					
LmjF01.0690	hypothetical	1.8	1.10E-03	Lm ama									
LmjF02.0010	phosphoglycan beta 1,3 galactosyltransferase c	2.4	1.50E-07	Lm ama									
LmjF02.0400	hypothetical	1.7	2.89E-02	Lm ama									
LmjF03.0920	hypothetical	1.9	2.96E-06	Lm ama									
LmjF04.1210	protein kinase	2.3	1.84E-07	Lm ama									
LmjF05.0810	hypothetical	2.2	8.66E-07	Lm ama									
LmjF05.1000	hypothetical	2.1	1.87E-06	Lm ama									
LmjF05.1010	methyltransferase	3.0	7.89E-03	Lm ama									
LmjF05.1230	GIPL galf transferase d	2.0	1.31E-05	Lm ama									
LmjF06.0030	hypothetical	2.0	5.94E-06	Lm ama									
LmjF06.1300	hypothetical	2.0	5.13E-07	Lm ama									
LmjF07.0670	hypothetical	2.0	1.09E-05	Lm ama									
LmjF07.1170	phosphoglycan beta 1,3 galactosyltransferase c	2.4	1.50E-07	Lm ama									
LmjF08.0320	mitochondrial associated ribonuclease	2.2	6.02E-07	Lm ama									
LmjF08.0590	hypothetical	2.9	2.48E-06	Lm ama									
LmjF08.0770	amastin e	35.7	3.80E-13	Lm ama									
LmjF08.0800	amastin e	2.4	1.71E-06	Lm ama									
LmjF08.0810	amastin e	4.9	6.24E-10	Lm ama									
LmjF08.0820	amastin e	6.3	7.42E-11	Lm ama									
LmjF08.0840	amastin e	2.4	1.71E-06	Lm ama									
LmjF08.0850	amastin e	4.2	2.29E-10	Lm ama									
LmjF08.1010	cathepsin L-like d	2.6	2.59E-06	Lm ama									
LmjF08.1020	cathepsin L-like d	2.6	2.59E-06	Lm ama									
LmjF08.1030	cathepsin L-like d	2.6	2.59E-06	Lm ama									
LmjF08.1040	cathepsin L-like d	2.6	2.59E-06	Lm ama									
LmjF08.1050	cathepsin L-like d	2.6	2.59E-06	Lm ama									
LmjF08.1060	cathepsin L-like d	2.6	2.59E-06	Lm ama									
LmjF08.1070	cathepsin L-like d	2.6	2.59E-06	Lm ama									
LmjF08.1080	cathepsin L-like d	2.6	2.59E-06	Lm ama									
LmjF08.1225	hypothetical	2.2	7.06E-06	Lm ama									
LmjF08.1270	hypothetical	2.2	7.06E-06	Lm ama									
LmjF09.0010	hypothetical	1.7	5.36E-04	Lm ama									
LmjF09.0320	hypothetical	1.8	6.28E-04	Lm ama									
LmjF09.0330	hypothetical	2.3	2.40E-07	Lm ama									
LmjF10.0020	pteridine transporter d	1.7	7.94444532	Lm ama									
LmjF10.0020	pteridine transporter d	1.7	1.70E-05	Lm ama									
LmjF10.0230	hypothetical	3.7	2.86E-09	Lm ama									
LmjF10.0470	GP63, leishmanolysin	2.0	1.47E-05	Lm ama									
LmjF10.1310	pteridine transporter d	1.7	7.94444532	Lm ama									
LmjF10.1310	pteridine transporter d	1.7	1.70E-05	Lm ama									
LmjF11.0340	hypothetical	1.9	2.04E-05	Lm ama									
LmjF11.1220	ABC transporter d	6.7	3.89E-09	Lm ama									
LmjF11.1250	ABC transporter d	6.7	3.89E-09	Lm ama									
LmjF13.0100	hypothetical	2.1	6.62E-05	Lm ama									
LmjF13.0590	hypothetical	2.3	1.35E-06	Lm ama									
LmjF14.1100	kinesin K39	2.1	1.85E-07	Lm ama									
LmjF14.1320	serine hydroxymethyltransferase	1.8	5.67160194	Lm ama									
LmjF14.1320	serine hydroxymethyltransferase	1.8	4.98E-04	Lm ama									
LmjF15.0140	hypothetical	1.7	2.06E-04	Lm ama									
LmjF15.1040	tryparedoxin d	2.1	3.88E-07	Lm ama									
LmjF15.1080	tryparedoxin d	2.1	3.88E-07	Lm ama									
LmjF15.1120	tryparedoxin d	2.1	3.88E-07	Lm ama									
LmjF15.1160	tryparedoxin d	2.1	3.88E-07	Lm ama									
LmjF16.0090	hypothetical	2.5	7.49E-06	Lm ama									
LmjF16.0230	protein tyrosine phosphatase	2.0	3.32E-03	Lm ama									
LmjF16.0500	hypothetical	2.6	3.88E-07	Lm ama									
LmjF16.1500	hypothetical	2.0	3.86E-03	Lm ama									
LmjF17.0020	hypothetical	5.1	1.19E-07	Lm ama									
LmjF17.0340	hypothetical	4.0	2.32E-09	Lm ama									
LmjF17.0390	protein kinase	2.5	2.11E-07	Lm ama									
LmjF18.0140	hypothetical	1.8	3.56E-06	Lm ama									
LmjF18.0290	hypothetical	1.7	6.06E-06	Lm ama									
LmjF18.1100	hypothetical	1.7	3.14E-05	Lm ama									
LmjF18.1260	hypothetical d	2.3	2.09E-05	Lm ama									
LmjF18.1265	hypothetical d	2.3	2.09E-05	Lm ama									

LmjF18.1270	hypothetical d	2.3	2.09E-05	Lm ama									
LmjF18.1275	hypothetical d	2.3	2.09E-05	Lm ama									
LmjF18.1280	hypothetical d	2.3	2.09E-05	Lm ama									
LmjF18.1285	hypothetical d	2.3	2.09E-05	Lm ama									
LmjF18.1290	hypothetical d	2.3	9.46285023	Lm ama									
LmjF18.1290	hypothetical d	2.3	2.09E-05	Lm ama									
LmjF19.0010	hypothetical	1.8	2.06E-05	Lm ama									
LmjF19.0110	hypothetical	1.7	5.36E-04	Lm ama									
LmjF19.0220	polyprenyl synthase	2.4	7.75E-08	Lm ama									
LmjF19.1347	hypothetical	2.7	5.24E-08	Lm ama									
LmjF19.1420	cysteine protease	3.1	3.06E-08	Lm ama									
LmjF19.1430	hypothetical	1.7	1.04E-02	Lm ama									
LmjF19.1490	hypothetical	1.8	7.45E-06	Lm ama									
LmjF19.1510	hypothetical	1.8	7.45E-06	Lm ama									
LmjF19.1650	GIPL galf transferase d	2.0	1.31E-05	Lm ama									
LmjF20.0450	hypothetical	2.3	2.35E-05	Lm ama									
LmjF20.0705	hypothetical	1.8	2.35E-05	Lm ama									
LmjF20.0960	protein kinase	1.7	2.85E-05	Lm ama									
LmjF20.1590	hypothetical	2.2	2.27E-02	Lm ama									
LmjF20.1660	hypothetical	2.0	1.65E-05	Lm ama									
LmjF21.0010	phosphoglycan beta 1,3 galactosyltransferase c	2.4	1.50E-07	Lm ama									
LmjF21.0040	hypothetical	3.6	1.88E-06	Lm ama									
LmjF21.0560	hypothetical	1.9	3.88E-04	Lm ama									
LmjF21.0730	60S ribosomal protein	1.7	1.08E-02	Lm ama									
LmjF21.1590	hypothetical	1.8	9.29E-04	Lm ama									
LmjF22.0010	hypothetical	2.0	5.13E-07	Lm ama									
LmjF22.0740	hypothetical	3.3	3.64E-08	Lm ama									
LmjF22.1440	hypothetical	1.9	1.13E-05	Lm ama									
LmjF23.0320	hypothetical	1.8	4.79E-03	Lm ama									
LmjF23.0730	RNA-binding protein	3.6	2.86E-09	Lm ama									
LmjF23.1040	HASPA1 d	2.4	1.36E-06	Lm ama									
LmjF23.1082	HASPA2 d	2.4	1.36E-06	Lm ama									
LmjF23.1088	HASPA2 d	2.4	1.36E-06	Lm ama									
LmjF23.1155	hypothetical	1.9	4.22E-05	Lm ama									
LmjF23.1390	hypothetical	1.8	1.92E-05	Lm ama									
LmjF23.1430	DHHC zinc finger domain-like	2.2	2.16E-06	Lm ama									
LmjF24.0470	hypothetical	1.9	1.65E-06	Lm ama									
LmjF24.0580	hypothetical	4.8	1.10E-08	Lm ama									
LmjF24.1270	amastin e	1.9	1.53E-06	Lm ama									
LmjF24.1770	inhibitor of cysteine peptidase	2.7	2.98E-08	Lm ama									
LmjF24.1860	hypothetical	2.4	2.93E-07	Lm ama									
LmjF25.0230	hypothetical	2.0	7.93E-07	Lm ama									
LmjF25.1120	aldehyde dehydrogenase, mitochondrial precursor	3.7	2.82E-08	Lm ama									
LmjF25.2370	adenylate kinase	1.9	2.23E-02	Lm ama									
LmjF25.2460	phosphoglycan beta 1,3 galactosyltransferase c	2.4	1.50E-07	Lm ama									
LmjF26.1510	hypothetical	1.7	1.04E-02	Lm ama									
LmjF26.1520	hypothetical	3.5	1.15E-06	Lm ama									
LmjF26.1800	hypothetical	1.9	3.15E-06	Lm ama									
LmjF27.0200	hypothetical	2.0	1.31E-04	Lm ama									
LmjF27.0260	hypothetical	1.9	8.66E-05	Lm ama									
LmjF27.1150	hypothetical	1.8	7.61E-05	Lm ama									
LmjF27.1210	translation initiation factor eIF2B subunit	1.7	8.32E-05	Lm ama									
LmjF27.1700	hypothetical	2.1	6.83E-05	Lm ama									
LmjF27.1780	casein kinase I	1.8	1.38E-04	Lm ama									
LmjF27.1890	endo/exonuclease Mre11	2.2	1.03E-02	Lm ama									
LmjF27.1930	hypothetical	2.8	4.05E-05	Lm ama									
LmjF27.2140	hypothetical	1.7	1.82E-04	Lm ama									
LmjF27.2350	vesicle-associated membrane protein (VAMP)	1.8	5.91727877	Lm ama									
LmjF27.2350	vesicle-associated membrane protein (VAMP)	1.8	5.82E-04	Lm ama									
LmjF28.0150	hypothetical	1.7	1.71E-05	Lm ama									
LmjF28.0980	hypothetical	2.6	1.57E-08	Lm ama									
LmjF28.1510	hypothetical	1.9	1.14E-02	Lm ama									
LmjF28.2095	hypothetical	1.9	4.24E-04	Lm ama									
LmjF28.2370	serine hydroxymethyltransferase	1.8	5.67160194	Lm ama									
LmjF28.2370	serine hydroxymethyltransferase	1.8	4.98E-04	Lm ama									
LmjF28.2380	subtilisin-like serine peptidase	1.7	1.62E-03	Lm ama									
LmjF28.2660	hypothetical	2.0	3.88E-07	Lm ama									
LmjF28.2910	glutamate dehygrogenase	2.8	12.5090302	Lm ama									
LmjF28.2910	glutamate dehygrogenase	2.8	4.92E-07	Lm ama									
LmjF29.0080	hypothetical	2.0	7.65E-05	Lm ama									
LmjF29.1160	tryparedoxin	1.9	7.84E-06	Lm ama									
LmjF29.1230	hypothetical	1.9	1.20E-05	Lm ama									

LmjF29.2420	hypothetical	1.7	3.57E-04	Lm ama									
LmjF30.0350	kinesin	1.8	2.08E-04	Lm ama									
LmjF30.1130	hypothetical	1.7	2.40E-04	Lm ama									
LmjF30.1260	hypothetical	1.9	1.13E-05	Lm ama									
LmjF30.1390	hypothetical	3.7	1.93E-08	Lm ama									
LmjF30.1410	ama 1 d	2.3	5.13E-07	Lm ama									
LmjF30.1420	ama 1 d	2.3	5.13E-07	Lm ama									
LmjF30.1430	ama 1 d	2.3	5.13E-07	Lm ama									
LmjF30.1460	class i nuclease d	1.8	1.04E-02	Lm ama									
LmjF30.1470	class i nuclease d	1.8	1.04E-02	Lm ama									
LmjF30.1480	class i nuclease d	1.8	1.04E-02	Lm ama									
LmjF30.1490	class i nuclease d	1.8	1.04E-02	Lm ama									
LmjF30.1570	hypothetical	2.2	3.44E-05	Lm ama									
LmjF30.2180	hypothetical	1.9	3.79E-05	Lm ama									
LmjF30.2190	RNA-binding protein	5.8	3.89E-10	Lm ama									
LmjF30.2200	hypothetical	3.7	1.11E-08	Lm ama									
LmjF30.2230	hypothetical	1.8	1.09E-04	Lm ama									
LmjF30.2380	hypothetical	1.7	5.89E-06	Lm ama									
LmjF30.2500	tyrosine/dopa decarboxylase	2.0	1.28E-05	Lm ama									
LmjF30.2950	phospholipase c-like	2.1	7.44E-08	Lm ama									
LmjF30.3230	hypothetical	1.8	1.48E-03	Lm ama									
LmjF30.3620	hypothetical	1.9	2.10E-05	Lm ama									
LmjF31.0020	aquaporin 3	3.0	1.85E-08	Lm ama									
LmjF31.0800	hypothetical	2.3	1.55E-05	Lm ama									
LmjF31.1330	hypothetical	4.1	4.18E-10	Lm ama									
LmjF31.1450	surface membrane protein gp46-like d	2.1	6.63E-07	Lm ama									
LmjF31.1460	surface membrane protein gp46-like d	2.1	6.63E-07	Lm ama									
LmjF31.1510	hypothetical	2.5	6.93E-08	Lm ama									
LmjF31.1790	amino acid permease d	2.2	5.73E-07	Lm ama									
LmjF31.1820	amino acid permease d	2.2	5.73E-07	Lm ama									
LmjF31.1920	hypothetical	2.3	5.32E-07	Lm ama									
LmjF31.2010	hypothetical	2.5	5.32E-07	Lm ama									
LmjF31.2240	hypothetical	2.0	2.86E-06	Lm ama									
LmjF31.2450	hypothetical	2.1	2.07E-06	Lm ama									
LmjF31.2460	lipase	2.6	2.88E-05	Lm ama									
LmjF31.2810	hypothetical	2.1	1.56E-06	Lm ama									
LmjF31.2870	hypothetical	1.9	1.86E-05	Lm ama									
LmjF31.3190	phosphoglycan beta 1,3 galactosyltransferase c	10.9	2.46E-12	Lm ama									
LmjF32.0740	hypothetical	1.8	8.56E-04	Lm ama									
LmjF32.0830	hypothetical	1.9	1.98E-05	Lm ama									
LmjF32.1810	protein kinase	2.3	2.65E-06	Lm ama									
LmjF32.2470 f	hypothetical	1.7	7.03E-04	Lm ama									
LmjF32.2480	hypothetical	1.8	4.26E-06	Lm ama									
LmjF32.3500	hypothetical	1.8	3.47E-05	Lm ama									
LmjF32.3510	hypothetical	1.8	3.47E-05	Lm ama									
LmjF32.3520	hypothetical	1.8	3.47E-05	Lm ama									
LmjF32.3530	hypothetical	1.8	3.47E-05	Lm ama									
LmjF32.3540	hypothetical	1.8	3.47E-05	Lm ama									
LmjF33.0295	autophagocytosis protein b	1.8	6.01E-05	Lm ama									
LmjF33.0700	hypothetical	1.7	8.14E-05	Lm ama									
LmjF33.1310	expression-site associated gene (ESAG3)	1.7	1.56E-03	Lm ama									
LmjF33.1470	RNA-binding protein	2.3	2.71E-06	Lm ama									
LmjF34.0495	splicing factor ptsr1 interacting protein	1.8	1.86E-06	Lm ama									
LmjF34.0500	amastin e	2.1	4.36E-06	Lm ama									
LmjF34.0580	hypothetical	1.7	7.81E-05	Lm ama									
LmjF34.0770	hypothetical	2.2	2.02E-07	Lm ama									
LmjF34.1580	amastin e	5.2	1.96E-07	Lm ama									
LmjF34.1600	amastin e	6.8	3.90E-11	Lm ama									
LmjF34.1620	amastin e	5.2	1.96E-07	Lm ama									
LmjF34.1640	amastin e	2.4	2.65E-03	Lm ama									
LmjF34.1660	amastin e	5.2	1.96E-07	Lm ama									
LmjF34.1700	amastin e	3.7	4.01E-05	Lm ama									
LmjF34.1740	amastin e	6.8	3.90E-11	Lm ama									
LmjF34.1760	amastin e	6.8	3.90E-11	Lm ama									
LmjF34.1780	amastin e	6.8	22.7778882	Lm ama									
LmjF34.1780	amastin e	6.8	3.90E-11	Lm ama									
LmjF34.1800	amastin e	6.8	22.7778882	Lm ama									
LmjF34.1800	amastin e	6.8	3.90E-11	Lm ama									
LmjF34.1820	amastin e	6.8	22.7778882	Lm ama									
LmjF34.1820	amastin e	6.8	3.90E-11	Lm ama									
LmjF34.1840	amastin e	7.7	32.7639615	Lm ama									
LmjF34.1860	amastin e	6.8	22.7778882	Lm ama									

LmjF34.1860	amastin e	6.8	3.90E-11	Lm ama									
LmjF34.1880	amastin e	5.2	22.7778882	Lm ama									
LmjF34.1880	amastin e	5.2	1.96E-07	Lm ama									
LmjF34.1900	amastin e	6.8	3.90E-11	Lm ama									
LmjF34.1920	amastin e	5.7	8.65E-09	Lm ama									
LmjF34.1940	amastin e	6.8	3.90E-11	Lm ama									
LmjF34.2070	hypothetical	1.8	1.44E-05	Lm ama									
LmjF34.2360	hypothetical	1.7	3.21E-03	Lm ama									
LmjF34.3220	aminophospholipid translocase	1.7	1.94E-05	Lm ama									
LmjF34.4300	hypothetical	1.8	7.67E-04	Lm ama									
LmjF35.0010	phosphoglycan beta 1,3 galactosyltransferase c	10.9	2.46E-12	Lm ama									
LmjF35.1050	protein kinase	1.8	3.78E-06	Lm ama									
LmjF35.1480	arginase	2.8	9.64E-09	Lm ama									
LmjF35.1480	arginase	2.8	9.64E-09	Lm ama									
LmjF35.1490	hypothetical	1.9	4.69E-06	Lm ama									
LmjF35.1530	hypothetical	1.8	5.84E-03	Lm ama									
LmjF35.2090	kinesin	1.7	5.65E-05	Lm ama									
LmjF35.2820	hypothetical	1.8	4.25E-04	Lm ama									
LmjF35.3420	hypothetical	1.9	3.26E-06	Lm ama									
LmjF35.3770	hypothetical	2.2	6.76E-04	Lm ama									
LmjF35.4230	hypothetical	10.3	4.56E-02	Lm ama									
LmjF35.4450	hypothetical	2.5	7.11E-08	Lm ama									
LmjF35.4810	hypothetical	1.8	1.28E-05	Lm ama									
LmjF35.5190	NIMA-related kinase	2.9	6.99E-09	Lm ama									
LmjF35.5200	hypothetical	2.2	1.05E-06	Lm ama									
LmjF36.0010	phosphoglycan beta 1,3 galactosyltransferase c	2.4	1.50E-07	Lm ama									
LmjF36.0310	hypothetical	1.8	1.11E-03	Lm ama									
LmjF36.0535	small nuclear ribonucleoprotein	1.8	1.43E-02	Lm ama									
LmjF36.0730	hypothetical	1.8	2.04E-05	Lm ama									
LmjF36.0910	mitogen-activated protein kinase	2.2	5.41E-05	Lm ama									
LmjF36.1270	amastin e	4.8	9.93E-05	Lm ama									
LmjF36.1930	hypothetical	1.7	9.30E-05	Lm ama									
LmjF36.1970	hypothetical	1.8	5.97E-04	Lm ama									
LmjF36.2000	hypothetical	1.8	7.29E-05	Lm ama									
LmjF36.2170	hypothetical	2.0	1.32E-02	Lm ama									
LmjF36.2350	glyceraldehyde 3-phosphate dehydrogenase	3.6	4.49E-07	Lm ama									
LmjF36.2820	hypothetical	1.9	6.26E-05	Lm ama									
LmjF36.3150	ADP-ribosylation factor GTPase activating protein	1.8	2.99E-05	Lm ama									
LmjF36.3320	hypothetical	1.7	1.04E-05	Lm ama									
LmjF36.3540	hypothetical	2.0	1.50E-03	Lm ama									
LmjF36.4250	protein kinase	1.7	1.43E-04	Lm ama									
LmjF36.4640	hypothetical	2.0	2.02E-07	Lm ama									
LmjF36.4870	hypothetical	2.1	6.05E-03	Lm ama									
LmjF36.4880	hypothetical	2.1	6.05E-03	Lm ama									
LmjF36.5520	hypothetical	1.8	1.41E-03	Lm ama									
LmjF36.5690	hypothetical	1.7	1.65E-05	Lm ama									
LmjF36.5960	glycerophosphoryl diester phosphodiesterase	3.1	2.11E-07	Lm ama									
LmjF36.6070	rab-like GTPase activating protein	2.4	6.54E-06	Lm ama									
LmjF36.6080	hypothetical	1.7	1.20E-05	Lm ama									
LmjF36.6150	hypothetical	1.9	1.78E-04	Lm ama									
LmjF36.6345	hypothetical	1.9	2.11E-05	Lm ama									
LmjF36.6440	hypothetical	1.9	7.42E-05	Lm ama									
LmjF36.6550	protein-L-isoaspartate O-methyltransferase	1.8	1.20E-03	Lm ama									
LmjF23.1050	SHERP c	1.7	5.01E-06	Lm pro	LinJ23_V3.1210	2.5	1.89E-06	Li ama					
LmjF23.1080	SHERP c	1.7	5.01E-06	Lm pro	LinJ23_V3.1230	2.5	1.89E-06	Li ama					
LmjF31.0350	amino acid transporter aATP11	4.7	4.29E-06	Lm pro	LinJ31_V3.0370	2.4	1.86E-05	Li ama					
LmjF33.2340	succinyl-coA:3-ketoacid-coenzyme A transferase	2.1	4.05E-06	Lm pro	LinJ33_V3.2470	1.8	1.18E-03	Li ama					
LmjF35.3730	hypothetical	1.7	9.53E-05	Lm pro	LinJ35_V3.3780	1.8	9.53E-06	Li ama					
LmjF01.0620	hypothetical	2.3	4.26E-07	Lm pro	LinJ01_V3.0640	2.4	1.03E-04	Li pro					
LmjF02.0160	phosphoglycan beta 1,3 galactosyltransferase-like	3.8	1.05E-06	Lm pro	LinJ02_V3.0140	1.8	9.97E-04	Li pro					
LmjF02.0550	hypothetical	5.0	2.27E-10	Lm pro	LinJ02_V3.0520	3.9	6.57E-10	Li pro					
LmjF02.0740	peptidyl dipeptidase c	2.5	1.95E-08	Lm pro	LinJ02_V3.0710	1.7	7.74E-04	Li pro					
LmjF05.0280	protein tyrosine phosphatase	2.6	9.65E-07	Lm pro	LinJ05_V3.0280	1.8	1.28E-04	Li pro					
LmjF07.0030	hypothetical	1.8	2.33E-03	Lm pro	LinJ07_V3.0040	2.0	2.47E-04	Li pro					
LmjF07.0310	hypothetical	2.7	3.79E-08	Lm pro	LinJ07_V3.0470	1.8	1.49E-05	Li pro					
LmjF09.1320	PFR component	2.8	1.02E-07	Lm pro	LinJ09_V3.1390	1.9	8.02E-07	Li pro					
LmjF09.1530	hypothetical	1.8	8.34E-05	Lm pro	LinJ09_V3.1620	2.1	2.31E-05	Li pro					
LmjF10.0180	leucine-rich repeat protein	2.3	7.16E-07	Lm pro	LinJ10_V3.0160	2.1	1.23E-05	Li pro					
LmjF10.1230	hypothetical	1.9	2.47E-06	Lm pro	LinJ10_V3.1370	1.9	2.28E-05	Li pro					
LmjF11.0610	hypothetical	2.1	9.84E-07	Lm pro	LinJ11_V3.0620	2.0	3.06E-04	Li pro					
LmjF11.1040	hypothetical	2.0	1.55E-05	Lm pro	LinJ11_V3.1040	2.1	3.27E-05	Li pro					
LmjF12.0400	3'-nucleotidase/nuclease	3.9	9.33E-09	Lm pro	LinJ12_V3.0350	2.0	6.46E-05	Li pro					

LmjF13.0160	protein kinase A regulatory subunit	2.1	6.05E-07	Lm pro	LinJ13_V3.0160	2.5	2.53E-08	Li pro
LmjF13.1160	calmodulin	1.8	1.62E-05	Lm pro	LinJ13_V3.1060	1.8	3.11E-05	Li pro
LmjF13.1190	adenylosuccinate synthetase	2.0	3.69E-05	Lm pro	LinJ13_V3.1090	1.8	1.32E-04	Li pro
LmjF13.1650	dynein heavy chain	2.1	1.42E-07	Lm pro	LinJ13_V3.1390	1.7	8.21E-05	Li pro
LmjF14.0670	fatty acid elongase	3.2	1.75E-09	Lm pro	LinJ14_V3.0700	2.0	9.44E-06	Li pro
LmjF14.0850	calpain-like cysteine peptidase	4.5	3.90E-11	Lm pro	LinJ14_V3.0920	1.7	7.00E-06	Li pro
LmjF14.1160	enolase	2.4	7.56E-08	Lm pro	LinJ14_V3.1240	2.3	3.49E-08	Li pro
LmjF15.1010	glutamate dehydrogenase	1.8	8.28E-06	Lm pro	LinJ15_V3.1070	1.8	4.70E-06	Li pro
LmjF17.0200	receptor-type adenylate cyclase a	1.8	2.35E-05	Lm pro	LinJ17_V3.0120	1.7	2.26E-04	Li pro
LmjF17.0230	receptor-type adenylate cyclase b c	2.6	1.27E-06	Lm pro	LinJ17_V3.0140	1.8	9.38E-04	Li pro
LmjF17.0235	receptor-type adenylate cyclase b c	2.6	1.27E-06	Lm pro	LinJ17_V3.0160	1.8	9.38E-04	Li pro
LmjF17.0800	OSM3-like kinesin	2.0	8.17E-07	Lm pro	LinJ17_V3.0890	1.7	1.81E-04	Li pro
LmjF17.0870	hypothetical	3.6	4.39E-09	Lm pro	LinJ17_V3.0970	2.0	1.77E-06	Li pro
LmjF18.1090	phosphodiesterase	2.3	4.22E-08	Lm pro	LinJ18_V3.1090	1.9	8.06E-05	Li pro
LmjF18.1640	hypothetical	6.6	1.29E-07	Lm pro	LinJ18_V3.1640	3.5	7.19E-10	Li pro
LmjF19.0520	hypothetical	5.9	4.97E-10	Lm pro	LinJ19_V3.0520	4.5	8.18E-12	Li pro
LmjF20.0700	hypothetical	1.8	4.31E-04	Lm pro	LinJ20_V3.0760	1.7	1.48E-04	Li pro
LmjF20.1280	calpain-like cysteine peptidase	2.1	7.22E-07	Lm pro	LinJ20_V3.1320	2.0	8.88E-07	Li pro
LmjF21.0380	hypothetical	2.5	2.17E-07	Lm pro	LinJ21_V3.0440	1.8	3.08E-06	Li pro
LmjF23.0690	3-ketoacyl-CoA thiolase-like	3.3	1.26E-08	Lm pro	LinJ23_V3.0860	2.1	1.97E-04	Li pro
LmjF23.0710	acetyl-CoA synthetase	4.5	8.18E-07	Lm pro	LinJ23_V3.0880	2.0	9.02E-05	Li pro
LmjF23.1020	hypothetical	4.6	8.63E-11	Lm pro	LinJ23_V3.1190	2.0	1.17E-02	Li pro
LmjF23.1300	lathosterol oxidase-like	3.6	8.78E-10	Lm pro	LinJ23_V3.1560	1.9	2.04E-04	Li pro
LmjF24.1030	dynein light chain	2.0	5.26E-06	Lm pro	LinJ24_V3.1050	2.0	1.48E-05	Li pro
LmjF24.1560	hypothetical	4.1	2.21E-09	Lm pro	LinJ24_V3.1630	4.1	3.57E-09	Li pro
LmjF24.1980	hypothetical	2.3	3.14E-07	Lm pro	LinJ24_V3.2060	2.2	2.53E-08	Li pro
LmjF24.2110	hypothetical	1.8	1.20E-04	Lm pro	LinJ24_V3.2200	2.4	7.25E-06	Li pro
LmjF25.1170	ATPase beta subunit c	1.9	2.75E-05	Lm pro	LinJ25_V3.1210	2.1	1.04E-03	Li pro
LmjF25.1180	ATPase beta subunit c	1.9	2.75E-05	Lm pro	LinJ25_V3.2580	2.1	1.04E-03	Li pro
LmjF25.1770	3-oxo-5-alpha-steroid 4-dehydrogenase	3.0	5.74E-09	Lm pro	LinJ25_V3.1850	1.7	1.88E-05	Li pro
LmjF26.1020	dynein heavy chain	1.7	4.61E-05	Lm pro	LinJ26_V3.1000	1.9	5.26E-06	Li pro
LmjF26.2380	hypothetical	3.0	9.24E-08	Lm pro	LinJ26_V3.2400	2.1	2.25E-07	Li pro
LmjF27.0870	hypothetical	1.7	3.05E-05	Lm pro	LinJ27_V3.0720	1.9	1.56E-06	Li pro
LmjF27.2590	dynein heavy chain (pseudogene)	2.0	7.82E-06	Lm pro	LinJ27_V3.2460	2.1	4.13E-05	Li pro
LmjF28.0610	dynein heavy chain	2.0	8.20E-06	Lm pro	LinJ28_V3.0650	1.8	7.66E-04	Li pro
LmjF28.1060	hypothetical	2.9	5.74E-09	Lm pro	LinJ28_V3.1150	2.1	4.54E-05	Li pro
LmjF28.2430	vacuolar ATP synthase subunit B	1.7	2.41E-04	Lm pro	LinJ28_V3.2610	1.8	1.24E-04	Li pro
LmjF29.0350	hypothetical	1.9	3.90E-05	Lm pro	LinJ29_V3.0360	1.7	1.96E-03	Li pro
LmjF29.1000	hypothetical	3.0	2.55E-08	Lm pro	LinJ29_V3.1090	2.4	7.67E-06	Li pro
LmjF29.1100	hypothetical	1.8	1.64E-06	Lm pro	LinJ29_V3.1190	2.0	1.13E-06	Li pro
LmjF29.1170	hypothetical	3.2	9.21E-10	Lm pro	LinJ29_V3.1260	2.5	1.34E-07	Li pro
LmjF29.1750	PFR1D c	4.5	4.39E-09	Lm pro	LinJ29_V3.1880	3.9	4.30E-11	Li pro
LmjF29.1760	PFR1D c	4.5	4.39E-09	Lm pro	LinJ29_V3.1890	3.9	4.30E-11	Li pro
LmjF29.2830	hypothetical	3.7	5.71E-09	Lm pro	LinJ29_V3.2940	2.5	6.20E-08	Li pro
LmjF30.2850	hypothetical	8.5	2.46E-12	Lm pro	LinJ30_V3.2870	4.4	1.41E-09	Li pro
LmjF31.1200	hypothetical	2.0	1.47E-02	Lm pro	LinJ31_V3.1220	2.7	5.84E-06	Li pro
LmjF31.2310	3' nucleotidase/nuclease precursor	4.6	6.92E-11	Lm pro	LinJ31_V3.2380	1.7	6.85E-05	Li pro
LmjF32.0350	hypothetical	2.0	3.18E-06	Lm pro	LinJ32_V3.0360	1.7	2.83E-05	Li pro
LmjF32.0360	hypothetical	2.2	7.18E-07	Lm pro	LinJ32_V3.0370	2.2	3.57E-07	Li pro
LmjF32.0970	calpain-like cysteine peptidase	1.9	2.66E-04	Lm pro	LinJ32_V3.1020	1.8	2.03E-03	Li pro
LmjF32.1760	hypothetical	3.0	9.51E-08	Lm pro	LinJ32_V3.1840	2.2	9.25E-05	Li pro
LmjF32.1910	hypothetical	2.4	6.72E-06	Lm pro	LinJ32_V3.2020	2.8	1.24E-07	Li pro
LmjF32.1940	HSP 70	1.9	4.20E-04	Lm pro	LinJ32_V3.2050	2.0	3.90E-06	Li pro
LmjF32.3010	hypothetical	2.2	1.24E-05	Lm pro	LinJ32_V3.3200	1.7	1.62E-02	Li pro
LmjF33.0610	hypothetical	5.4	2.27E-10	Lm pro	LinJ33_V3.0660	3.7	9.42E-09	Li pro
LmjF34.1520	hypothetical	4.0	9.64E-09	Lm pro	LinJ34_V3.1620	2.0	1.93E-06	Li pro
LmjF34.2760	hypothetical	1.8	3.72E-05	Lm pro	LinJ34_V3.2590	1.7	6.92E-05	Li pro
LmjF34.4600	hypothetical	3.0	4.92E-08	Lm pro	LinJ34_V3.4230	1.9	2.54E-06	Li pro
LmjF35.0980	aldose 1-epimerase	2.4	3.88E-07	Lm pro	LinJ35_V3.1000	1.8	1.86E-04	Li pro
LmjF35.1750	dynein-associated roadblock protein	2.1	1.40E-05	Lm pro	LinJ35_V3.1740	1.8	3.18E-05	Li pro
LmjF35.5340	hypothetical	4.0	1.80E-07	Lm pro	LinJ35_V3.5310	2.4	5.57E-09	Li pro
LmjF36.0740	hypothetical	1.8	4.50E-05	Lm pro	LinJ36_V3.0800	2.4	4.39E-05	Li pro
LmjF36.2850	hypothetical	2.0	7.27E-07	Lm pro	LinJ36_V3.3000	1.8	4.38E-04	Li pro
LmjF36.3620	hypothetical	6.7	2.15E-07	Lm pro	LinJ36_V3.3780	1.8	1.36E-03	Li pro
LmjF36.4170	oxidoreductase	1.7	5.88E-06	Lm pro	LinJ36_V3.4380	1.7	2.34E-05	Li pro
LmjF36.4230	hypothetical	5.7	2.52E-07	Lm pro	LinJ36_V3.4440	4.1	8.58E-07	Li pro
LmjF36.4780	hypothetical	3.2	8.33E-07	Lm pro	LinJ36_V3.5010	2.7	7.83E-10	Li pro
LmjF36.4910	hypothetical	1.9	6.81E-06	Lm pro	LinJ36_V3.5140	2.3	5.17E-07	Li pro
LmjF36.4980	hypothetical	2.5	3.86E-04	Lm pro	LinJ36_V3.5210	1.8	9.02E-04	Li pro
LmjF01.0010	hypothetical	1.8	4.39E-04	Lm pro				
LmjF01.0200	hypothetical	1.7	1.28E-02	Lm pro				
LmjF01.0570	tricarboxylate carrier	1.8	3.72E-03	Lm pro				

LmjF01.0830	peptidyl dipeptidase c	2.5	1.95E-08	Lm pro									
LmjF03.0040	2-aminoethylphosphonate:pyruvateaminotransferase	2.0	5.66E-06	Lm pro									
LmjF03.0140	hypothetical	1.7	2.62E-05	Lm pro									
LmjF03.0200	delta-1-pyrroline-5-carboxylate dehydrogenase	1.9	3.24E-07	Lm pro									
LmjF03.0230	long chain fatty Acyl CoA synthetase	1.9	9.73E-07	Lm pro									
LmjF03.0360	hypothetical	1.9	1.30E-04	Lm pro									
LmjF03.0970	hypothetical	2.3	5.01E-07	Lm pro									
LmjF04.0040	hypothetical	2.5	9.12E-06	Lm pro									
LmjF04.0130	hypothetical c	2.9	1.34E-07	Lm pro									
LmjF04.0140	hypothetical c	2.9	1.34E-07	Lm pro									
LmjF04.0150	hypothetical c	2.9	1.34E-07	Lm pro									
LmjF04.0160	hypothetical c	2.9	1.34E-07	Lm pro									
LmjF04.0170	hypothetical c	2.9	1.34E-07	Lm pro									
LmjF04.0180	surface antigen protein	2.0	9.07E-05	Lm pro									
LmjF04.0190	surface antigen protein	2.7	4.22E-08	Lm pro									
LmjF04.0210	surface antigen protein	1.9	3.15E-06	Lm pro									
LmjF04.0220	hypothetical	1.9	4.54E-04	Lm pro									
LmjF04.0280	adenosine monophosphate deaminase	2.4	1.19E-07	Lm pro									
LmjF04.0310	beta-fructofuranosidase	5.4	2.29E-10	Lm pro									
LmjF04.0320	beta-fructofuranosidase	3.6	1.13E-05	Lm pro									
LmjF04.0625	hypothetical	2.5	2.22E-06	Lm pro									
LmjF04.0690	hypothetical	1.7	9.34E-04	Lm pro									
LmjF04.1020 f	hypothetical	3.8	5.84E-09	Lm pro									
LmjF04.1200	hypothetical	2.0	5.05E-06	Lm pro									
LmjF05.0040	PFR par4	2.8	5.08E-08	Lm pro									
LmjF05.0640	hypothetical	1.7	1.25E-03	Lm pro									
LmjF05.0650	hypothetical	2.0	2.92E-05	Lm pro									
LmjF05.0920	PFR	2.3	3.79E-08	Lm pro									
LmjF05.0960	dipeptidyl-peptidase III	1.9	7.05E-05	Lm pro									
LmjF05.1020	hypothetical	1.7	6.96E-05	Lm pro									
LmjF05.1070	hypothetical	3.0	1.85E-08	Lm pro									
LmjF05.1080	hypothetical	1.7	9.41E-05	Lm pro									
LmjF06.0310	pteridine transporter	3.2	2.34E-06	Lm pro									
LmjF06.0980	hypothetical	1.7	6.65E-05	Lm pro									
LmjF07.0130	tRNA-methyl transferase	2.5	2.19E-03	Lm pro									
LmjF07.0320	hypothetical	2.1	3.64E-06	Lm pro									
LmjF07.0550	hypothetical	1.7	5.54E-06	Lm pro									
LmjF07.0800	flavoprotein	1.8	3.61E-03	Lm pro									
LmjF07.1160	transmembrane amino acid transporter	9.7	2.46E-12	Lm pro									
LmjF08.0960	hypothetical	1.8	6.72E-05	Lm pro									
LmjF08.1120	hypothetical	2.1	1.75E-05	Lm pro									
LmjF09.0150	microtubule associated protein d	2.5	7.52E-06	Lm pro									
LmjF09.0154	microtubule associated protein d	2.5	7.52E-06	Lm pro									
LmjF09.0158	microtubule associated protein d	2.5	7.52E-06	Lm pro									
LmjF09.0162	microtubule associated protein d	2.5	7.52E-06	Lm pro									
LmjF09.0166	microtubule associated protein d	2.5	7.52E-06	Lm pro									
LmjF09.0170	microtubule associated protein d	2.5	7.52E-06	Lm pro									
LmjF09.0174	microtubule associated protein d	2.5	7.52E-06	Lm pro									
LmjF09.0210	hypothetical	2.3	2.02E-07	Lm pro									
LmjF09.0500	hypothetical	2.4	1.56E-06	Lm pro									
LmjF09.0530	leucine-rich repeat protein	2.5	3.49E-07	Lm pro									
LmjF09.0770	oligopeptidase b	2.2	6.54E-08	Lm pro									
LmjF09.1300	hypothetical	2.0	1.95E-06	Lm pro									
LmjF09.1510	hypothetical	2.9	3.43E-08	Lm pro									
LmjF09.1520	hypothetical	3.2	4.11E-08	Lm pro									
LmjF10.0030	phosphate-repressible phosphate permease c	1.8	1.04E-02	Lm pro									
LmjF10.0185 g	hypothetical	2.0	1.23E-04	Lm pro									
LmjF10.0195	hypothetical	1.7	1.66E-05	Lm pro									
LmjF10.0219	hypothetical	1.8	5.98E-05	Lm pro									
LmjF10.0260	hypothetical	4.9	9.51E-08	Lm pro									
LmjF10.0460	GP63, leishmanolysin c	4.7	1.93E-08	Lm pro									
LmjF10.0465	GP63, leishmanolysin c	4.7	1.93E-08	Lm pro									
LmjF10.0480	GP63, leishmanolysin c	4.7	1.93E-08	Lm pro									
LmjF10.0490	mitogen-activated protein kinase 3	2.0	5.54E-07	Lm pro									
LmjF10.0870	Histone 3 c	2.2	6.38E-06	Lm pro									
LmjF10.0900	hypothetical	2.2	7.24E-05	Lm pro									
LmjF10.0970	Histone 3 c	2.2	6.38E-06	Lm pro									
LmjF10.0990	Histone 3 c	2.2	6.38E-06	Lm pro									
LmjF10.1190	flagellar protofilament ribbon	1.7	9.78E-06	Lm pro									
LmjF10.1300	phosphate-repressible phosphate permease c	1.8	1.04E-02	Lm pro									
LmjF11.0100	seryl-tRNA synthetase	1.9	7.98E-06	Lm pro									
LmjF11.0260	hypothetical	1.9	2.34E-05	Lm pro									

LmjF11.0310	hypothetical	1.9	1.27E-05	Lm pro									
LmjF11.0550	nucleoside transporter	2.9	5.58E-08	Lm pro									
LmjF11.0670	hypothetical	3.2	3.97E-09	Lm pro									
LmjF11.0673	hypothetical	3.2	3.97E-09	Lm pro									
LmjF11.0675	hypothetical	3.2	3.97E-09	Lm pro									
LmjF11.0740	hypothetical	1.7	8.32E-05	Lm pro									
LmjF11.0810	hypothetical	1.9	1.46E-06	Lm pro									
LmjF11.0890	hypothetical	2.2	1.56E-06	Lm pro									
LmjF11.0940	hypothetical	1.9	2.45E-05	Lm pro									
LmjF11.1310	hypothetical	2.3	1.81E-04	Lm pro									
LmjF12.0730	surface antigen protein 2 e	2.1	2.18E-05	Lm pro									
LmjF12.0740	surface antigen protein 2 e	2.0	2.14E-05	Lm pro									
LmjF12.0755	surface antigen protein 2 e	2.1	2.18E-05	Lm pro									
LmjF12.0760	surface antigen protein 2 e	2.2	1.05E-04	Lm pro									
LmjF12.0780	surface antigen protein 2 e	1.9	3.77E-05	Lm pro									
LmjF12.0810	surface antigen protein 2 e	1.9	3.77E-05	Lm pro									
LmjF12.0830	surface antigen protein 2 e	2.1	2.18E-05	Lm pro									
LmjF12.0850	surface antigen protein 2 e	2.0	6.94E-06	Lm pro									
LmjF12.0860	surface antigen protein 2 e	2.0	2.14E-05	Lm pro									
LmjF12.0870	surface antigen protein 2 e	2.0	2.59E-04	Lm pro									
LmjF12.0890	surface antigen protein 2 e	2.0	2.59E-04	Lm pro									
LmjF12.0910	surface antigen protein 2 e	2.1	1.04E-04	Lm pro									
LmjF12.0920	surface antigen protein 2 e	2.1	1.04E-04	Lm pro									
LmjF12.0940	surface antigen protein 2 e	1.9	3.77E-05	Lm pro									
LmjF12.0960	surface antigen protein 2 e	2.1	2.18E-05	Lm pro									
LmjF12.0980	surface antigen protein 2 e	1.9	3.77E-05	Lm pro									
LmjF12.0990	surface antigen protein 2 e	2.1	2.18E-05	Lm pro									
LmjF12.1000	surface antigen protein 2 e	1.9	3.77E-05	Lm pro									
LmjF12.1020	surface antigen protein 2 e	2.0	1.65E-05	Lm pro									
LmjF12.1040	surface antigen protein 2 e	2.0	2.14E-05	Lm pro									
LmjF12.1060	surface antigen protein 2 e	2.0	2.14E-05	Lm pro									
LmjF12.1070	surface antigen protein 2 e	2.0	6.94E-06	Lm pro									
LmjF13.0090	carboxypeptidase	2.1	1.56E-06	Lm pro									
LmjF13.0870	mitochondrial processing peptidase alpha subunit	2.1	4.93E-06	Lm pro									
LmjF13.1050	hypothetical	1.8	2.46E-05	Lm pro									
LmjF13.1210	nucleobase/nucleoside transporter 8.1	3.5	1.26E-08	Lm pro									
LmjF13.1470	hypothetical	1.7	7.79E-05	Lm pro									
LmjF13.1680	pyrroline-5-carboxylate reductase	2.1	2.21E-06	Lm pro									
LmjF14.0040	j-binding protein	2.3	2.27E-06	Lm pro									
LmjF14.0480	hypothetical	2.6	3.49E-08	Lm pro									
LmjF14.0490	hypothetical	2.6	3.49E-08	Lm pro									
LmjF14.0640	fatty acid elongase	1.8	5.23E-04	Lm pro									
LmjF14.0650	fatty acid elongase	2.7	1.00E-08	Lm pro									
LmjF14.0660	fatty acid elongase	2.1	1.47E-06	Lm pro									
LmjF14.0810	kinesin	1.8	1.37E-05	Lm pro									
LmjF14.1020	hypothetical	1.8	1.20E-05	Lm pro									
LmjF14.1220	hypothetical	2.5	3.85E-07	Lm pro									
LmjF14.1390	hypothetical	1.9	1.01E-03	Lm pro									
LmjF14.1430	hypothetical	2.1	7.00E-07	Lm pro									
LmjF15.0230	lysyl-tRNA synthetase	1.9	1.52E-06	Lm pro									
LmjF15.0250	hypothetical	2.4	3.01E-07	Lm pro									
LmjF15.0540	hypothetical	1.9	1.64E-06	Lm pro									
LmjF15.0930	calmodulin	1.7	3.31E-05	Lm pro									
LmjF15.1230	nucleoside transporter 1 c	5.6	6.21E-08	Lm pro									
LmjF15.1240	nucleoside transporter 1 c	5.6	6.21E-08	Lm pro									
LmjF15.1280	hypothetical	2.0	1.82E-04	Lm pro									
LmjF15.1480	cAMP specific phosphodiesterase	2.3	6.29E-06	Lm pro									
LmjF16.0210	mitochondrial ornithine transporter 1-like	2.2	4.18E-07	Lm pro									
LmjF16.0470	hypothetical	1.8	1.17E-04	Lm pro									
LmjF16.0570	Histone 3 c	2.2	6.38E-06	Lm pro									
LmjF16.0575	Histone 3 c	2.2	6.38E-06	Lm pro									
LmjF16.0600	Histone 3 c	2.2	6.38E-06	Lm pro									
LmjF16.0610	Histone 3 c	2.2	6.38E-06	Lm pro									
LmjF16.0900	hypothetical	2.2	7.00E-07	Lm pro									
LmjF16.1425	PFR2C c	5.3	2.40E-10	Lm pro									
LmjF16.1430	PFR2C c	5.3	2.40E-10	Lm pro									
LmjF16.1550	hypothetical	2.5	3.96E-07	Lm pro									
LmjF17.0180	receptor-type adenylate cyclase b	2.1	2.04E-05	Lm pro									
LmjF17.0237	receptor-type adenylate cyclase b c	2.6	1.27E-06	Lm pro									
LmjF17.0630	hypothetical	2.5	3.43E-08	Lm pro									
LmjF17.0670	protein kinase	1.8	3.03E-06	Lm pro									
LmjF17.0890	hypothetical	6.5	2.29E-10	Lm pro									

LmjF18.0090	alpha glucosidase II subunit	1.7	1.13E-04	Lm pro									
LmjF18.0200	UDP-galactopyranose mutase	1.7	5.30E-05	Lm pro									
LmjF18.0570	hypothetical	1.9	6.47E-06	Lm pro									
LmjF18.0640	serine/threonine kinase	1.8	5.59E-06	Lm pro									
LmjF18.1010	dynein light chain 2b	1.8	8.26E-05	Lm pro									
LmjF18.1080	protein kinase A catalytic subunit	3.2	4.23E-09	Lm pro									
LmjF18.1320	hypothetical	1.9	1.46E-06	Lm pro									
LmjF18.1340	hypothetical	2.6	2.35E-04	Lm pro									
LmjF18.1580	nonspecific nucleoside hydrolase	2.1	5.71E-05	Lm pro									
LmjF19.0080	hypothetical	3.5	1.10E-08	Lm pro									
LmjF19.0330	hypothetical	1.8	4.50E-06	Lm pro									
LmjF19.0530	hypothetical	2.2	1.85E-04	Lm pro									
LmjF19.0560	hypothetical	2.0	5.39E-05	Lm pro									
LmjF19.0600	hypothetical	2.1	6.62E-06	Lm pro									
LmjF19.0820	microtubule associated protein d	2.4	2.25E-06	Lm pro									
LmjF19.0842	microtubule associated protein d	2.4	2.25E-06	Lm pro									
LmjF19.0846	microtubule associated protein d	2.4	2.25E-06	Lm pro									
LmjF19.0850	microtubule associated protein d	2.4	2.25E-06	Lm pro									
LmjF19.0860	microtubule associated protein d	2.4	1.88E-06	Lm pro									
LmjF19.0870	microtubule associated protein d	2.4	1.88E-06	Lm pro									
LmjF19.0880	microtubule associated protein d	2.4	2.25E-06	Lm pro									
LmjF19.0900	microtubule associated protein d	2.4	1.88E-06	Lm pro									
LmjF19.0910	microtubule associated protein d	2.4	2.25E-06	Lm pro									
LmjF19.0920	pteridine transporter	1.9	5.09E-05	Lm pro									
LmjF19.1340	glycerol uptake protein c	1.8	3.72E-05	Lm pro									
LmjF19.1345	glycerol uptake protein c	1.8	3.72E-05	Lm pro									
LmjF19.1360	hypothetical	1.7	1.88E-02	Lm pro									
LmjF20.0150	hypothetical	1.7	7.78E-06	Lm pro									
LmjF20.1190	calpain-like cysteine peptidase	2.6	9.84E-07	Lm pro									
LmjF20.1310	calpain-like cysteine peptidase	2.9	5.07E-08	Lm pro									
LmjF20.1400	axoneme central apparatus protein	1.7	6.65E-05	Lm pro									
LmjF21.0130	protein kinase	2.2	1.24E-06	Lm pro									
LmjF21.0180	hypothetical	2.3	5.48E-06	Lm pro									
LmjF21.0240	hexokinase c	2.2	3.82E-07	Lm pro									
LmjF21.0250	hexokinase c	2.2	3.82E-07	Lm pro									
LmjF21.0440	ubiquitin-conjugating enzyme-like	2.2	7.65E-08	Lm pro									
LmjF21.1040	kinesin	1.9	1.81E-04	Lm pro									
LmjF22.0190	hypothetical	1.7	2.10E-05	Lm pro									
LmjF22.0230	amino acid permease	2.7	5.47E-07	Lm pro									
LmjF22.0440	hypothetical	1.9	9.62E-04	Lm pro									
LmjF22.0900	hypothetical	2.0	3.46E-05	Lm pro									
LmjF22.1280	hypothetical	2.2	1.01E-06	Lm pro									
LmjF22.1360	farnesyl pyrophosphate synthase	1.9	2.70E-06	Lm pro									
LmjF22.1400	hypothetical	2.1	1.46E-06	Lm pro									
LmjF23.0160	hypothetical	2.0	1.03E-02	Lm pro									
LmjF23.0360	NADP-dependent alcohol dehydrogenase	1.8	2.10E-05	Lm pro									
LmjF23.0720	hypothetical	2.4	1.42E-07	Lm pro									
LmjF23.0840	hypothetical	2.6	1.44E-07	Lm pro									
LmjF23.1086	SHERP c	1.7	5.01E-06	Lm pro									
LmjF23.1170	membrane-bound acid phosphatase 2	3.0	7.89E-05	Lm pro									
LmjF23.1440	hypothetical	2.2	1.14E-06	Lm pro									
LmjF23.1690	hypothetical	1.8	5.61E-06	Lm pro									
LmjF24.0160	hypothetical	2.2	5.45E-07	Lm pro									
LmjF24.0280	dynein intermediate-chain-like protein	1.8	7.11E-06	Lm pro									
LmjF24.0360	CMP-sialic acid transporter	1.7	1.02E-03	Lm pro									
LmjF24.0680	sugar transporter	3.9	3.52E-06	Lm pro									
LmjF24.0840	dynein arm light chain	2.1	1.10E-04	Lm pro									
LmjF24.0870	hypothetical	2.0	3.92E-02	Lm pro									
LmjF24.1090	hypothetical	4.6	4.76E-10	Lm pro									
LmjF25.0160	hypothetical	1.7	5.27E-05	Lm pro									
LmjF25.0260	dynein light chain 1	1.7	6.24E-05	Lm pro									
LmjF25.0770	hypothetical	1.7	1.71E-04	Lm pro									
LmjF25.0980	dynein heavy chain	1.9	1.36E-05	Lm pro									
LmjF25.1443	hypothetical	1.7	2.50E-04	Lm pro									
LmjF25.1600	hypothetical	1.9	5.94E-06	Lm pro									
LmjF25.1620	hypothetical	1.7	5.97E-06	Lm pro									
LmjF25.1750	hypothetical	2.0	6.73E-07	Lm pro									
LmjF25.1820	hypothetical	2.1	4.79E-06	Lm pro									
LmjF25.1920	hypothetical	1.9	1.16E-04	Lm pro									
LmjF25.2010	hypothetical	2.7	1.27E-07	Lm pro									
LmjF25.2200	hypothetical	1.8	2.08E-03	Lm pro									
LmjF26.0650	hypothetical	1.7	4.90E-04	Lm pro									

LmjF26.1450	hypothetical	2.0	9.00E-06	Lm pro									
LmjF26.1570	thimet oligopeptidase	1.9	1.27E-03	Lm pro									
LmjF26.1980	hypothetical	1.9	5.98E-05	Lm pro									
LmjF26.2570	protein kinase	4.3	3.63E-08	Lm pro									
LmjF27.0240	kinetoplast-associated protein	1.9	1.11E-05	Lm pro									
LmjF27.0510	calpain-like cysteine peptidase	2.8	1.46E-08	Lm pro									
LmjF27.0680	amino acid permease	3.0	6.85E-09	Lm pro									
LmjF27.1470	hypothetical	1.9	6.72E-06	Lm pro									
LmjF27.1480	hypothetical	2.2	2.04E-05	Lm pro									
LmjF27.1870	trypanothione synthetase	1.8	9.06E-05	Lm pro									
LmjF27.2000	hypothetical	1.8	4.59E-06	Lm pro									
LmjF27.2660	peptidyl dipeptidase c	2.5	1.95E-08	Lm pro									
LmjF28.0090	adenylate cyclase	1.7	2.97E-03	Lm pro									
LmjF28.0140	pantothenate kinase subunit	2.2	3.02E-05	Lm pro									
LmjF28.0220	hypothetical	1.7	1.91E-05	Lm pro									
LmjF28.0280	hypothetical	1.8	3.44E-05	Lm pro									
LmjF28.0740	hypothetical	1.7	7.18E-05	Lm pro									
LmjF28.0950	hypothetical	1.8	9.72E-05	Lm pro									
LmjF28.1110	hypothetical	1.8	4.25E-04	Lm pro									
LmjF28.2890	hypothetical	1.9	4.90E-04	Lm pro									
LmjF29.0280	D-lactate dehydrogenase	3.0	3.97E-09	Lm pro									
LmjF29.0760	lipophosphoglycan biosynthetic protein	2.3	3.83E-06	Lm pro									
LmjF29.0880	ADP ribosylation factor 3	1.7	3.02E-05	Lm pro									
LmjF29.0890	hypothetical	2.0	2.36E-04	Lm pro									
LmjF29.0990	hypothetical	2.0	9.44E-06	Lm pro									
LmjF29.1370	RNA binding protein	1.7	9.63E-05	Lm pro									
LmjF29.1410	hypothetical	1.9	2.10E-05	Lm pro									
LmjF29.1570	glutamamyl carboxypeptidase	1.7	7.13E-06	Lm pro									
LmjF29.1680	hypothetical	2.9	2.46E-07	Lm pro									
LmjF29.1690	hypothetical	2.8	1.27E-07	Lm pro									
LmjF29.1770	PFR1D c	4.5	4.39E-09	Lm pro									
LmjF29.2440	hypothetical	3.3	4.88E-08	Lm pro									
LmjF29.2510	6-phospho-1-fructokinase	2.6	3.62E-08	Lm pro									
LmjF29.2810	ribonuclease inhibitor-like	1.9	8.55E-06	Lm pro									
LmjF30.0020	cyclophilin	2.0	2.34E-05	Lm pro									
LmjF30.0880	adenosine kinase	1.7	4.30E-05	Lm pro									
LmjF30.0900	hypothetical	2.3	2.92E-06	Lm pro									
LmjF30.1240	calcium-binding protein	1.7	5.28E-03	Lm pro									
LmjF30.1380	n-acyl-l-amino acid amidohydrolase	1.7	5.08E-05	Lm pro									
LmjF30.2040	calpain-like cysteine peptidase	1.9	2.75E-04	Lm pro									
LmjF30.2620	hypothetical	2.2	1.55E-05	Lm pro									
LmjF30.2650	hypothetical	1.8	2.05E-03	Lm pro									
LmjF30.2700	hypothetical	3.3	6.19E-10	Lm pro									
LmjF30.2970	glyceraldehyde 3-phosphate dehydrogenase b c	2.2	4.98E-07	Lm pro									
LmjF30.2980	glyceraldehyde 3-phosphate dehydrogenase c	2.2	4.98E-07	Lm pro									
LmjF30.3190	3-hydroxy-3-methylglutaryl-CoA reductase	1.9	4.78E-06	Lm pro									
LmjF30.3240	glutamyl-tRNA synthetase	1.7	4.93E-05	Lm pro									
LmjF30.3360	calmodulin	2.0	1.70E-05	Lm pro									
LmjF31.0320	amino acid transporter	3.0	6.85E-09	Lm pro									
LmjF31.0410	calpain-like cysteine peptidase	1.8	9.30E-05	Lm pro									
LmjF31.0560	mevalonate kinase	2.1	2.37E-06	Lm pro									
LmjF31.0580	amino acid transporter aATP11	3.6	2.66E-06	Lm pro									
LmjF31.0730	hypothetical	2.1	5.79E-07	Lm pro									
LmjF31.0920	sodium stibogluconate resistance protein c	2.4	7.32E-07	Lm pro									
LmjF31.0930	sodium stibogluconate resistance protein c	2.4	7.32E-07	Lm pro									
LmjF31.0950	sodium stibogluconate resistance protein c	2.4	7.32E-07	Lm pro									
LmjF31.0960	sodium stibogluconate resistance protein c	2.4	7.32E-07	Lm pro									
LmjF31.1130	n-acyl-l-amino acid amidohydrolase	2.1	1.33E-06	Lm pro									
LmjF31.1160	hypothetical	1.9	6.38E-05	Lm pro									
LmjF31.1470	hypothetical	1.9	1.03E-04	Lm pro									
LmjF31.1800	amino acid permease	1.8	1.34E-03	Lm pro									
LmjF31.2150	prostaglandin f2-alpha synthase	5.1	3.01E-10	Lm pro									
LmjF31.2960	serine/threonine-protein kinase	2.8	5.51E-07	Lm pro									
LmjF31.2970	acetyl-CoA carboxylase	1.7	8.92E-05	Lm pro									
LmjF31.3120	phosphatidylethanolaminen-methyltransferase	2.4	4.92E-07	Lm pro									
LmjF32.0160	hypothetical	2.1	1.49E-05	Lm pro									
LmjF32.0470	hypothetical	2.0	6.72E-06	Lm pro									
LmjF32.0477	hypothetical	2.4	3.22E-05	Lm pro									
LmjF32.0730	hypothetical	2.1	3.03E-06	Lm pro									
LmjF32.0780	protein kinase	1.8	2.33E-05	Lm pro									
LmjF32.1160	long chain polyunsaturated fatty acid elongation enz	2.1	3.00E-03	Lm pro									
LmjF32.1680	hypothetical	3.3	1.03E-08	Lm pro									

LmjF32.1930	hypothetical	1.7	2.34E-04	Lm pro									
LmjF32.2040	hypothetical	2.2	5.76E-04	Lm pro									
LmjF32.2550	AMP deaminase	1.8	1.78E-03	Lm pro									
LmjF32.2660	amino acid transporter	2.6	8.56E-07	Lm pro									
LmjF32.2900	hypothetical	1.7	2.62E-05	Lm pro									
LmjF32.3030	chaperone protein DNAJ	2.2	7.70E-07	Lm pro									
LmjF32.3150	hypothetical	2.3	3.30E-07	Lm pro									
LmjF32.3250	mitogen-activated protein kinase	2.0	1.69E-05	Lm pro									
LmjF32.3310	dihydrolipoamide dehydrogenase	2.2	4.29E-06	Lm pro									
LmjF32.3450	hypothetical	1.9	2.25E-06	Lm pro									
LmjF32.3760	hypothetical	1.8	2.79E-03	Lm pro									
LmjF32.3870	myosin heavy chain	3.5	1.00E-08	Lm pro									
LmjF33.0110	hypothetical	1.8	4.91E-06	Lm pro									
LmjF33.0210	hypothetical	4.0	2.40E-10	Lm pro									
LmjF33.0590	hypothetical	1.8	1.68E-04	Lm pro									
LmjF33.0990	hypothetical	3.4	3.18E-09	Lm pro									
LmjF33.1070	hypothetical	2.7	1.88E-08	Lm pro									
LmjF33.1170	hypothetical	2.1	2.17E-04	Lm pro									
LmjF33.1460	hypothetical	2.2	1.53E-05	Lm pro									
LmjF33.1720	hypothetical	1.7	6.70E-03	Lm pro									
LmjF33.2540	carboxypeptidase	3.0	1.57E-08	Lm pro									
LmjF33.2800	hypothetical	1.7	3.20E-05	Lm pro									
LmjF33.2940	hypothetical	1.7	1.01E-03	Lm pro									
LmjF33.3100	hypothetical	1.7	3.13E-05	Lm pro									
LmjF33.3170	hypothetical	1.9	1.67E-04	Lm pro									
LmjF33.3240	Histone 1	1.9	4.39E-06	Lm pro									
LmjF34.0190	hypothetical	2.1	2.70E-05	Lm pro									
LmjF34.0410	hypothetical	2.4	8.00E-05	Lm pro									
LmjF34.0460	hypothetical	2.0	1.05E-06	Lm pro									
LmjF34.0705	hypothetical	1.8	1.81E-04	Lm pro									
LmjF34.0810	serine/threonine-protein phosphatase PP1	1.8	2.73E-06	Lm pro									
LmjF34.0970	amastin	3.5	8.14E-05	Lm pro									
LmjF34.1030	hypothetical	1.8	4.88E-04	Lm pro									
LmjF34.1230	hypothetical	1.9	2.17E-04	Lm pro									
LmjF34.1380	hypothetical	2.3	3.49E-06	Lm pro									
LmjF34.1410	d-isomer specific 2-hydroxyacid dehydrogenase-like	2.3	1.34E-06	Lm pro									
LmjF34.2000	pyroglutamyl-peptidase (PGP)	2.0	5.74E-07	Lm pro									
LmjF34.2190	dual specificity protein phosphatase	1.9	4.60E-06	Lm pro									
LmjF34.2340	asparaginyl-tRNA synthetase	1.9	1.32E-05	Lm pro									
LmjF34.2730	gpi transamidase component	1.7	2.53E-04	Lm pro									
LmjF34.2820	regulatory subunit of protein kinase a-like protein	2.2	1.03E-06	Lm pro									
LmjF34.3050	hypothetical	1.8	1.72E-06	Lm pro									
LmjF34.3480	hypothetical	1.8	1.13E-04	Lm pro									
LmjF34.4280	hypothetical	2.4	1.38E-07	Lm pro									
LmjF34.4580	hypothetical	1.8	5.01E-06	Lm pro									
LmjF34.4620	hypothetical	1.9	3.45E-07	Lm pro									
LmjF35.0640	beta-fructofuranosidase	5.9	3.52E-11	Lm pro									
LmjF35.0650	hypothetical	3.4	3.78E-07	Lm pro									
LmjF35.0970	aldose 1-epimerase	3.2	1.09E-09	Lm pro									
LmjF35.1180	NADH-dependent fumarate reductase	1.8	3.46E-05	Lm pro									
LmjF35.1320	hypothetical	1.9	1.09E-05	Lm pro									
LmjF35.1460	hypothetical	1.7	5.94E-06	Lm pro									
LmjF35.2160	adenosine deaminase	4.6	2.51E-10	Lm pro									
LmjF35.2400	hypothetical	2.2	1.19E-07	Lm pro									
LmjF35.2740	galactokinase-like	2.0	5.22E-07	Lm pro									
LmjF35.3170	protein kinase	2.1	5.62E-05	Lm pro									
LmjF35.4010	protein kinase A catalytic subunit isoform 1	1.7	5.97E-05	Lm pro									
LmjF35.4060	hypothetical	1.7	2.35E-04	Lm pro									
LmjF35.4350	hypothetical	1.7	1.25E-05	Lm pro									
LmjF35.4490	hypothetical	1.7	1.78E-04	Lm pro									
LmjF35.5080	hypothetical	1.7	2.15E-05	Lm pro									
LmjF35.5150	biopterin transporter	2.4	5.33E-07	Lm pro									
LmjF35.5250	glycosyl transferase	1.7	1.10E-04	Lm pro									
LmjF36.0350	hypothetical	1.7	2.45E-04	Lm pro									
LmjF36.0670	hypothetical	2.5	6.65E-07	Lm pro									
LmjF36.0770	hypothetical	1.8	1.94E-05	Lm pro									
LmjF36.0950	dynein heavy chain	2.6	6.28E-08	Lm pro									
LmjF36.1000	hypothetical	2.1	7.26E-06	Lm pro									
LmjF36.1130	hypothetical	1.7	1.81E-04	Lm pro									
LmjF36.1170	hypothetical	2.1	2.14E-06	Lm pro									
LmjF36.1360	adenylate kinase	2.2	4.06E-05	Lm pro									
LmjF36.1400	hypothetical	1.9	1.23E-06	Lm pro									

LmjF36.1460	hypothetical	1.7	7.89E-05	Lm pro									
LmjF36.1940	nucleoside transporter 1	2.4	8.89E-07	Lm pro									
LmjF36.2010	hypothetical	1.7	5.78E-03	Lm pro									
LmjF36.2070	hypothetical	1.9	2.91E-06	Lm pro									
LmjF36.2160	hypothetical	1.7	1.24E-04	Lm pro									
LmjF36.2360	tyrosine aminotransferase	2.7	1.59E-07	Lm pro									
LmjF36.2470	acid phophatase	1.9	5.61E-06	Lm pro									
LmjF36.2590	membrane-bound acid phosphatase 2	4.6	3.90E-11	Lm pro									
LmjF36.2610	folylpolyglutamate synthetase	2.1	5.13E-07	Lm pro									
LmjF36.2790	hypothetical	2.5	5.93E-06	Lm pro									
LmjF36.3120	hypothetical	1.9	5.54E-06	Lm pro									
LmjF36.3180	receptor-type adenylate cyclase a	3.3	2.55E-07	Lm pro									
LmjF36.3300	hypothetical	2.2	1.53E-04	Lm pro									
LmjF36.3700	hypothetical	1.8	2.92E-05	Lm pro									
LmjF36.3840	glycyl tRNA synthetase	1.9	3.75E-06	Lm pro									
LmjF36.3940	hypothetical	2.2	1.11E-07	Lm pro									
LmjF36.4260	hypothetical	2.0	1.07E-06	Lm pro									
LmjF36.5480	hypothetical	1.7	4.05E-04	Lm pro									
LmjF36.5620	isoleucyl-tRNA synthetase	1.7	4.39E-03	Lm pro									
LmjF36.6280	glucose transporter, lmgt2 c	5.6	1.91E-09	Lm pro									
LmjF36.6290	glucose transporter, lmgt2 c	5.6	1.91E-09	Lm pro									
LmjF36.6300	glucose transporter	1.9	2.42E-05	Lm pro									
LmjF36.6750	prolyl oligopeptidase	1.7	4.49E-04	Lm pro									
LmjF36.6760	hypothetical	1.7	5.43E-05	Lm pro									
LmjF01.0280					LinJ01_V3.0280	1.9	9.95E-06	Li ama					
LmjF01.0360					LinJ01_V3.0380	3.3	1.74E-08	Li ama					
LmjF01.0590					LinJ01_V3.0610	2.1	5.58E-07	Li ama					
LmjF01.0800					LinJ01_V3.0820	2.3	6.02E-03	Li ama					
LmjF02.0190					LinJ02_V3.0160	22.0	4.29E-12	Li ama					
LmjF02.0660					LinJ02_V3.0630	1.9	4.61E-03	Li ama					
LmjF02.0730					LinJ02_V3.0700	2.0	1.02E-06	Li ama					
LmjF03.0070					LinJ03_V3.0060	3.0	2.97E-06	Li ama					
LmjF03.0340					LinJ03_V3.0320	3.5	3.15E-04	Li ama					
LmjF03.0570					LinJ03_V3.0550	11.7	2.43E-12	Li ama					
LmjF03.0710					LinJ03_V3.0690	1.8	3.25E-04	Li ama					
LmjF03.0720					LinJ03_V3.0700	1.9	1.03E-04	Li ama					
LmjF04.0090					LinJ04_V3.0100	1.7	7.80E-06	Li ama					
LmjF04.1100					LinJ04_V3.1110	3.8	1.23E-05	Li ama					
LmjF04.1140					LinJ04_V3.1150	7.7	1.25E-11	Li ama					
LmjF05.0480					LinJ05_V3.0480	1.7	1.69E-02	Li ama					
LmjF05.0490					LinJ05_V3.0490	3.2	2.30E-04	Li ama					
LmjF05.0520					LinJ05_V3.0520	2.1	1.52E-06	Li ama					
LmjF06.0750					LinJ06_V3.0780	1.8	1.23E-02	Li ama					
LmjF06.0830					LinJ06_V3.0860	1.8	1.46E-05	Li ama					
LmjF06.0870					LinJ06_V3.0900	1.7	1.06E-03	Li ama					
LmjF06.1170					LinJ06_V3.1210	1.9	5.57E-07	Li ama					
LmjF06.1260					LinJ06_V3.1320	2.8	1.41E-07	Li ama					
LmjF07.0180					LinJ07_V3.0340	7.3	9.81E-07	Li ama					
LmjF07.0230					LinJ07_V3.0390	2.1	5.15E-07	Li ama					
LmjF07.1100					LinJ07_V3.1270	2.1	2.72E-05	Li ama					
LmjF07.1105					LinJ07_V3.1280	2.1	4.19E-04	Li ama					
LmjF08.0090					LinJ08_V3.0090	9.5	3.19E-04	Li ama					
LmjF08.0160					LinJ08_V3.0170	1.9	3.18E-07	Li ama					
LmjF08.0860					LinJ08_V3.0800	1.9	6.67E-07	Li ama					
LmjF09.0100					LinJ09_V3.0120	2.2	1.82E-05	Li ama					
LmjF11.0040					LinJ11_V3.0040	2.6	3.76E-06	Li ama					
LmjF11.0150					LinJ11_V3.0150	2.3	2.27E-04	Li ama					
LmjF11.0660					LinJ11_V3.0670	1.8	7.24E-03	Li ama					
LmjF11.0710					LinJ11_V3.0720	2.8	7.57E-04	Li ama					
LmjF11.1200					LinJ11_V3.1190	10.9	1.41E-09	Li ama					
LmjF12.0470					LinJ12_V3.0430	1.8	7.46E-04	Li ama					
LmjF12.0560					LinJ12_V3.0520	3.9	1.58E-08	Li ama					
LmjF12.1110					LinJ12_V3.0710	1.8	1.40E-06	Li ama					
LmjF13.0230					LinJ13_V3.0230	2.7	6.65E-09	Li ama					
LmjF13.0840					LinJ13_V3.0730	2.5	1.93E-06	Li ama					
LmjF13.0970					LinJ13_V3.0860	2.1	3.69E-05	Li ama					
LmjF13.1040					LinJ13_V3.0940	2.2	5.25E-07	Li ama					
LmjF13.1330					LinJ13_V3.1230	4.2	4.11E-03	Li ama					
LmjF13.1450					LinJ13_V3.1490	1.9	3.50E-03	Li ama					
LmjF13.1560					LinJ13_V3.1620	1.7	2.05E-06	Li ama					
LmjF13.1600					LinJ13_V3.1340	3.6	2.63E-05	Li ama					
LmjF14.0270					LinJ14_V3.0270	3.5	5.45E-07	Li ama					

LmjF14.0550					LinJ14_V3.0560	24.6	2.20E-10	Li ama					
LmjF14.0750					LinJ14_V3.0810	3.9	1.89E-08	Li ama					
LmjF14.1140					LinJ14_V3.1210	5.7	1.85E-06	Li ama					
LmjF15.0490					LinJ15_V3.0510	8.5	2.31E-10	Li ama					
LmjF15.0595					LinJ15_V3.0620 [e]	7.1	1.28E-10	Li ama					
LmjF15.0750					LinJ15_V3.0790	1.9	5.79E-03	Li ama					
LmjF15.0940					LinJ15_V3.1000	4.7	1.48E-12	Li ama					
LmjF17.0540					LinJ17_V3.0600	2.4	4.75E-08	Li ama					
LmjF17.0550					LinJ17_V3.0610	1.7	1.00E-04	Li ama					
LmjF17.1130					LinJ17_V3.1230	2.4	9.33E-04	Li ama					
LmjF18.0120					LinJ18_V3.0120	1.9	9.36E-04	Li ama					
LmjF18.0190					LinJ18_V3.0190	2.1	2.40E-05	Li ama					
LmjF18.0240					LinJ18_V3.0240	2.0	2.06E-04	Li ama					
LmjF18.0300					LinJ18_V3.0300	3.0	3.31E-10	Li ama					
LmjF18.0550					LinJ18_V3.0550	3.5	2.77E-05	Li ama					
LmjF18.0770					LinJ18_V3.0770	1.8	3.70E-03	Li ama					
LmjF18.1390					LinJ18_V3.1370	1.7	2.43E-03	Li ama					
LmjF18.1490					LinJ18_V3.1470	1.8	2.86E-06	Li ama					
LmjF19.0160					LinJ19_V3.0150	1.7	1.25E-04	Li ama					
LmjF19.0420					LinJ19_V3.0420	5.0	2.40E-10	Li ama					
LmjF19.0660					LinJ19_V3.0660	1.9	4.54E-04	Li ama					
LmjF19.1080					LinJ19_V3.1060b	4.9	2.27E-08	Li ama					
LmjF19.1180					LinJ19_V3.1170	9.0	7.58E-11	Li ama					
LmjF20.0380					LinJ20_V3.0450	1.8	2.04E-05	Li ama					
LmjF20.0430					LinJ20_V3.0500	1.7	4.87E-02	Li ama					
LmjF20.1360					LinJ20_V3.1400	2.0	1.76E-07	Li ama					
LmjF20.1700					LinJ20_V3.1670	3.3	1.12E-09	Li ama					
LmjF20.1730					LinJ20_V3.1700	2.1	5.58E-07	Li ama					
LmjF21.0490					LinJ21_V3.0550	4.5	5.87E-10	Li ama					
LmjF21.0540					LinJ21_V3.0600	1.7	3.14E-05	Li ama					
LmjF21.0650					LinJ21_V3.0710	1.7	5.04E-02	Li ama					
LmjF21.0825					LinJ21_V3.0920	4.6	1.48E-11	Li ama					
LmjF21.0980					LinJ21_V3.1220	5.1	2.20E-09	Li ama					
LmjF22.0240					LinJ22_V3.0110	1.8	3.89E-07	Li ama					
LmjF22.0470					LinJ22_V3.0350	2.7	1.94E-04	Li ama					
LmjF23.1060					LinJ23_V3.1220	2.4	6.43E-05	Li ama					
LmjF23.1065					LinJ23_V3.1290	2.8	6.47E-06	Li ama					
LmjF23.1267					LinJ23_V3.1520	2.3	1.05E-05	Li ama					
LmjF23.1740					LinJ23_V3.1790	7.0	1.25E-08	Li ama					
LmjF23.1760					LinJ23_V3.1810	1.9	3.97E-02	Li ama					
LmjF24.0540					LinJ24_V3.0550	4.5	1.40E-03	Li ama					
LmjF24.0590					LinJ24_V3.0600	2.1	6.91E-04	Li ama					
LmjF24.0670					LinJ24_V3.0680	2.2	1.94E-04	Li ama					
LmjF24.0690					LinJ24_V3.0700	8.2	8.18E-12	Li ama					
LmjF24.0930					LinJ24_V3.0950	2.3	4.35E-04	Li ama					
LmjF24.1060					LinJ24_V3.1080	4.0	2.13E-10	Li ama					
LmjF24.1290					LinJ24_V3.1320	1.8	1.12E-04	Li ama					
LmjF24.1370					LinJ24_V3.1410	1.7	8.49E-03	Li ama					
LmjF24.1420					LinJ24_V3.1460	1.9	2.58E-05	Li ama					
LmjF24.1550					LinJ24_V3.1620	1.9	1.33E-03	Li ama					
LmjF24.1580					LinJ24_V3.1650	1.7	4.91E-04	Li ama					
LmjF25.0020					LinJ25_V3.0020	1.7	4.82E-04	Li ama					
LmjF25.0210					LinJ25_V3.0210	1.7	1.16E-03	Li ama					
LmjF25.0220					LinJ25_V3.0220	11.0	1.92E-11	Li ama					
LmjF25.0390					LinJ25_V3.0400	1.8	1.50E-06	Li ama					
LmjF25.0430					LinJ25_V3.0440	2.6	3.66E-07	Li ama					
LmjF25.0450					LinJ25_V3.0460	5.0	2.40E-10	Li ama					
LmjF25.0510					LinJ25_V3.0520	1.9	3.26E-04	Li ama					
LmjF25.0550					LinJ25_V3.0560	32.7	1.78E-11	Li ama					
LmjF25.0690					LinJ25_V3.0700	1.8	8.88E-05	Li ama					
LmjF25.1890					LinJ25_V3.1970	2.5	3.73E-06	Li ama					
LmjF25.1970					LinJ25_V3.2050	3.8	1.09E-09	Li ama					
LmjF26.0030					LinJ26_V3.0040	8.1	6.17E-10	Li ama					
LmjF26.0400					LinJ26_V3.0390	12.8	4.09E-09	Li ama					
LmjF26.1250					LinJ26_V3.1230	2.0	6.68E-05	Li ama					
LmjF26.1280					LinJ26_V3.1260	1.9	2.21E-05	Li ama					
LmjF26.1850					LinJ26_V3.1850	2.8	1.22E-08	Li ama					
LmjF26.2100					LinJ26_V3.2100	2.2	1.15E-06	Li ama					
LmjF26.2210					LinJ26_V3.2220	6.5	7.38E-11	Li ama					
LmjF26.2630					LinJ26_V3.2660	1.8	5.04E-05	Li ama					
LmjF27.0100					LinJ27_V3.0100	4.6	5.87E-10	Li ama					
LmjF27.1200					LinJ27_V3.1080	2.8	1.38E-05	Li ama					

LmjF27.1530					LinJ27_V3.1430	2.4	7.12E-03	Li ama					
LmjF27.1690					LinJ27_V3.1590	1.8	4.33E-04	Li ama					
LmjF27.2060					LinJ27_V3.1980	1.7	6.26E-05	Li ama					
LmjF27.2340					LinJ27_V3.2290	1.7	7.30E-06	Li ama					
LmjF27.2500					LinJ27_V3.2450	4.0	4.74E-08	Li ama					
LmjF28.0530					LinJ28_V3.0560	2.3	2.77E-05	Li ama					
LmjF28.1120					LinJ28_V3.1220	2.2	9.02E-03	Li ama					
LmjF28.1470					LinJ28_V3.1590	1.9	1.68E-05	Li ama					
LmjF28.1480					LinJ28_V3.1600	1.9	1.68E-05	Li ama					
LmjF28.1570					LinJ28_V3.1700	2.3	1.18E-07	Li ama					
LmjF28.2020					LinJ28_V3.2160	1.7	1.60E-03	Li ama					
LmjF28.2330					LinJ28_V3.2500	2.2	1.54E-06	Li ama					
LmjF28.2580					LinJ28_V3.2790	5.5	1.25E-11	Li ama					
LmjF28.2650					LinJ28_V3.2850	2.2	1.70E-04	Li ama					
LmjF29.0200					LinJ29_V3.0210	2.1	2.86E-02	Li ama					
LmjF29.0450					LinJ29_V3.0460	1.9	2.72E-04	Li ama					
LmjF29.0620					LinJ29_V3.0640	4.8	4.63E-10	Li ama					
LmjF29.0868					LinJ29_V3.0930	46.7	1.00E-14	Li ama					
LmjF29.0885					LinJ29_V3.0960	1.9	5.12E-03	Li ama					
LmjF29.0935					LinJ29_V3.1020	2.4	5.57E-09	Li ama					
LmjF29.1550					LinJ29_V3.1660	2.1	3.00E-02	Li ama					
LmjF29.1560					LinJ29_V3.1670	7.1	3.90E-13	Li ama					
LmjF29.1890					LinJ29_V3.2010	1.7	5.77E-04	Li ama					
LmjF29.2130					LinJ29_V3.2240	1.8	1.46E-04	Li ama					
LmjF29.2390					LinJ29_V3.2500	2.5	5.17E-03	Li ama					
LmjF29.2820					LinJ29_V3.2930	7.3	5.20E-10	Li ama					
LmjF30.0170					LinJ30_V3.0160	12.4	2.43E-10	Li ama					
LmjF30.0540					LinJ30_V3.0560	4.1	6.13E-10	Li ama					
LmjF30.0630					LinJ30_V3.0650	1.9	2.48E-02	Li ama					
LmjF30.0720					LinJ30_V3.0750	2.0	5.54E-03	Li ama					
LmjF30.0780					LinJ30_V3.0830	2.1	3.69E-03	Li ama					
LmjF30.1090					LinJ30_V3.1150	1.9	3.15E-04	Li ama					
LmjF30.1100					LinJ30_V3.1160	2.4	2.26E-05	Li ama					
LmjF30.1120					LinJ30_V3.1180	2.0	5.30E-05	Li ama					
LmjF30.1190					LinJ30_V3.1250	2.5	1.79E-04	Li ama					
LmjF30.1500					LinJ30_V3.1520	1.7	2.14E-04	Li ama					
LmjF30.1610					LinJ30_V3.1630	1.9	1.72E-03	Li ama					
LmjF30.2050					LinJ30_V3.2050	2.0	1.00E-14	Li ama					
LmjF30.2340					LinJ30_V3.2350	2.4	3.71E-06	Li ama					
LmjF30.2790					LinJ30_V3.2800	2.0	3.51E-05	Li ama					
LmjF30.3400					LinJ30_V3.3450	3.0	6.09E-07	Li ama					
LmjF30.3710					LinJ30_V3.3770	1.8	4.09E-04	Li ama					
LmjF31.0120					LinJ31_V3.0130	1.9	1.93E-05	Li ama					
LmjF31.0190					LinJ31_V3.0200	1.7	1.52E-04	Li ama					
LmjF31.0200					LinJ31_V3.0210	1.9	5.77E-04	Li ama					
LmjF31.0440					LinJ31_V3.0450	1.9	2.96E-05	Li ama					
LmjF31.0470					LinJ31_V3.0490	5.4	4.82E-10	Li ama					
LmjF31.0770					LinJ31_V3.0800	1.7	1.15E-04	Li ama					
LmjF31.0850					LinJ31_V3.0880	1.7	1.15E-04	Li ama					
LmjF31.1165					LinJ31_V3.1180 [e]	2.2	3.71E-06	Li ama					
LmjF31.1190					LinJ31_V3.1210	2.1	2.73E-06	Li ama					
LmjF31.1400					LinJ31_V3.1430	2.0	2.77E-05	Li ama					
LmjF31.1600					LinJ31_V3.1630	2.2	3.55E-07	Li ama					
LmjF31.1680					LinJ31_V3.1700	4.0	5.86E-06	Li ama					
LmjF31.2620					LinJ31_V3.2690	10.9	2.31E-10	Li ama					
LmjF31.2710					LinJ31_V3.2790	7.6	7.82E-07	Li ama					
LmjF32.0090					LinJ32_V3.0090	1.7	1.53E-02	Li ama					
LmjF32.1230					LinJ32_V3.1290	2.4	1.80E-05	Li ama					
LmjF32.1350					LinJ32_V3.1410	2.2	1.63E-08	Li ama					
LmjF32.2770					LinJ32_V3.2910	1.8	3.89E-02	Li ama					
LmjF32.3440					LinJ32_V3.3640	2.3	2.09E-05	Li ama					
LmjF32.3600					LinJ32_V3.3760	2.9	4.14E-10	Li ama					
LmjF32.3860					LinJ32_V3.4010	2.3	1.46E-05	Li ama					
LmjF32.3980					LinJ32_V3.4130	2.3	1.11E-06	Li ama					
LmjF33.0565					LinJ33_V3.0590	1.8	1.56E-06	Li ama					
LmjF33.0760					LinJ33_V3.0810	2.1	1.05E-05	Li ama					
LmjF33.1575					LinJ33_V3.1670	3.2	1.25E-07	Li ama					
LmjF33.2410					LinJ33_V3.2540	1.7	1.51E-02	Li ama					
LmjF33.2570					LinJ33_V3.2700	4.7	5.40E-10	Li ama					
LmjF33.2710					LinJ33_V3.2850	1.9	2.96E-05	Li ama					
LmjF34.0175					LinJ34_V3.0190	2.3	3.99E-04	Li ama					
LmjF34.0300					LinJ34_V3.0320	2.4	7.30E-06	Li ama					

LmjF34.0430					LinJ34_V3.0450	10.2	1.27E-07	Li ama					
LmjF34.0490					LinJ34_V3.0510	1.9	5.66E-05	Li ama					
LmjF34.0530					LinJ34_V3.0550	1.8	1.51E-05	Li ama					
LmjF34.1060					LinJ34_V3.1130	3.6	2.27E-08	Li ama					
LmjF34.1970					LinJ34_V3.1160	2.3	1.71E-07	Li ama					
LmjF34.2473					LinJ34_V3.2250	2.0	1.32E-03	Li ama					
LmjF34.3640					LinJ34_V3.3420	5.7	4.30E-13	Li ama					
LmjF34.3645					LinJ34_V3.3430 [e]	5.7	4.30E-13	Li ama					
LmjF34.3730					LinJ34_V3.3520	4.1	1.56E-08	Li ama					
LmjF35.0230					LinJ35_V3.0230	2.9	7.60E-07	Li ama					
LmjF35.1640					LinJ35_V3.1640	9.4	3.03E-10	Li ama					
LmjF35.1770					LinJ35_V3.1770	2.0	1.21E-04	Li ama					
LmjF35.2450					LinJ35_V3.2500	9.1	1.93E-06	Li ama					
LmjF35.4040					LinJ35_V3.4090	10.3	4.52E-10	Li ama					
LmjF35.4380					LinJ35_V3.4450	2.0	1.56E-06	Li ama					
LmjF35.5030					LinJ35_V3.5350	2.1	9.26E-07	Li ama					
LmjF36.0120					LinJ36_V3.0130	7.3	7.03E-09	Li ama					
LmjF36.0400					LinJ36_V3.0430	3.6	1.65E-07	Li ama					
LmjF36.0440					LinJ36_V3.0470	6.3	4.22E-10	Li ama					
LmjF36.0890					LinJ36_V3.0950	1.9	3.08E-04	Li ama					
LmjF36.1160					LinJ36_V3.1220	1.9	1.25E-04	Li ama					
LmjF36.2580					LinJ36_V3.2710	2.2	3.62E-07	Li ama					
LmjF36.2640					LinJ36_V3.2770	2.1	3.22E-05	Li ama					
LmjF36.2990					LinJ36_V3.3140	1.8	2.76E-04	Li ama					
LmjF36.3235					LinJ36_V3.3390	3.0	5.52E-05	Li ama					
LmjF36.3480					LinJ36_V3.3640	4.9	2.27E-08	Li ama					
LmjF36.3900					LinJ36_V3.4090	1.7	2.58E-06	Li ama					
LmjF36.3990					LinJ36_V3.4180	8.7	1.74E-08	Li ama					
LmjF36.4200					LinJ36_V3.4410	3.5	2.43E-10	Li ama					
LmjF36.4710					LinJ36_V3.4940	1.8	3.93E-05	Li ama					
LmjF36.5320					LinJ36_V3.5550	2.6	3.11E-03	Li ama					
LmjF36.5530					LinJ36_V3.5770	1.8	3.46E-04	Li ama					
LmjF36.5630					LinJ36_V3.5880	1.7	7.08E-04	Li ama					
LmjF36.6270					LinJ36_V3.6530	5.0	1.82E-04	Li ama					
LmjF36.6580					LinJ36_V3.6890	2.1	2.26E-05	Li ama					
LmjF36.6960					LinJ36_V3.7300	2.9	1.13E-05	Li ama					
no_id					LinJ16_V3.0050	1.9	2.27E-03	Li ama					
no_id					LinJ16_V3.0100	1.9	3.37E-05	Li ama					
no_id					LinJ16_V3.0210	3.4	5.38E-08	Li ama					
no_id					LinJ16_V3.0620	5.2	2.14E-07	Li ama					
no_id					LinJ16_V3.0660	2.1	5.26E-04	Li ama					
no_id					LinJ16_V3.0850	8.9	1.00E-14	Li ama					
no_id					LinJ16_V3.0860	1.8	7.46E-04	Li ama					
no_id					LinJ16_V3.1180	1.9	9.36E-04	Li ama					
no_id					LinJ16_V3.1260	11.6	1.00E-14	Li ama					
no_id					LinJ16_V3.1460 [e]	1.9	2.78E-04	Li ama					
no_id					LinJ16_V3.1470	2.1	4.02E-05	Li ama					
no_id					LinJ22_V3.0670 [f]	5.6	3.36E-08	Li ama					
no_id					LinJ25_V3.2870	2.3	1.60E-04	Li ama					
no_id					LinJ29_V3.1450	2.0	2.67E-03	Li ama					
no_id					LinJ29_V3.3000	2.0	2.67E-03	Li ama					
no_id					LinJ29_V3.3010	1.7	8.53E-03	Li ama					
no_id					LinJ29_V3.3020	1.8	4.85E-04	Li ama					
no_id					LinJ31_V3.0470	1.7	9.89E-04	Li ama					
LmjF02.0310					LinJ02_V3.0280	1.9	3.73E-05	Li pro					
LmjF02.0460					LinJ02_V3.0430	4.3	1.28E-10	Li pro					
LmjF02.0530					LinJ02_V3.0500	2.1	4.32E-06	Li pro					
LmjF03.0940					LinJ03_V3.0920	1.7	3.59E-03	Li pro					
LmjF04.0050					LinJ04_V3.0060	2.8	9.26E-07	Li pro					
LmjF04.1170					LinJ04_V3.1190	2.2	1.72E-08	Li pro					
LmjF05.0010					LinJ05_V3.0010	1.7	4.63E-05	Li pro					
LmjF05.0100					LinJ05_V3.0100	1.9	1.96E-05	Li pro					
LmjF05.0350					LinJ05_V3.0350	1.7	3.14E-05	Li pro					
LmjF05.0370					LinJ05_V3.0370	2.1	7.42E-04	Li pro					
LmjF05.0420					LinJ05_V3.0420	1.8	2.34E-04	Li pro					
LmjF05.0780					LinJ05_V3.0780	1.7	9.44E-06	Li pro					
LmjF05.1030					LinJ05_V3.1030	2.6	1.13E-05	Li pro					
LmjF05.1110					LinJ05_V3.1110	3.0	1.82E-08	Li pro					
LmjF06.0120					LinJ06_V3.0120	1.7	7.22E-05	Li pro					
LmjF06.0140					LinJ06_V3.0140	1.7	7.64E-05	Li pro					
LmjF06.0270					LinJ06_V3.0270	1.7	2.08E-04	Li pro					
LmjF06.1290					LinJ06_V3.1350	1.7	1.84E-03	Li pro					

LmjF07.0025					LinJ07_V3.0030	2.4	4.79E-07	Li pro					
LmjF07.0820					LinJ07_V3.0980	2.4	5.45E-07	Li pro					
LmjF07.0830					LinJ07_V3.0990	1.8	5.08E-04	Li pro					
LmjF08.0010					LinJ08_V3.0010	1.9	3.99E-04	Li pro					
LmjF09.0400					LinJ09_V3.0450	2.0	1.25E-04	Li pro					
LmjF09.0410					LinJ09_V3.0460	1.9	3.20E-04	Li pro					
LmjF09.1010					LinJ09_V3.1070	1.9	4.11E-05	Li pro					
LmjF10.0935					LinJ10_V3.1000	2.3	4.21E-04	Li pro					
LmjF10.1150					LinJ10_V3.1240	2.0	9.97E-05	Li pro					
LmjF11.0350					LinJ11_V3.0350	1.8	3.14E-05	Li pro					
LmjF11.1000					LinJ11_V3.1000	1.8	5.47E-06	Li pro					
LmjF12.0310					LinJ12_V3.0260	1.7	3.68E-02	Li pro					
LmjF12.0520					LinJ12_V3.0480	1.7	4.62E-05	Li pro					
LmjF13.1110					LinJ13_V3.1010	1.8	5.07E-05	Li pro					
LmjF13.1180					LinJ13_V3.1080	1.8	2.04E-05	Li pro					
LmjF13.1340					LinJ13_V3.1240	1.8	2.52E-04	Li pro					
LmjF13.1360					LinJ13_V3.1260	1.8	2.22E-03	Li pro					
LmjF13.1520					LinJ13_V3.1580	1.8	2.75E-05	Li pro					
LmjF14.1060					LinJ14_V3.1130	1.9	4.32E-06	Li pro					
LmjF14.1120					LinJ14_V3.1190	1.7	2.58E-03	Li pro					
LmjF14.1440					LinJ14_V3.1540	2.5	1.17E-04	Li pro					
LmjF15.0740					LinJ15_V3.0780	1.9	1.65E-04	Li pro					
LmjF15.1450					LinJ15_V3.1500	1.9	9.56E-05	Li pro					
LmjF17.0280					LinJ17_V3.0330	2.3	4.80E-04	Li pro					
LmjF17.0500					LinJ17_V3.0560	1.8	2.64E-03	Li pro					
LmjF18.0510					LinJ18_V3.0510	2.0	2.58E-06	Li pro					
LmjF18.0590					LinJ18_V3.0590	1.8	4.25E-05	Li pro					
LmjF19.0540					LinJ19_V3.0570	1.7	1.25E-04	Li pro					
LmjF19.0570					LinJ19_V3.0560	2.3	6.67E-07	Li pro					
LmjF19.0610					LinJ19_V3.0610	1.8	5.39E-04	Li pro					
LmjF19.0690					LinJ19_V3.0690	1.7	7.47E-06	Li pro					
LmjF19.1160					LinJ19_V3.1150	1.9	2.48E-06	Li pro					
LmjF19.1450					LinJ19_V3.1490	1.7	2.58E-06	Li pro					
LmjF20.0030					LinJ20_V3.0030	3.6	1.12E-09	Li pro					
LmjF20.0270					LinJ20_V3.0300	2.2	1.66E-03	Li pro					
LmjF20.1040					LinJ20_V3.1050	1.8	2.49E-03	Li pro					
LmjF20.1180					LinJ20_V3.1210	1.7	2.05E-05	Li pro					
LmjF20.1310					LinJ20_V3.1350	1.7	4.81E-05	Li pro					
LmjF20.1365					LinJ20_V3.1410	1.7	1.34E-03	Li pro					
LmjF21.1210					LinJ21_V3.1450	1.7	3.28E-03	Li pro					
LmjF21.1250					LinJ21_V3.1490	2.4	2.56E-05	Li pro					
LmjF21.1555					LinJ21_V3.1830	1.7	2.35E-05	Li pro					
LmjF21.1720					LinJ21_V3.2090	1.8	1.68E-04	Li pro					
LmjF22.1150					LinJ22_V3.0970	1.9	4.97E-04	Li pro					
LmjF22.1410					LinJ22_V3.1260	1.9	3.34E-06	Li pro					
LmjF22.1460					LinJ22_V3.1310	1.9	2.81E-07	Li pro					
LmjF23.0010					LinJ23_V3.0010	2.1	1.94E-07	Li pro					
LmjF23.0080					LinJ23_V3.0090	1.7	1.60E-04	Li pro					
LmjF23.0560					LinJ23_V3.0720	1.9	8.95E-06	Li pro					
LmjF23.1000					LinJ23_V3.1170	2.2	2.18E-04	Li pro					
LmjF23.1450					LinJ23_V3.1850	1.8	1.63E-05	Li pro					
LmjF23.1520					LinJ23_V3.1920	1.9	1.62E-03	Li pro					
LmjF24.0850					LinJ24_V3.0870	1.8	2.58E-05	Li pro					
LmjF24.1315					LinJ24_V3.1350	1.7	9.44E-03	Li pro					
LmjF24.1400					LinJ24_V3.1440	2.1	1.04E-03	Li pro					
LmjF24.1570					LinJ24_V3.1640	2.1	3.09E-05	Li pro					
LmjF24.1720					LinJ24_V3.1790	1.8	8.28E-03	Li pro					
LmjF24.2310					LinJ24_V3.2400	2.4	1.17E-04	Li pro					
LmjF24.2330					LinJ24_V3.2420	1.8	6.62E-06	Li pro					
LmjF25.0490					LinJ25_V3.0500	2.1	1.24E-08	Li pro					
LmjF25.0500					LinJ25_V3.0510	2.1	1.24E-08	Li pro					
LmjF25.0540					LinJ25_V3.0550	1.8	9.02E-05	Li pro					
LmjF25.1000					LinJ25_V3.1030	1.9	1.46E-03	Li pro					
LmjF25.1470					LinJ25_V3.1530	1.8	3.46E-03	Li pro					
LmjF26.1340					LinJ26_V3.1320	1.7	2.13E-04	Li pro					
LmjF26.1960					LinJ26_V3.1960	1.8	1.11E-06	Li pro					
LmjF26.2280					LinJ26_V3.2290	1.8	5.88E-03	Li pro					
LmjF26.2660					LinJ26_V3.2690	1.8	2.96E-05	Li pro					
LmjF27.0190					LinJ27_V3.0190	1.9	1.73E-06	Li pro					
LmjF27.0350					LinJ27_V3.0360	1.8	1.05E-03	Li pro					
LmjF27.0490					LinJ27_V3.2490	1.8	4.36E-06	Li pro					
LmjF27.0500					LinJ27_V3.0510	1.9	1.59E-04	Li pro					

LmjF27.1080					LinJ27_V3.0950	2.1	1.19E-04	Li pro					
LmjF27.1110					LinJ27_V3.0980	1.8	2.10E-05	Li pro					
LmjF27.1220					LinJ27_V3.1100	1.7	3.03E-05	Li pro					
LmjF27.1230					LinJ27_V3.1110	2.5	2.82E-08	Li pro					
LmjF27.1730					LinJ27_V3.1630	1.8	1.96E-05	Li pro					
LmjF27.1850					LinJ27_V3.1750	1.7	1.08E-02	Li pro					
LmjF28.0100					LinJ28_V3.0100	1.8	1.03E-04	Li pro					
LmjF28.0130					LinJ28_V3.0130	1.7	4.52E-03	Li pro					
LmjF28.0210					LinJ28_V3.0210	1.8	1.05E-05	Li pro					
LmjF28.0890					LinJ28_V3.0980	3.1	1.10E-03	Li pro					
LmjF28.1930					LinJ28_V3.2050	2.5	2.49E-08	Li pro					
LmjF28.1930					LinJ28_V3.2060	2.5	2.49E-08	Li pro					
LmjF28.1990					LinJ28_V3.2120	1.8	4.56E-05	Li pro					
LmjF28.2880					LinJ28_V3.3110	2.0	2.12E-06	Li pro					
LmjF29.0020					LinJ29_V3.0020	1.8	2.36E-04	Li pro					
LmjF29.0510					LinJ29_V3.0520	2.0	8.09E-06	Li pro					
LmjF29.0850					LinJ29_V3.0890	2.2	1.01E-03	Li pro					
LmjF29.0960					LinJ29_V3.1050	1.8	4.29E-02	Li pro					
LmjF29.1240					LinJ29_V3.1330	1.9	1.70E-03	Li pro					
LmjF29.1300					LinJ29_V3.1390	1.7	1.33E-03	Li pro					
LmjF29.1390					LinJ29_V3.1500	1.7	6.46E-05	Li pro					
LmjF30.1810					LinJ30_V3.1810	1.9	6.85E-05	Li pro					
LmjF30.1960					LinJ30_V3.1940	1.9	7.93E-06	Li pro					
LmjF30.2370					LinJ30_V3.2380	2.0	5.33E-05	Li pro					
LmjF30.2570					LinJ30_V3.2560	1.7	4.21E-04	Li pro					
LmjF30.2900					LinJ30_V3.2920	1.8	1.40E-05	Li pro					
LmjF30.3260					LinJ30_V3.3310	1.7	2.88E-04	Li pro					
LmjF30.3310					LinJ30_V3.3360	1.8	7.97E-05	Li pro					
LmjF30.3550					LinJ30_V3.3610	2.2	2.91E-04	Li pro					
LmjF31.0710					LinJ31_V3.0740	1.7	7.83E-05	Li pro					
LmjF31.1030					LinJ31_V3.1030	1.9	9.53E-06	Li pro					
LmjF32.0230					LinJ32_V3.0240	2.1	2.64E-03	Li pro					
LmjF32.0400					LinJ32_V3.0410	1.9	7.67E-06	Li pro					
LmjF32.0500					LinJ32_V3.0530	1.7	1.56E-02	Li pro					
LmjF32.1830					LinJ32_V3.1920	1.9	1.54E-03	Li pro					
LmjF32.1840					LinJ32_V3.1930	1.8	4.03E-04	Li pro					
LmjF32.1875					LinJ32_V3.1970	1.7	7.25E-03	Li pro					
LmjF32.2020					LinJ32_V3.2150	2.2	3.42E-04	Li pro					
LmjF32.2610					LinJ32_V3.2750	1.9	6.30E-06	Li pro					
LmjF32.3320					LinJ32_V3.3520	2.2	1.25E-05	Li pro					
LmjF32.3780					LinJ32_V3.3930	2.2	7.88E-05	Li pro					
LmjF32.3790					LinJ32_V3.3940	1.7	1.01E-03	Li pro					
LmjF33.1140					LinJ33_V3.1200	2.6	5.96E-08	Li pro					
LmjF33.1450					LinJ33_V3.1540	1.7	7.24E-05	Li pro					
LmjF33.2100					LinJ33_V3.2220	2.2	3.16E-07	Li pro					
LmjF33.2440					LinJ33_V3.2570	1.8	7.05E-07	Li pro					
LmjF33.3240					LinJ33_V3.3390	2.6	3.06E-07	Li pro					
LmjF34.0070					LinJ34_V3.0070	3.0	1.94E-07	Li pro					
LmjF34.0110					LinJ34_V3.0120	1.8	1.00E-03	Li pro					
LmjF34.0200					LinJ34_V3.0220	1.9	7.38E-05	Li pro					
LmjF34.0320					LinJ34_V3.0340	1.8	2.42E-06	Li pro					
LmjF34.0550					LinJ34_V3.0570	1.7	9.36E-04	Li pro					
LmjF34.0990					LinJ34_V3.1060	1.9	1.52E-04	Li pro					
LmjF34.1420					LinJ34_V3.1520	2.0	2.71E-04	Li pro					
LmjF34.1530					LinJ34_V3.1630	1.8	2.29E-05	Li pro					
LmjF34.2560					LinJ34_V3.2390	2.2	6.21E-07	Li pro					
LmjF34.2580					LinJ34_V3.2410	2.5	4.67E-04	Li pro					
LmjF34.3120					LinJ34_V3.4290	1.7	1.72E-04	Li pro					
LmjF34.3390					LinJ34_V3.3170	1.7	3.84E-03	Li pro					
LmjF34.3500					LinJ34_V3.3280	1.7	4.92E-05	Li pro					
LmjF34.3670					LinJ34_V3.3460	1.7	1.79E-03	Li pro					
LmjF34.4070					LinJ34_V3.3900	1.7	5.00E-04	Li pro					
LmjF35.0030					LinJ35_V3.5450	1.9	4.72E-04	Li pro					
LmjF35.0180					LinJ35_V3.0180	1.8	1.10E-03	Li pro					
LmjF35.0950					LinJ35_V3.0970	1.8	1.03E-02	Li pro					
LmjF35.1030					LinJ35_V3.1050	1.8	7.77E-03	Li pro					
LmjF35.1230					LinJ35_V3.1240	2.5	2.14E-07	Li pro					
LmjF35.1300					LinJ35_V3.1310	1.7	1.58E-03	Li pro					
LmjF35.1380					LinJ35_V3.1390	2.1	7.82E-07	Li pro					
LmjF35.1755					LinJ35_V3.1750	2.0	7.11E-05	Li pro					
LmjF35.2420					LinJ35_V3.2470	1.9	4.25E-05	Li pro					
LmjF35.2430					LinJ35_V3.2480	1.9	5.49E-05	Li pro					

LmjF35.3330					LinJ35_V3.3380	1.8	5.07E-05	Li pro					
LmjF35.3340					LinJ35_V3.3390	2.0	3.19E-07	Li pro					
LmjF35.3720					LinJ35_V3.3770	1.7	7.64E-05	Li pro					
LmjF35.4290					LinJ35_V3.4360	1.7	6.65E-05	Li pro					
LmjF35.4760					LinJ35_V3.4820	1.9	1.89E-03	Li pro					
LmjF36.0500					LinJ36_V3.0530	2.1	2.85E-02	Li pro					
LmjF36.0550					LinJ36_V3.0600	2.0	7.15E-04	Li pro					
LmjF36.1380					LinJ36_V3.1430	2.3	9.65E-05	Li pro					
LmjF36.1635					LinJ36_V3.1710	1.9	7.38E-05	Li pro					
LmjF36.3090					LinJ36_V3.3240	1.8	2.06E-04	Li pro					
LmjF36.3210					LinJ36_V3.3360	1.8	4.69E-06	Li pro					
LmjF36.5290					LinJ36_V3.5520	2.9	3.85E-03	Li pro					
LmjF36.5380					LinJ36_V3.5620	2.6	3.12E-05	Li pro					
LmjF36.5820					LinJ36_V3.6070	1.8	2.88E-04	Li pro					
LmjF36.5845					LinJ36_V3.6100	2.8	2.58E-07	Li pro					
LmjF36.5920					LinJ36_V3.6180	1.8	2.06E-04	Li pro					
LmjF36.6030					LinJ36_V3.6290	1.9	6.34E-04	Li pro					
LmjF36.7000					LinJ36_V3.7360	2.5	2.09E-08	Li pro					
					LinJ16_V3.0950	2.0	1.29E-05	Li pro					
					LinJ16_V3.1500	2.0	1.56E-05	Li pro					
					LinJ16_V3.1510	3.7	1.23E-11	Li pro					
					LinJ16_V3.1520	3.7	1.23E-11	Li pro					
					LinJ16_V3.1550	1.8	8.62E-05	Li pro					
					LinJ16_V3.1570	1.8	8.62E-05	Li pro					
